# Examining State Affective and Cognitive Outcomes Following Brief Mobile Phone-Based Training Sessions to Reduce Anxious Interpretations

**DOI:** 10.1007/s10608-025-10623-z

**Published:** 2025-06-16

**Authors:** Kaitlyn Petz, Emma Toner, Mark Rucker, Emily Leventhal, Sarah Livermon, Benjamin Davidson, Mehdi Boukhechba, Laura Barnes, Bethany Teachman

**Affiliations:** 1 University of Virginia, Charlottesville, United States; 2 Johnson & Johnson (United States), New Brunswick, United States

**Keywords:** Cognitive bias modification, mHealth, Anxiety, Ecological momentary assessment, Affect

## Abstract

**Background:**

Rates of stress and anxiety are alarmingly high in university communities, but most people do not receive treatment. Mobile health (mHealth) interventions show promise to improve psychological symptoms and increase access to interventions, but little is known about their effects in the moment. The present study evaluated the short-term impact of brief mHealth sessions to determine which intervention features are associated with the greatest momentary self-reported improvements.

**Methods:**

Participants (*N* = 100 undergraduate students, graduate students, and university staff members) completed brief training sessions 1–2 times daily of Hoos Think Calmly, a new mobile application for the university community that uses Cognitive Bias Modification for Interpretations (CBM-I) to shift anxious thinking patterns. Training sessions varied based on stressor domain/topic selected and writing requirements, among other features. Linear mixed effects models were used to test whether stressor domain or writing requirements predict post-training: (1) momentary affect, (2) reappraisal self-efficacy, and (3) emotion regulation self-efficacy.

**Results:**

Self-reported improvement in state affect, reappraisal self-efficacy, and emotion regulation self-efficacy occurred for six out of eight stressor domains. Additionally, training sessions requiring less (vs. more) writing were associated with greater positive changes in affect, but not reappraisal or emotion regulation self-efficacy.

**Conclusion:**

Stressor domain and writing requirements are associated with different in-the-moment cognitive and affective outcomes, pointing to the need to tailor mHealth programs to users’ specific needs and current stressors.

## Introduction

Rates of stress and anxiety are alarmingly high in university communities. Even before the COVID pandemic, 40% of college students reported at least mild (though subclinical) anxiety symptoms, which are often tied to stressors such as academic performance, pressure to succeed, post-graduation plans, and financial concerns (Beiter et al., 2015). The COVID-19 pandemic only exacerbated these trends, prompting a rise in high degrees of loneliness, significant depressive symptoms, and moderate to severe anxiety symptoms in young adults (Horigian et al., 2021). Negative mental health symptoms like stress, co-rumination, and low well-being are of pressing concern among university faculty and staff members, who can experience financial difficulties, lack of social support, and burnout (American Council on Education, 2021; Boren, 2014).

Despite a clear need for mental health support, most people in need of mental health services either because they meet the criteria for a diagnosis or feel as though they could use support do not receive treatment, a problem that extends to university populations (Kazdin, 2017). There are multiple reasons why those in need of mental health care might not receive it. First, individuals experiencing mental health concerns (particularly anxiety symptoms) may delay or avoid seeking treatment due to a lack of resources/knowledge, feeling embarrassed about needing help, wanting to handle problems on their own, or stigma (Gaddis et al., 2018; Goetter et al., 2020), along with many logistical barriers (e.g., transportation, costs, insurance; Chartier-Otis et al., 2010). In university settings, mental health services through employee assistance programs are critically under-used by employees, for reasons like fear of problems being disclosed to employers (Wu et al., 2021). Thus, the first group of barriers to accessing care involves factors that lead a person not to seek treatment. Second, among those facing mental health concerns who actually do seek out treatment, individuals may then struggle to access a provider in a timely way. For example, college counseling centers are seeing increases in the number of clients seeking care, often resulting in waitlists (Xiao et al., 2017). Even though not all of those in need of mental health care actively seek it out, there are still not enough providers to treat those requesting services.

Mobile health (mHealth) interventions offer one promising way to address many of the barriers tied to accessibility of mental health services on university campuses. Critically, they can be scaled and delivered much more flexibly than traditional, face-to-face interventions, given they can often be completed in any place at any time, providing more people the opportunity to receive care (Hollis et al., 2015). Though mHealth interventions show clear promise to improve psychological symptoms (Kozlov et al., 2022), we have a limited understanding of which specific elements of mHealth apps are most beneficial to users. Further, we know little about the effect of cognitive mHealth interventions on short time scales (e.g., over the course of minutes) given that most studies examine changes over several weeks.

Additional intervention methods to address the growing need for mental health support include secondary prevention efforts; these interventions are offered to people who are experiencing subclinical symptoms or who are at heightened risk for developing clinical problems to prevent symptoms worsening and reaching a clinical level (Lau & Rapee, 2011; Stockings et al., 2016). There is compelling evidence that clinical threshold anxiety is more likely to occur in groups with subclinical anxiety (Zhong et al., 2024), along with research suggesting that internet- and mobile-delivered secondary prevention methods for anxiety and depression show preliminary promise at reducing symptoms (Schmitt et al., 2022; Zarski et al., 2024).

Accordingly, the present study focuses on the relationship between brief mHealth intervention doses with different characteristics and clinically relevant short-term outcomes based on responses to ecological momentary assessment questions administered immediately post-intervention dose. The application, called Hoos Think Calmly, is a mobile adaptation of a web-based Cognitive Bias Modification for Interpretations (CBM-I) program designed to shift thinking patterns that are common among people experiencing uncomfortable emotions like anxiety at both clinical and subclinical levels.

### The Hoos Think Calmly Application

People experiencing anxiety tend to interpret ambiguous information in rigid, negative ways (called a negative interpretation bias; Blanchette & Richards, 2003) and this bias is a well-studied mechanism that maintains anxiety and mood problems, making it a logical target for intervention. CBM-I aims to encourage more flexible thinking by repeatedly showing participants scenarios that end in positive or benign ways, or that encourage more resiliency-focused interpretations. CBM-I mHealth interventions can reduce stress and anxiety, particularly symptoms of social anxiety disorder (Daniel et al., 2020; Eberle et al., 2024; Hirsch et al., 2018; Larrazabal et al., 2024; Liu et al., 2017), though there are also null or mixed findings (see Fodor et al., 2020; Gober et al., 2021). A meta-analysis concluded that CBM-I for anxiety has some of the strongest effects for any type of CBM across problem areas (Fodor et al., 2020). Even for those with subclinical levels of anxiety, depression, and stress, learning how to manage and regulate distressing feelings is a critical skill. CBM-I is designed to encourage more flexible thinking about emotionally challenging situations, which is a key emotion regulation skill and is very aligned with teaching distress tolerance and other evidence-based emotion regulation skills. Additionally, CBM-I can be scaled more readily than many other cognitive behavioral therapy-related digital interventions that typically perform better with coaches (see reviews by Bernstein et al., 2022, and Karyotaki et al., 2021; along with Haaf et al., 2024), which reduces scalability. CBM-I can also be delivered in small chunks (i.e., microdoses), which are intended to more closely match how people actually use their devices than a more lengthy session model, which is how many digital cognitive-behavioral programs are designed. CBM-I may thus be a promising option for implementation in mHealth interventions like Hoos Think Calmly and “digital apothecaries” that offer various evidence-based treatments (Muñoz et al., 2018).

Hoos Think Calmly is a new mobile application version of MindTrails, a web-based CBM-I program in which participants read descriptions of brief ambiguous situations, and the final word is only a fragment of a word or is missing (modeled off ambiguous scenarios training paradigms; see Mathews & Mackintosh, 2000). Then, participants are prompted to complete the final word, which establishes either a positive/benign or negative emotional outcome to the situation and therefore only fits one valenced interpretation pattern. This is followed by a comprehension question that reinforces the valenced interpretation that was determined by completing the final word in the previous part (examples below). Evidence suggests that positive CBM-I training on MindTrails web is effective in reducing negative interpretation bias and anxiety symptoms (e.g., Ji et al., 2021; Larrazábal et al., 2024; Eberle et al., 2024).

Hoos Think Calmly was created specifically for the University of Virginia (UVA) community members who reached out about wanting help managing feelings of anxiety, depression, or stress, regardless of whether their symptoms were at subclinical or clinical levels. We sought to implement a patient-centered approach that is inclusive, given one of the values of a digital intervention is its scalability, so it is easier to deliver care to the range of people who feel they would benefit from it. It incorporates new features designed to enhance personalization and engagement, such as content tailored to a university community, post-CBM-I emotion regulation and resource recommendations, new scenario formats, a gamified leveling-up structure, and choice of stressor domain to target. These elements were incorporated based on qualitative interviews with members of the UVA community, in which participants mentioned relatability to the content of scenarios, gamification/varying levels of difficulty, and customizability as things they would like to see in the app (Leventhal et al., 2022), and they align well with reported characteristics hypothesized to influence engagement (see review by Perski et al., 2017). Engagement with mHealth interventions is assumed to be crucial to their effectiveness, with greater engagement being positively associated with intervention outcomes (see review by Wei et al., 2020). Also, Hoos Think Calmly is administered in ultra-brief 5–10 min sessions, otherwise known as microdoses, which vary elements of the training material and its delivery to increase personalization and engagement.

### Assessing mHealth-Linked Affective and Cognitive Changes in Real Time

mHealth interventions offer a low- or no-cost, accessible treatment option and can be effective at addressing a wide range of mental health problems, including stress, sleep disturbances, anxiety, depressive symptoms, general mental health, and emotional well-being (Hollis et al., 2015; Kozlov et al., 2022; Kubo et al., 2021; Lee et al., 2018; Pham et al., 2015), though the vast majority of mHealth programs have limited real world testing. For anxiety specifically, the most common types of interventions that mHealth applications apply are cognitive behavioral therapy, problem-solving therapy, and psychoeducation (see review by Leong et al., 2022). In addition, there are some attention training (see review by Zhang et al., 2018) as well as many mindfulness-based applications (Kozlov et al., 2022; Kubo et al., 2021).

Studies on mHealth interventions often measure results on a long-term scale (e.g. six weeks after starting a program). To our knowledge, there has been little research on the immediate effects of mHealth interventions, even though examining short-term change after doses with different app features may provide valuable insight into which components of an mHealth intervention are more (vs. less) helpful for users in the moment and may be important for improving engagement and retention. Accordingly, the present study uses ecological momentary assessment (EMA) as participants complete mHealth microdoses in their daily lives. There is evidence that EMA obtains more accurate responses in self-reported symptoms than standard questionnaire responses, given EMA usually asks about immediate or very recent symptoms and does not rely heavily on retrospective reporting (Grossman et al., 2020). EMA can capture improvements in symptoms such as depression and anxiety (Grossman et al., 2020; Juengst et al., 2015; Shiffman et al., 2008) and self-efficacy for managing negative emotions, showing that EMA can capture real-time changes in emotion regulation on this short timescale (Kleiman et al., 2023).

By measuring short-term change via EMA, we can assess in-the-moment feelings with high accuracy and can test which features of our brief intervention doses (e.g., stressor domain selected for training or writing demands) are linked to short-term changes. Importantly, understanding which intervention feature(s) are associated with affective and cognitive changes at the time a user chooses to engage with the intervention could ultimately aid in developing just-in-time adaptive interventions (JITAIs; Nahum-Shani et al., 2018) that offer accessible, personalized treatments to people when and where they need them. It may also help with engagement and attrition, which are serious challenges for most mHealth programs (see review by Short et al., 2018). Based on the research surrounding habits, we anticipate that increasing the positive momentary effects of interventions measured via EMA will also increase sustained engagement with and adherence to the intervention (Verplanken & Orbell, 2022; Wood & Rünger, 2016). More generally, we think that when people choose to do an intervention in the middle of the day, they are often hoping to feel better in some way in that moment (though this is likely not always the motivation; see response to Reviewer 2, point 10 below). For example, our qualitative research conducted before the parent study suggested that some participants would want to use the program to feel better day-by-day and see this progress plotted throughout the week (Leventhal et al., 2024). Additionally, qualitative feedback interviews after the parent study revealed that some participants appreciated the app’s ability to change their thoughts “in the heat of the moment” (Livermon et al., 2025). In the anxiety literature more broadly, we know that people experiencing anxiety (both clinical and subclinical) tend to have low distress tolerance, and find the experience of distress particularly aversive (French et al., 2022; MacDonald et al., 2014; Mitchell et al., 2013). Research suggests that anxious individuals frequently want to eliminate negative feelings as soon as possible (Sandel-Fernandez et al., 2023), and often use maladaptive emotion regulation strategies to avoid or suppress distress instead of healthier strategies (Jaso et al., 2020; Jeffries et al., 2015). Thus, we expect that using EMA to measure in-the-moment affective and cognitive changes after microdoses will give us insight into whether our app is a helpful tool for managing stress in the moment in an adaptive way.

At the same time, we recognize that what ‘feels good’ in the moment may not be what is most helpful to reduce symptoms in the long term (e.g., avoidance can relieve distress in the moment while ultimately worsening anxiety), so there are many open questions about what short-term impacts to expect or even aim for. Hoos Think Calmly was designed to promote long-term symptom reduction through building a new thinking skill, but understanding short-term impacts is likely critical to secure the ongoing practice needed for the long-term gains, especially for mHealth interventions that do not involve a coach or therapist who is encouraging ongoing participation. Additionally, short-term changes in symptoms, which can be measured via EMA, show preliminary evidence as predictors and mediators of long-term changes in symptoms. For example, daily changes in emotion predict changes in trait anxiety that occur over a year (Mitchell et al., 2024); daily changes in mood predict depression level and suicidal ideation at the end of a few months (Horwitz et al., 2023). Thus, better understanding which features of individual intervention sessions are associated with differential momentary outcomes may also help the field design interventions to more effectively address mental health concerns over time.

Specific to cognitive bias change in the short-term, a few studies have shown that interpretation bias can be altered in single-session interventions (SSIs): one study found that an online CBM-I intervention administered in just five blocks of 10 ambiguous scenarios had a large effect on reducing threat interpretations (Riddleston et al., 2023), and another study found that a 45–50 min computer-based anxiety sensitivity intervention including CBM-I was successful at changing interpretation bias (Capron et al., 2017). However, while these results indicate reduction of negative interpretation bias on relatively shorter time-scales, results for CBM SSIs are mixed (Hallion & Ruscio, 2011) and we have yet to study whether there can be changes in cognitive and affective outcomes over the course of just minutes. Although SSIs are administered in one sitting, these are often interventions similar in length to a full therapy session, including multiple blocks of scenarios. Our intervention was administered in 5–10-min micro-sessions, while participants were going about their daily lives rather than in a controlled laboratory or clinical setting. Thus, EMA is a useful assessment method to determine what app features lead participants to report better or worse outcomes after single sessions. This knowledge can help the field design interventions that will more effectively achieve positive outcomes in the moment, which, in turn, may increase sustained engagement (though testing this latter step is not within the scope of this study).

### Present Study

Hoos Think Calmly is an mHealth application that uses brief CBM-I microdoses with the goal of changing thinking patterns often associated with anxiety and depression. One of the ways we measured this change is through EMA surveys administered before and after each microdose. These microdoses have multiple elements of personalization and content tailoring across various features of training material and delivery. We focused these analyses on stressor domain targeted in training and on writing demands, in part because deciding how to manage these features comes up when designing for many apps, so that adds to generalizability of the current study, and in part because we were testing novel variants of these features in this app.^1^ Specifically, when starting a microdose, participants chose the stressor domain causing them the most stress in the moment from the following options: (1) academics/work/career development, (2) family and home life, (3) finances, (4) mental health, (5) physical health, (6) romantic relationships, (7) social situations, and (8) discrimination. The content of the training scenarios differed across stressor domains. Additionally, the writing demands of the CBM-I scenarios that participants completed in the app differed over time to add variety and increase desirable difficulty. These included: (1) short scenarios with one letter missing from the final word fragment; (2) short scenarios with two letters missing; (3) fill-in-the-blank short scenarios with the entire last word missing; (4) “write your own” short scenarios; and (5) long scenarios. Our predictor variables and outcome variables are further described in the “Variables“ section below.

Our goal is to evaluate the short-term/in-the-moment effects of a personalized mHealth intervention through EMA and to determine which features of a microdose are associated with: (1) the greatest positive momentary affect post-microdose while controlling for affect pre-microdose, (2) the greatest perceived ability to do cognitive reappraisal post-microdose, and (3) the greatest perceived ability to manage emotions post-microdose. These outcomes were selected because they capture both CBM-I target engagement (cognitive reappraisal requires assigning a new meaning or interpretation) and standard user goals for mental health interventions (to feel more positive affect and regulate hard emotions). The hypotheses and analytic plan for this study were pre-registered (https://osf.io/jg4vu) and are described below following a more in-depth description of the feature and formatting variations in the program that were examined as predictors.

## Methods

### Participants

**Fig. 1:**
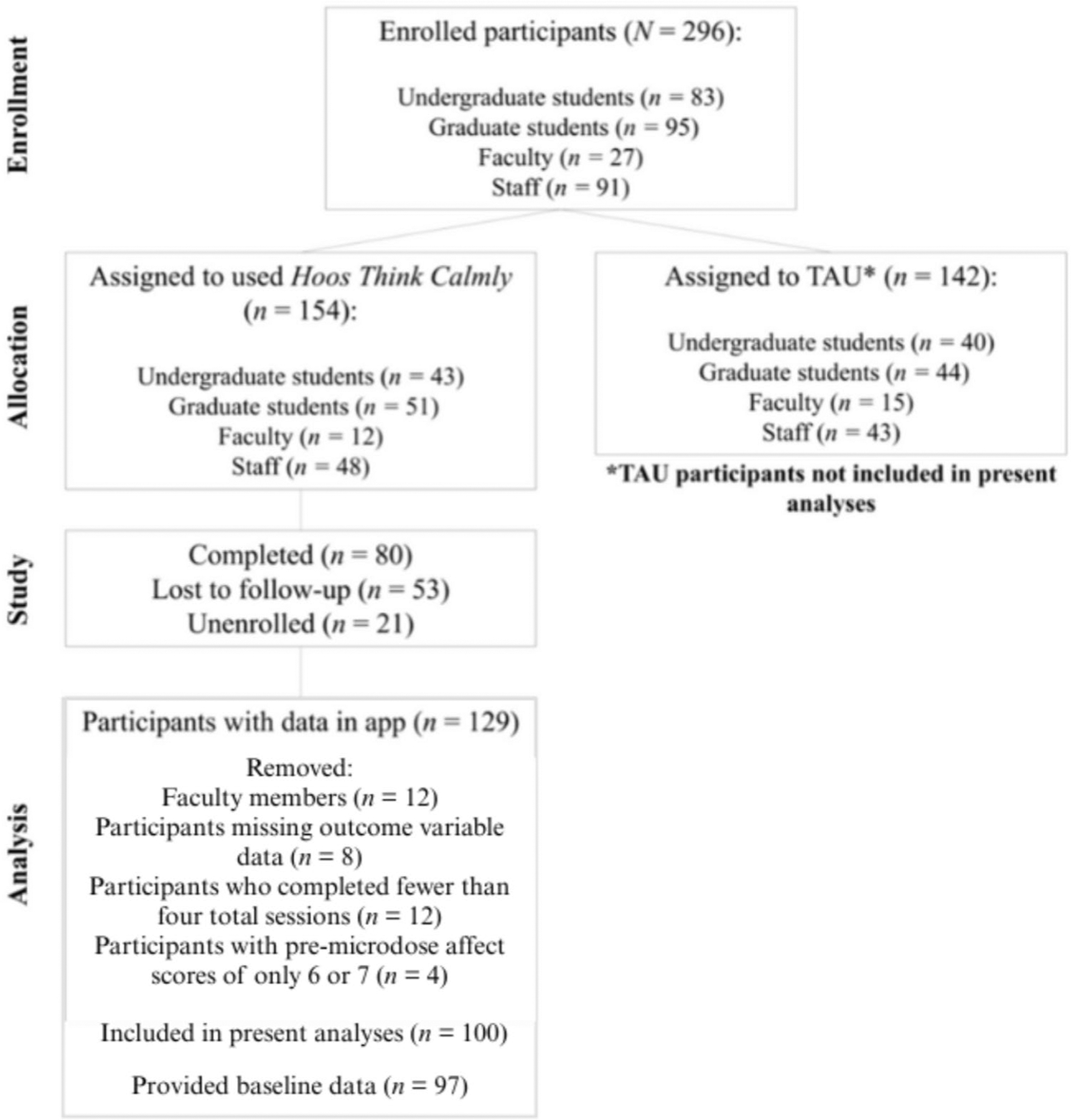
CONSORT Diagram

This study included *N* = 100 participants (See *CONSORT* Diagram; Fig. 1). Data collection began November 2022. For the full parent trial, we recruited *N* = 296 participants, who were undergraduate students (*n* = 83), graduate students (*n* = 95), faculty members (*n* = 27), and staff members (*n* = 91) from the Hoos Think Calmly community. Participants recruited from each of the four Hoos Think Calmly groups were randomly assigned to either use Hoos Think Calmly for a 6-week intervention period (*n* = 43 undergraduate students; *n* = 51 graduate students; *n* = 12 faculty members; *n* = 48 staff members) or to a treatment as usual (TAU) control group, which involved continuing whichever strategies they were currently using to manage distress (*n* = 40 undergraduate students; *n* = 44 graduate students; *n* = 15 faculty members; *n* = 43 staff members). The active study period lasted for six weeks, followed by an additional two-week follow-up assessment. To be eligible to participate, participants must have: (1) been over 18 years old, (2) been a current undergraduate student, graduate student, faculty member or staff member at Hoos Think Calmly, (3) owned a smartphone that meets technological requirements necessary to download and run the mobile application (i.e., iPhone running iOS 10 or later or an Android phone running Android 5.0 or later), and (4) been able to access and use their phone during the day.

To increase accessibility, there were no exclusion criteria related to psychological symptoms, and no clinical severity threshold was required to participate. In our study (based on the 97/100 participants who reported baseline data), 38.14% of participants reported anxiety symptom scores (measured using the Overall Anxiety Severity and Impairment Scale [OASIS; Norman et al., 2006]) of 8 or above, which is commonly considered the threshold indicating a probable anxiety disorder (Campbell-Sills et al., 2009). Additionally, 22.68% of participants reported depression symptom scores (measured using the Patient Health Questionnaire-2 [PHQ-2; Kroenke et al., 2003]) of 3 or above, which is commonly considered the threshold indicating probable major depressive disorder.

Only those participants assigned to use Hoos Think Calmly are included in the analyses for this project as the control group did not complete the EMA portion of the study. Additionally, we decided prior to conducting analyses to exclude the faculty member participants (*n* = 12) from the main analyses because this group had a very small sample size and few microdoses completed (and we were informed about some technical errors with the faculty program). Full faculty member analyses are included in Supplemental Material A but should be considered preliminary given the small sample and some implementation coding errors. Additionally, Supplemental Material C includes two main analyses run with the full sample (undergraduates, graduates, staff, and faculty), which were conducted during the peer-review process as recommended by reviewers.

### General Procedure

Participants were recruited via on-campus health and wellness providers, organizational email lists, and on-campus flyers. Interested participants provided informed consent virtually via DocuSign. The study lasted eight weeks (an active intervention period of six weeks and a two-week follow-up period). During the active intervention period, participants completed up to two microdoses per day and daily surveys in the app. Participants could have completed a microdose at a particular time via three ways: random prompt, specific time requested in the nightly survey completed the previous night, or initiated at any moment manually by the participant. Throughout the active intervention and follow-up period, participants completed mental health and app experience questionnaires every two weeks.

**Fig. 2:**
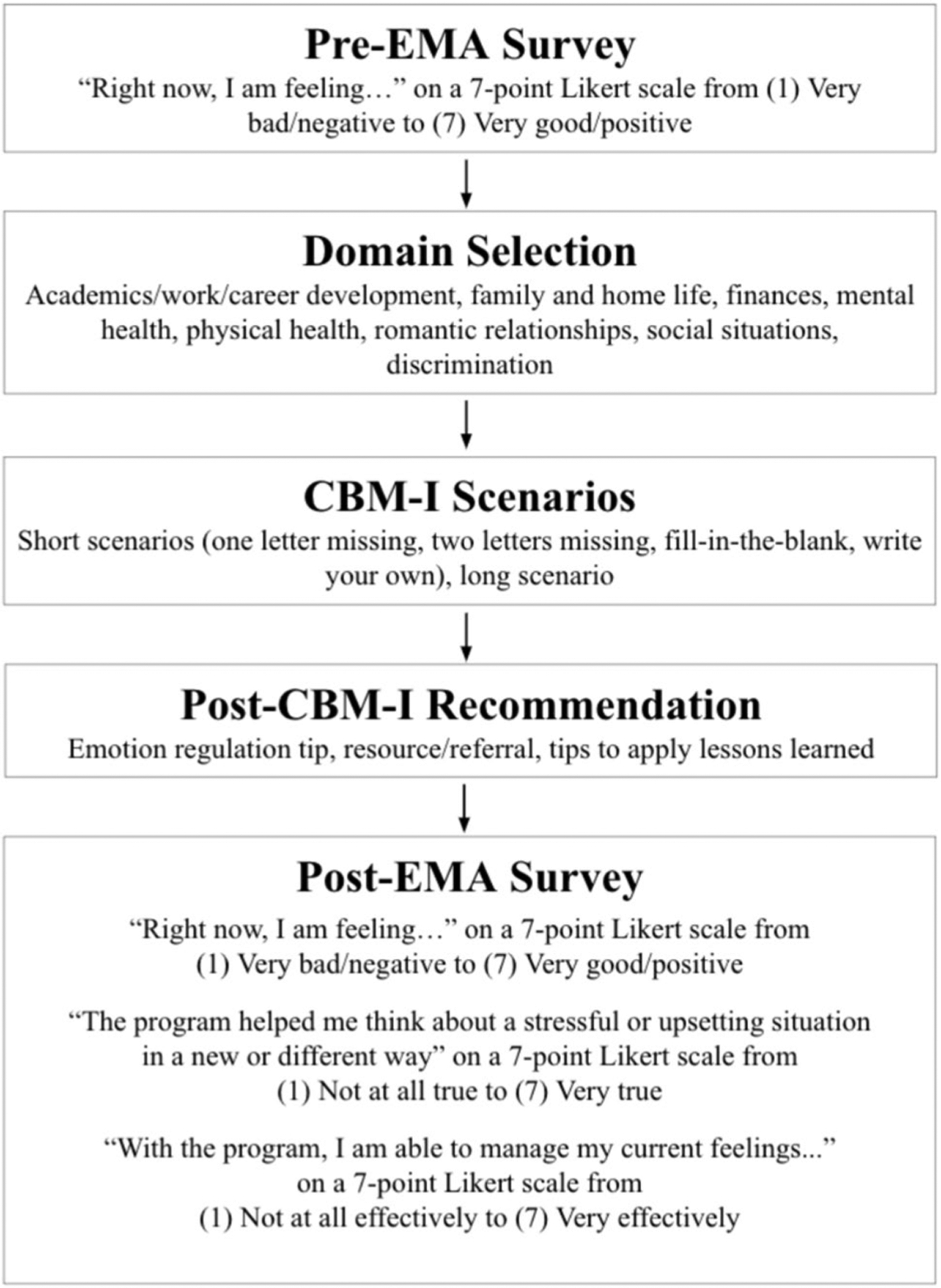
Microdose flowchart

Each microdose consisted of: (1) a brief pre-CBM-I assessment; (2) a CBM-I dose; (3) a post-CBM-I emotion regulation tip, resource/referral, or strategy to apply the CBM-I learning to daily life; and (4) a brief post-CBM-I assessment, as described below. See Fig. 2 for a flowchart of the microdose steps. The content in each microdose involved multiple levels of personalization, which are described in the “Variables“ section.

#### Brief Pre-intervention Assessment

When starting a microdose, participants first completed a brief assessment. They were asked to rate their in-the-moment mood and select the stressor domain that was currently causing them the most stress (e.g., finances, health, relationships). If participants responded that they were feeling positively, we provided them with an *anxious imagery prime*, which prompted participants to choose, describe, and vividly imagine an upcoming situation that they expected would make them feel anxious or worried, and then answer a few questions about how anxious they felt, how vividly they imagined the situation, and how they expected the situation to turn out. This was included to activate the anxious thinking patterns that the training was designed to target. All participants also practiced this priming task during their first training session in the app.

#### CBM-I Dose

Participants were presented with a brief dose of CBM-I tailored to the stressor domain they selected in the pre-intervention assessment. Within the Hoos Think Calmly program, there were three different types of CBM-I scenarios: (1) short scenarios; (2) write-your-own scenarios; and (3) long scenarios.

#### Short Scenarios

Were brief stories that consisted of approximately three sentences of content: an initial sentence that provides contextual information, a second sentence that raises uncertainty or ambiguity tied to the emotional threat (e.g., fear of negative evaluation for a social situation), and a third sentence that resolves the ambiguity when the final word is completed. During a microdose, participants were presented with 10 short scenarios, of which there are three types: (1) positive; (2) negative; and (3) resilience. In the *positive* scenarios, which accounted for 70% of the short scenarios, the final sentence resolves the ambiguity in a positive or non-threatening way. In the *negative* scenarios, which accounted for 10% of the short scenarios, the final sentence resolves the ambiguity in a negative or threatening way. The *resilience* scenarios, which accounted for 20% of the short scenarios, initially set up a negative experience but conclude with an additional sentence or two in which the negative resolution is reinterpreted in a way that indicates resilience. We chose to have these variations in valence so that participants can practice *flexible* thinking, not just *positive* thinking; given that not all situations end positively, this method of training is intended to help participants take multiple perspectives and learn that they can recover from hard things. We included more positive scenarios because people with anxiety tend to have rigid *negative* thoughts, so we wanted to reduce that catastrophic thinking pattern. Consider the following example of a CBM-I scenario in the Social Situations stressor domain for undergraduate students that has been adapted to illustrate each of the short scenario formats:

***Positive*:** Your professor asks your class to give presentations for your final project. You have been practicing for several weeks, but you’re not sure how it will go on the big day. While giving the presentation, you feel

[confident].

***Negative:*** Your professor asks your class to give presentations for your final project. You have been practicing for several weeks, but you’re not sure how it will go on the big day. While giving the presentation, you make a big

[mistake].

***Resilience***:

***Part 1:*** Your professor asks your class to give presentations for your final project. You have been practicing for several weeks, but you’re not sure how it will go on the big day. While giving the presentation, you make a big

[mistake].

***Part 2:*** After the presentation, you realize that these mistakes are common among students. The professor probably thinks your recovery and ability to continue presenting was very

[admirable].

Each of the short scenario microdoses involved completing the last word of the sentence (i.e., “confident”, “mistake”, or “admirable” in the examples above). For some microdoses, the final word was missing one letter that participants must fill in; for other microdoses, the final word was missing two letters; for others, the final word was completely missing and participants had to fill in the blank. The “missing letter” microdoses included positive, negative, and resilience scenarios; the “fill-in-the-blank” microdoses prompted participants to choose positive final words. Additionally, the “missing letter” microdoses were followed by a comprehension question (e.g., “Is the professor disappointed with your presentation?” [Yes/No]) that was designed to reinforce the resolution of the emotional ambiguity, because the question has only a single correct answer once the scenario is completed by filling in the missing letter(s). The “fill-in-the-blank” microdoses did not include comprehension questions because participants generate different final words and a generic comprehension question would not make sense with all possible participant responses.

For the **write-your-own scenarios**, participants were provided instructions to think about an anxiety-provoking situation in their past or future, and then generate their own CBM-I story based on this situation. Participants were encouraged to provide a positive or non-threatening ending to the story they write. Finally, they were asked to list potential reasons why the non-threatening ending could be likely to occur to them (e.g., reasons why they think they could tolerate the situation, or evidence that something terrible is unlikely to happen). This type of scenario was included to encourage application of the skills learned to personally relevant situations. During a write-your-own scenario microdose, participants completed only one write-your-own scenario given that this task requires more cognitive effort and time than the short scenarios.

Finally, the **long scenario** microdoses were similar to the short scenarios in that they also consist of around three sentences of scenario content, but they were followed by a variety of thoughts, feelings, or behaviors someone might have in the situation that are either positive, negative, or resilient. After reading a short story, participants were presented with a list of thoughts one might have in that situation, then encouraged to write down their own ideas for potentially helpful thoughts. Participants then followed the same procedure for feelings and for behaviors. This type of scenario was included to encourage flexibility in thinking by making explicit the many different ways a situation might unfold. Given the length of this format, only one long scenario was included per microdose.

To enhance engagement (by adding variety and increasing difficulty), the CBM-I doses followed a “leveling up” structure within each stressor domain over the course of the active intervention period. Specifically, participants completed three microdoses of short scenarios with one letter missing, then advanced to completing three microdoses of short scenarios with two letters missing, then advanced to completing three microdoses of “fill-in-the-blank” short scenarios, and finally advanced to completing three microdoses of “write your own” scenarios. This sequence then repeated for subsequent microdoses. Additionally, every sixth microdose completed within a stressor domain was a long scenario.

#### Post-CBM-I Recommendation.

Following each CBM-I dose, participants were presented with one of three post-CBM-I recommendations: (1) emotion regulation tip; (2) resource/referral; or (3) tip to apply lessons learned from the microdose to their daily life.

#### Brief Post-Intervention Assessment.

At the end of each microdose, participants were given another brief assessment similar to the one administered before the microdose. This survey again asked about the participant’s in-the-moment affect, then asked if the program helped them think about a stressful situation in a new way and manage their current feelings.

### Variables

#### Predictor Variables: Microdose Characteristics

##### Stressor Domain

When starting a microdose, participants chose the stressor domain causing them the most stress in the moment; the domain selected could change with each microdose. Stressor domains were preselected in collaboration with UVA community members through qualitative interviews during a user-centered design phase (Leventhal et al., 2024). From these interviews, we set eight broad areas that interview participants said trigger anxiety: (1) academics/work/career development, (2) family and home life, (3) finances, (4) mental health, (5) physical health, (6) romantic relationships, (7) social situations, and (8) discrimination. The content of the CBM-I scenarios, as well as the content of the emotion regulation tips and resources/referrals, differed across stressor domains. For example, an academics/work/career development CBM-I scenario might focus on preparing for a final exam, whereas a finances scenario might focus on anxiety tied to budgeting. Similarly, an emotion regulation tip for academics/work/career development might emphasize breaking work down into smaller pieces, whereas an emotion regulation tip for finances might encourage participants to come up with enjoyable, low-cost activities. The tips to apply lessons learned in daily life were more general and did not differ across stressor domains.

The structure of the discrimination stressor domain microdose was unique. If a participant selected the discrimination stressor domain during the pre-microdose survey, they were first presented a statement validating their feelings and offering a selection of tools and resources. Participants were then able to select what they would like to do next from a set of options, which included journaling, receiving resources tied to reporting an instance of discrimination, receiving resources for self-care and wellness following an instance of discrimination, and receiving resources tied to community-building and social support, or participants could choose not to receive any additional tools at that time. This structure was determined based on qualitative interviews with diverse community members and leaders of cultural groups at UVA. Specifically, we received feedback that CBM-I exercises might not be suitable for handling discrimination-related anxiety, in part because these instances are usually very personal and people did not want a proscriptive, one-size-fits-all response, and in part because we wanted to avoid inadvertently ‘gaslighting’ participants by suggesting their thinking about an experience of discrimination was not accurate or valid in some way (Leventhal et al., 2024).

##### Writing Demand

As detailed above, there were multiple types of CBM-I scenarios that participants completed in the app, with varying amounts of generative writing: (1) short scenarios with one letter missing; (2) short scenarios with two letters missing; (3) fill-in-the-blank short scenarios with the entire last word missing; (4) “write your own” short scenarios; and (5) long scenarios.

#### Outcome Variables: Ecological Momentary Assessments (EMA)

##### Momentary Affect Post-Microdose

Immediately before and after each microdose, participants reported on their current affect via a brief EMA survey. Specifically, participants responded to the prompt, “Right now, I am feeling…” on a 7-point scale from (1) Very bad/negative to (7) Very good/positive. Participants were asked about their current feelings very broadly: “right now I am feeling bad/negative vs. good/positive”. This choice to assess core affect (i.e., feeling good/bad; Russell, 2003) instead of several discrete emotions was made to keep the assessments brief with the goal of reducing user burden.

After preregistering our analysis plans, we realized the challenge of using survey data for pre-microdose affect scores of 6 or 7, because these initially-positive affect scores triggered participants to receive the anxiety imagery prime described above. The anxious imagery prime exercise likely altered these positive affect scores given the imagery was intended to activate anxious thinking, but we did not assess affect again post-anxious imagery prime (but before the CBM-I training). Thus, pre-microdose affect scores of 6 and 7 do not actually accurately reflect the participants’ affect *going into the CBM-I training*, but rather just *before receiving the anxious imagery prime*. As a result, we do not have a valid pre-training baseline affect score to use to examine change following training. With this in mind, we decided to make a change to our preregistered plans and remove all microdoses with pre-microdose affect scores of 6 or 7 for primary analyses, only using microdoses with pre-microdose affect scores between 1 and 5. See Supplemental Material A for analyses with all pre-microdose affect scores 1–7.

##### Reappraisal Efficacy

Following each microdose, participants reported the extent to which the program helped them think more flexibly (i.e., engage in cognitive reappraisal) via a brief EMA survey. Participants responded to the prompt, “The program helped me think about a stressful or upsetting situation in a new or different way” on a 7-point scale from (1) Not at all true to (7) Very true.

##### Emotion Regulation Efficacy

After each microdose, participants reported the extent to which the program helped them manage their emotions via a brief EMA survey. Participants responded to the prompt, “With the program, I am able to manage my current feelings…” on a 7-point scale from (1) Not at all effectively to (7) Very effectively.

Of note, we made the decision to treat our outcome variables as continuous (vs. ordinal). See the preregistration for justification (https://osf.io/jg4vu).

### Hypotheses

The short-term emotional effects of CBM-I are difficult to predict in advance. On one hand, CBM-I urges participants to imagine themselves in stressful situations and challenge their automatic interpretations of ambiguity, which we would not expect to be a particularly enjoyable experience. Conversely, CBM-I works to reduce negative interpretations and increase positive interpretations (Ji et al., 2021), which may induce a positive emotional state. Thus, for multiple outcomes, we lay out competing hypotheses or conduct exploratory analyses.

#### Hypotheses Set 1—Stressor Domain

Social anxiety is often a target of CBM-I interventions. Much of the existing literature on CBM-I has found significant positive effects of CBM-I on social anxiety symptoms (see review by Mobini et al., 2013, though there have also been mixed and null findings: Fodor et al., 2020), and many studies have found CBM-I effective at reducing social threat interpretation bias (e.g., Daniel et al., 2020; Riddleston et al., 2023). However, little to no research has been done specifically on the other stressor domains included in Hoos Think Calmly. Thus, we do not know if the target stressor domain selected for training will predict different short-term outcomes, so these tests are exploratory. Specifically, we explore whether there is a significant difference in momentary affect (**Exploratory Hypothesis 1a**), emotion regulation efficacy (**Exploratory Hypothesis 1b**), and reappraisal efficacy (**Exploratory Hypothesis 1c**) post-microdose based on the stressor domain selected.

We also report outcomes *within* each stressor domain to better understand the intervention’s immediate effects (i.e., beyond knowing whether affect differentially changed following training in one stressor domain vs. another, we also want to understand the change pattern when doing training in each domain).

Importantly, participants may vary in the number of times they choose each stressor domain, resulting in participants having more practice in some content stressor domains than others. Because of this, it should be kept in mind that practice may account for some of the effects of stressor domain.

#### Hypotheses Set 2—Writing Demand

There are mixed findings on the effects of more cognitively demanding intervention material on participants. Some studies suggest that more demanding responses (e.g., filling in 2 vs. only 1 letter to resolve a word fragment) yield more positive results/a decrease in negative symptoms, perhaps because the exercise is more engaging (Steinman et al., 2021). However, other studies suggest that participants are more likely to drop out of interventions when a written response is requested, suggesting less engagement with the intervention and potentially less benefit from it (Dobias et al., 2022).

In our study, scenarios requiring more writing were less structured, so participants could create responses more closely related to situations in their own lives. Studies show that information perceived to be more personally relevant enhances motivation for changing behavior (see review by Lustria et al., 2009); this suggests that participants’ ability to write personal examples in unstructured write-in scenarios may lead to more positive outcomes. Relatedly, the long scenarios explicitly required participants to read a list of thoughts, and then generate their own thoughts in response to a given scenario, and then repeat this sequence for feelings and behaviors. Additionally, the write your own scenarios required participants to list reasons why a scenario might end in a non-threatening way. Both of these exercises are intended to enhance cognitive flexibility by challenging participants to think about situations in multiple ways. This more flexible thinking practice was expected to reduce distress and increase reappraisal and emotion regulation efficacy.

However, more cognitively demanding work may not be immediately enjoyable for participants, especially because we are asking participants to imagine themselves in stressful situations. The writing-heavy scenario formats may feel more difficult for participants because they are likely trying to challenge *their own* rigid, negative thinking about personally significant stressors, and so may feel less efficacious than when trying to think about the preset, likely less personally significant stressors presented in the short missing letter(s) scenarios, which had the lowest writing demands. Thus, we have competing hypotheses regarding the effects of writing demand:

##### Competing Hypothesis 2a

Less writing will be associated with significantly better short-term outcomes, such that momentary affect post-microdose, reappraisal efficacy, and emotion regulation efficacy will be greatest/most positive following microdoses containing scenarios that do not require any writing (one-letter missing, two-letters missing), followed by scenarios that require write-ins but are structured and require less independent generation of written content (fill-in-the-blank), followed by write-in options that are unstructured and require more extensive generation of written content (write your own, long scenarios).

##### Competing Hypothesis 2b

More writing will be associated with significantly better short-term outcomes, such that momentary affect post-microdose, reappraisal efficacy, and emotion regulation efficacy will be greatest/most positive following microdoses containing write-in options that are unstructured and require more extensive generation of written content (write your own, long scenarios), followed by scenarios that require write-ins but are structured and require less independent generation of written content (fill-in-the-blank), followed by scenarios that do not require any writing (one-letter missing, two-letters missing).

We also report outcomes *within* each writing exercise to better understand the intervention’s immediate effects (i.e., beyond knowing whether affect differentially changed for one writing demand level vs. another, we also want to understand the change pattern within each different level of writing demand).

Importantly, the microdoses containing write-in scenarios appeared later in the program (i.e., within each stressor domain targeted in training, participants must have completed three microdoses consisting of scenarios with one letter missing and three microdoses consisting of scenarios with two letters missing before they reached any scenarios involving writing), so participants got more practice with non-write-in microdose types and were further along in the program by the time they reached writing-intensive microdoses. Because of this, we acknowledge that practice and sequence are confounded with writing demand.

### Statistical Analyses

In line with our preregistered analytic plan (https://osf.io/jg4vu), we ran a set of separate linear mixed effects models predicting momentary affect post-microdose and self-report of reappraisal and emotion regulation efficacy from our categorical variables: stressor domain targeted in training and writing demand (and post-CBM-I recommendation as outlined in Supplemental Material A). A description of prior analyses with this dataset is also included in the preregistration.

Analyses were conducted in R Version 4.2.3 using the lme4 package (Bates et al., 2015). We interpreted *p*-values using an alpha level of 0.05. Each of the predictor variables was examined in separate tests for each outcome variable, described below. Prior to running these tests, we first built three separate baseline models (one for each outcome variable) that partitions the variance in outcome (i.e., post-microdose affect, reappraisal efficacy, emotion regulation efficacy) and allows for fixed and random effects for outcome variable intercept, which allowed us to examine the average of each outcome across the full sample (regardless of microdose characteristics), as well as the percentage of the variance in each outcome variable that can be explained by individual differences.

#### Tests 1–3 (H1a-H1c)—Stressor Domain

After building our baseline models for each outcome variable (i.e., post-microdose affect, reappraisal efficacy, emotion regulation efficacy), stressor domain was added into the random intercepts models as a categorical predictor, and the *emmeans* package was used to explore all pairwise differences with a Holm correction.

#### Tests 4–6 (H2a and H2b)—Writing Demand

After building our baseline models for each outcome variable (i.e., post-microdose affect, reappraisal efficacy, emotion regulation efficacy), writing demand was added into the random intercepts models as a categorical predictor, and the *emmeans* package was used along with custom contrast codes to compare the difference in our outcome variables for the write-in scenarios versus fill in the blank scenarios versus the non-write-in scenarios. We grouped together long scenarios with write your own scenarios in a “large amount of writing” group, and one-letter missing scenarios with two-letters missing scenarios in a “no writing” group, rather than looking at writing demand at each level, to better test our hypotheses (as we are interested in looking at the effects of different amounts of writing, and these scenario types have similar writing requirements; see Appendix for specific contrasts to be tested).

For all tests examining post-microdose affect, we also included pre-microdose affect score in the random intercepts model as a predictor. By regressing the post-microdose affect score onto the pre-microdose affect score, we use residualized change scores to account for differences in pre-microdose affect score. For all tests examining reappraisal and emotion regulation efficacy, if there was an association between the predictor variable (stressor domain, writing demand) and the reappraisal or emotion regulation efficacy outcomes, we then added pre-microdose affect score into the random intercepts model as a predictor. By adding in pre-microdose affect score as a covariate, we removed any effect of pre-microdose affect on the relationship between the variables—if the relationship changed, that would mean that pre-microdose affect was different across stressor domains or writing demands and therefore must be considered in the model as another factor affecting reappraisal or emotion regulation efficacy.

At each stage in the tests, we compared the more complex model to the less complex model that immediately preceded it, using the AIC and BIC to determine which model better fit the data.

Additionally, for testing affect change within each stressor domain and writing demand level, we similarly created linear mixed effects models with Score (1–5) as the outcome variable and Time (pre- or post-microdose) as the predictor, still with individual participant as part of the model to account for the percentage of the variance in each outcome variable that can be explained by individual differences, for each domain and writing demand separately. We then used ANOVAs to determine the *F*-values and *p*-values for these models.

In our preregistered analyses, we originally planned to also run random slopes and intercepts models, but learned that these models would overparameterize the data and accordingly did not run them.

## Results

### Sample

154 participants were randomized to use the Hoos Think Calmly application in the parent study. Of these, we received microdose data from 129 participants (meaning they completed at least one microdose in the app), nine of whom were faculty members and thus removed (out of 12 faculty randomized to the condition, meaning three did not complete a single microdose). From the remaining 120 participants, we removed any microdoses that were missing values of our EMA outcome variables, which removed eight participants whose microdoses all had missing data. Then, we removed 12 participants who completed fewer than four microdoses total throughout the study; we believe that these participants are not providing reliable data on the effects of the intervention considering that they completed less than 5% of the recommended sessions. Last, from these 104 participants, we removed any microdoses with pre-microdose EMA scores of 6 or 7, which removed 4 participants whose pre-microdose affect was always 6 or 7. Thus, our final sample size for analyses was *N* = 100.

### Demographics

93 participants (out of 100 included in our sample) provided demographic information. Most participants identified as White/European (69.89%), non-Hispanic (95.70%), women (84.95%). Participant ages ranged from 19 to 62 (*M* = 31.12, *SD* = 11.23). See Table 1 for full demographic information.

### Microdose Characteristics

Across the full sample, participants completed 1,661 microdoses in total. Microdoses missing either pre- or post-microdose affect, reappraisal efficacy, or emotion regulation efficacy scores (as a result of either participants choosing to skip the question in the app or technical issues in the data being recorded) were removed (*n* = 24), leaving 1,637 microdoses completed across 100 participants. The total number of microdoses completed by participants during their study participation ranged from one (4.8% of total expected) to 62 (over 100% of total expected) microdoses (*M* = 16.37, *SD* = 14.19). Due to a technical error with how the mobile application collected data at the beginning of the study (October 2022—January 2023), writing demand and recommendation data are missing for a portion of the microdoses. Specifically, the microdose stressor domain is known for all but one microdose (99.94%), but writing demand is known for 1,162 microdoses (70.98%). On average, after removing survey responses for pre-microdose affect scores of 6 or 7, participants rated their affect as being somewhat positive both immediately prior to (4.26 out of 5) and immediately after (4.50 out of 5) completing a microdose; participants rated their reappraisal efficacy (4.28 out of 7) and their emotion regulation efficacy (4.52 out of 7) as being somewhat better immediately after completing a microdose. See Table 2 for full descriptives.

For each model, the total variance explained by the fixed effects and the total variance explained by both fixed and random effects, as well as the intraclass correlation coefficient (ICC), is reported in Supplemental Material B. Additionally, all null and random intercepts model results are reported in Supplemental Material B.

See Table 3 for a conceptual summary of the results.

### How Did Post-microdose Affect Scores (Controlling for Pre-microdose Affect) Differ Based on Domain? (H1a)

Post-microdose affect scores were significantly more positive for the academics/work/career development, family and home life, finances, mental health, physical health, and social situations stressor domains compared to the romantic relationships domain, and scores were significantly more positive for academics/work/career development than the discrimination domain; see Table 4 for pairwise comparisons. No other pairwise comparisons across domains were significant. The random intercepts model with domain as a predictor performed significantly better than the null model with no predictor (Chi-square = 60.48, df = 7, *P* < 0.001). See Table 5 for full model comparison, including Akaike Information Criterion (AIC) values.

### How Did Post-Microdose Emotion Regulation Efficacy Differ Based on Domain? (H1b)

Emotion regulation efficacy scores were significantly higher following microdoses completed in the academics/work/career development, family and home life, finances, mental health, physical health, and social situations stressor domains compared to the discrimination domain, and were significantly higher following microdoses completed in the academics/work/career development, mental health, physical health, and social situations stressor domains compared to romantic relationships; see Table 6 for pairwise comparisons. No other pairwise comparisons across domains were significant. The random intercepts model with domain as a predictor performed significantly better than the null model with no predictor (Chi-square = 50.46, df = 35, *P* < 0.001). Additionally, we ran a random intercepts model with pre-microdose affect score as a predictor along with domain to control for the effect of affect on emotion regulation efficacy. Results showed the same significant pairwise comparisons, suggesting pre-session affect level is not affecting the relationship between domain and emotion regulation efficacy. See Table 7 for full model comparisons.

### How Did Post-Microdose Reappraisal Efficacy Differ Based on Domain? (H1c)

Reappraisal scores were significantly higher after microdoses completed in the academics/work/career development, family and home life, finances, mental health, physical health, and social situations stressor domains compared to the romantic relationships and discrimination domains; see Table 8 pairwise comparisons. No other pairwise comparisons across domains were significant. The random intercepts model with domain as a predictor performed significantly better than the null model with no predictor (Chi-square = 83.532, df = 7, *P* < 0.001). Additionally, we ran a random intercepts model with pre-microdose affect score as a predictor along with domain to control for the effect of affect on reappraisal. Results showed the same significant pairwise comparisons. See Table 9 for full model comparisons.

### How Did Post-Microdose Affect Scores (Controlling for Pre-microdose Affect) Differ Based on Writing Demand? (H2a and H2b)

Writing demand data was collected (and thus analyzed) for 81 (out of 100) participants, including 1,162 (out of 1,637) microdoses.

Both scenarios with no writing (one- and two-letters missing; *B* = 0.31, *SE* = 0.10, *P* = 0.002) and some writing (fill-in-the-blank scenarios; *B* = 0.28, *SE* = 0.13, *P* = 0.03) were associated with significantly more positive post-microdose affect than scenarios containing a large amount of writing (write your own and long scenarios). There was no significant difference between post-microdose affect scores for scenarios with no writing (one- and two-letters missing) and fill-in-the-blank scenarios (*B* = 0.03, *SE* = 0.10, *P* = 0.76). See Table 10 for pairwise comparisons. The random intercepts model with writing demand as a predictor performed significantly better than the null model with no predictor (Chi-square = 14.30, df = 4, *P* = 0.006). See Table 11 for full model comparison.

### How Did Post-Microdose Emotion Regulation Efficacy Differ Based on Writing Demand? (H2a and H2b)

There was no significant difference in emotion regulation efficacy scores across writing demands. See Table 12 for pairwise comparisons. The random intercepts model with writing demands as a predictor did not perform significantly better than the null model with no predictor (Chi-square = 9.12, df = 4, *P* = 0.06). See Table 13 for full model comparison.

### How Did Post-Microdose Reappraisal Efficacy Differ Based on Writing Demand? (H2a and H2b)

There was no significant difference in reappraisal efficacy scores across writing demands. See Table 14 for pairwise comparisons. The random intercepts model with writing demands as a predictor did not perform significantly better than the null model with no predictor (Chi-square = 6.85, df = 4, *P* = 0.14). See Table 15 for full model comparison.

### Differences in Pre- to Post-Microdose Affect Scores Within Domains and Writing Demands

There are significant positive changes in affect from pre- to post-microdose within the academics/work/career development, family and home life, finances, mental health, physical health, and social situations stressor domains. However, there is no significant difference in pre- to post-microdose affect scores for the discrimination and romantic relationships stressor domains. Additionally, there are significant positive changes in affect from pre- to post-microdose within the one-letter missing, two-letters missing, and fill-in-the-blank scenarios; there is no significant difference in pre- to post-microdose affect scores for the write your own and long scenarios. See Table 16 for pre- and post-microdose affect score means, as well as *p*-values for change in pre- to post-microdose affect for each model.

### Results Summaries by Hypothesis

Hypotheses 1a, 1b, and 1c were all exploratory hypotheses about the relationship between emotion regulation efficacy, reappraisal efficacy, and post-microdose affect and stressor domain selected. Results showed that all three outcome variables were significantly more positive following microdoses in the academics/work/career development, family and home life, finances, mental health, physical health, and social situations domains than in the romantic relationships or discrimination domains. Hypotheses 2a and 2b, competing hypotheses about the effects of writing demand on post-microdose affect, emotion regulation efficacy, and reappraisal efficacy had mixed support: while microdoses requiring little to no writing (one- and two-letters missing, fill-in-the-blank scenarios) were associated were more positive post-microdose affect than microdoses requiring more writing (write your own and long scenarios), there were no differences in emotion regulation or reappraisal efficacy across writing demands.

## Discussion

The present study examined whether different characteristics of a mobile anxiety intervention were associated with more positive changes in self-reported momentary affect, reappraisal efficacy, and emotion regulation efficacy immediately following a brief session of CBM-I. Specifically, we investigated whether microdose domain, writing demand, or post-CBM-I recommendation type was associated with short-term affective and cognitive changes through EMA surveys administered immediately before and after a microdose on Hoos Think Calmly. Regarding our exploratory hypotheses, stressor domain was associated with differential post-microdose affect, emotion regulation efficacy, and reappraisal scores. As hypothesized (in one competing hypothesis), training sessions requiring less (versus more) writing were associated with more positive post-microdose affect, but, contrary to hypotheses, not differential perceived reappraisal or emotion regulation efficacy. Additionally, positive change in affect scores occurred from pre- to post-microdose in six out of eight stressor domains and three out of five writing demand levels, suggesting that Hoos Think Calmly can provide reliable positive change in affect across just a few minutes for most of our microdose types. After completing a training microdose, participants rated that the program both helped them think about a stressful situation in a new way (reappraisal efficacy) and manage their current emotions (emotion regulation efficacy). This was endorsed, on average, as “somewhat” to “mostly true” on the response scale.

Results for our faculty member group are reported in Supplemental Material A, but we would like to highlight that faculty did not show the significant differences in post-microdose affect, emotion regulation efficacy, or reappraisal efficacy across stressor domains, or the significant differences in post-microdose affect across writing demands, that we saw in our main sample. However, these results were based on a faculty analysis sample size of only six participants (*N* = 12 total, *n* = 9 completed at least one microdose, *n* = 6 remaining after taking the steps outlined in the “Sample“ section above). We also reran two primary analyses: 1) the relationship between post-microdose affect and stressor domain (H1a), and 2) the relationship between post-microdose affect and writing demand (H2a-2b) on the full sample including undergraduates, graduate students, staff members, as well as the faculty, for a total sample of *N* = 106. Supplemental Material C includes these results, which were consistent with our results reported in this main text.

### Stressor Domains Differentially Predict State Outcomes

Our findings indicate that Hoos Think Calmly is better able to address in-the-moment anxiety related to academics/work/career development, family and home life, finances, mental health, physical health, and social situations than anxiety about romantic relationships and discrimination, as these were associated with less positive state outcomes and no significant differences in pre- to post-microdose affect. Notably, pre-microdose affect scores were not significantly lower for these two domains in comparison to the others (See Table 17 for means and standards deviations of pre-microdose affect across domains), indicating that there is not a difference in how participants are feeling coming into a session for these two domains compared to all others, but are leaving feeling no more or less positively, raising interesting questions about why these domains were associated with a less reliable affective response.

CBM-I scenarios in the romantic relationships domain covered a large variety of possible situations (e.g., finding a peer attractive and wondering if the feelings were reciprocated, scheduling or attending a date, dealing with a breakup, or confronting thoughts about being single, among many others). We included a large variety so that scenarios would be relevant to our diverse sample, and to encourage practice of flexible thinking across a variety of situations, which in turn may promote more application of these skills outside of training. Although we view this variety as a strength, it may have come at the cost of the scenarios not all being relevant for particular participants (who may be in any stage of a relationship, or not in one at all), resulting in the attenuated positive affect change. Additionally, our qualitative work indicated that participants find romantic stressors to be a particularly difficult and sensitive area of stress, as romantic relationships are very intimate. As such, this may just be a more difficult stressor to target with this microdose model.

Our finding that affect, reappraisal efficacy, and emotion regulation efficacy were lower for our discrimination domain (vs. other domains) is not altogether surprising. The discrimination domain does not offer CBM-I training; instead, there is an option to journal, as well as a variety of resources tied to reporting an act of discrimination, finding community, allyship, and other categories related to discrimination. Because we are providing participants with resources that they are unlikely to use in that exact moment during the microdose, it is unsurprising to see less positive affect, reappraisal efficacy, and emotion regulation efficacy. Further, experiencing an act of discrimination can be particularly upsetting and is associated with a subsequent increase in depression and anxiety symptoms (Cobb et al., 2021; Livingston et al., 2020), suggesting that, like romantic relationships, this may be a difficult stressor to target. Also, in retrospect we recognized that our resources were particularly geared toward marginalization experiences tied to race and ethnicity or being LGBTQ +, and our post-trial qualitative work with past users (Livermon et al., under review) indicated that the discrimination experiences were not always tied to those aspects of users’ identities. Further co-design with potential users from a broad range of marginalized groups on how to provide effective in-the-moment, likely personalized supports will be important to improve this aspect of the program. For future iterations of the Hoos Think Calmly app, we could further personalize the experience by tailoring scenarios to different demographics, identities and life experiences. Recent advances in artificial intelligence could allow for large advances in personalization potential. Additionally, these results are consistent with other studies that evaluated cognitive techniques to reduce anxiety related to experiences of discrimination (Soto et al., 2025). Collectively, these findings highlight the need to develop more reliable ways to support individuals to manage anxiety that arises in response to these painful experiences.

### More Writing-Intensive Scenarios Predicted Lower Positive Affect

Our results suggest that people tend to feel more positively after a session where they fill in words with letters using tiles in comparison to writing in responses; specifically, there is no difference in affect from pre- to post-microdose for sessions involving writing in multiple-word responses while users reported more positive affect following the one-letter missing, two-letters missing, and fill-in-the-blank scenarios. One potential explanation is that write-in responses require an undesirable level of cognitive effort for participants, either because they do not want to put in the extra work of typing in a response rather than clicking a button, or because they do not want to take the time to think about longer responses; some prior CBM-I research suggests a certain level of desirable difficulty, with even three letters missing being significantly different from zero, one, or two (Steinman & Teachman, 2015). Thus, write-in options could be too cognitively demanding to feel enjoyable. This pattern of results could also reflect that write-in scenarios require participants to address more directly what they are anxious about in their own lives. Participants may not want to confront this personal anxiety trigger.

Conversely, written responses may promote engagement with the intervention (Steinman et al., 2021). By confronting anxiety and participating in approach (versus avoidance) behaviors, participants can learn how to tolerate and manage their anxiety (Butler et al., 1987; Rinck et al., 2013). It may be that microdoses appropriately activated some anxiety and negative affect in the short-term, but this may allow for valuable learning and opportunities for more positive affect in the long-term (Hofmann et al., 2008), akin to what occurs in exposure therapy. The observed differences in post-microdose affect between write-in and non-write-in scenarios may thus be a natural response to having to engage more deeply with the stressors participants are actually facing, which may benefit them in the long-term. However, the cognitive effort required of individuals in digital mental health interventions might not have positive in-the-moment effects, even though we presume that targeting individuals’ stressors more directly will have more potential benefits over time (see Hoppitt et al., 2014, about the benefits of actively engaging with anxious interpretations in CBM-I). Determining how to best balance the need to build skills in modifying unhelpful thinking in the moment with the natural desire to reduce distress and be able to focus on this skill-building is important, and may require sequencing brief interventions (e.g., completing a brief mindfulness exercise prior to engaging in CBM-I).

Consistent with these ideas, our results do not indicate a difference tied to writing demands on whether participants feel that the program helped them think about a situation in a new way or their ability to manage difficult emotions. This raises difficult questions about when to aim for short term positive feelings, which may promote engagement and adherence, and when to stick with the less positive short-term affective outcome because the training promotes more enduring, helpful learning opportunities (Hofmann et al., 2008). To our knowledge, this is the first study investigating the differential momentary outcomes of varying CBM-I scenario formats/writing demands. Given the field’s increasing focus on enhancing engagement/gamification of CBM-I (Salemink et al., 2022) and mHealth programs more broadly, these results can help inform how to adapt materials. For example, as adaptive intervention design and personalization advances, one could imagine assigning different training approaches throughout the day based on the user’s ability in that moment to tolerate more in-depth writing work versus their need for short-term affective relief. Specific to CBM-I, our results also provide preliminary evidence that we can offer a variety of writing exercises to help reduce the repetitive nature of training without compromising on the desired short-term cognitive changes across these formats.

### Limitations and Future Directions

The current study must be considered in light of its limitations. One limitation is that the sample size was determined based on power analyses conducted for the parent study; accordingly, we did not conduct a priori power analyses for the present analyses. Another limitation is the homogeneity of the participants. The majority identified as being White, non-Hispanic, and women, so our results are not representative of all races, ethnicities, and gender identities. Additionally, all participants are affiliated with UVA, which is a very niche sample of adults who study or work at the same institution in the same state, which is notably a historically Predominantly White Institution (PWI). While some studies show promising effects of CBM-I in minority racial and ethnic groups (Rozenman et al., 2020), the anxieties faced by these individuals can be unique and CBM-I may need more cultural tailoring (Carter et al., 2012; Safren et al., 2000). This may be especially true for stressors tied to discrimination as noted above. We also did not examine the effect of time or practice on our results. Some microdose types (e.g., write-your-own scenarios) occur later and less frequently in the microdose sequence within each domain, whereas others (e.g., one letter missing scenarios) occur much more frequently and earlier in the sequence. Accordingly, it is possible that certain results, such as the finding that writing scenarios were associated with significantly less positive affect change than non-writing scenarios, could be partially explained by an effect of practice or just decreasing engagement with the program over time.

Another potential limitation is that our sample had positive levels of affect pre-microdose, which might initially seem to suggest that people were feeling good and did not need mental health care when doing a session. This is an interesting and unexpected observation, especially when considering that participants were made aware of the study through recruitment materials highlighting the app’s potential in addressing feelings of worry, stress, anxious thinking, and depressed mood, and thus all participants presumably signed up for this program with that intent (though we acknowledge some participants may have signed up for other reasons, such as the financial compensation, but the pay rate was not high for the time required). Our qualitative work with users of the app (Livermon et al., 2025) has suggested there are different reasons someone might choose to initiate a microdose, which likely vary across and within persons over time. In some cases, we believe the time selected occurs because a person is feeling especially stressed and seeks help regulating difficult emotions. In other cases, it seems people feel it is a good time to work on building their skills, either because it is convenient or they feel in a good ‘head space’ to learn, etc. For example, one user told us during their qualitative post-study interview that they regularly did microdose sessions on the bus because it was a good time when there were few other distractions and they could reflect on how the training fit with challenges from the day (Livermon et al., 2025). Thus, we imagine that some sessions are completed at times of “vulnerability” (high stress), while others are completed at times of “intervention receptivity.” This distinction is discussed in the just-in-time adaptive intervention (JITAI) literature (Nahum-Shani et al., 2018, 2023), and we suspect that is part of why we do not reliably see high negative affect at baseline. Importantly, our goal is to provide an evidence-based resource for when individuals want to use it, which includes helping to manage stressors *before* they grow in intensity. We do not have a reason to believe that digital mental health interventions targeting anxious cognitions are only helpful when used during high levels of distress.

Though our study has important limitations, there are also strengths. First, we were able to tailor scenarios to students and staff members in a range of domains that our university community members specified as being particularly stressful. This allowed a new level of personalization with the goal of increasing engagement and effectiveness of the app. Second, using EMA measures collects participants’ feelings on a very short time-scale, allowing us to evaluate the moment-to-moment changes in affect and regulatory efficacy associated with a microdose of CBM-I, and reduce retrospective reporting bias. Additionally, we had a large number of microdoses completed (1,661), which granted us increased power to detect a potential effect of our microdose characteristics on post-microdose affect, reappraisal, and emotion regulation efficacy.

Clear future directions include comprehensive analyses of the relationship between momentary cognitive and affective outcomes of the Hoos Think Calmly application and adherence in the study period/engagement with the app, which the team is working on.

## Conclusion

Taken together, our findings point to mostly positive short-term effects from CBM-I microdoses, though not in all cases. On average, there was reliable positive change in participants’ affect scores from immediately before to after a microdose for six out of eight stressor domains and three out of five writing demand levels; people reported their reappraisal and emotion regulation efficacy as somewhat higher for those six stressor domains compared to romantic relationships and discrimination, but there were no differences in reappraisal and emotion regulation efficacies across writing demand levels.

## Supplementary Material

Supplement C

Supplement B

Supplement A

**Supplementary Information** The online version contains supplementary material available at https://doi.org/10.1007/s10608-025-10623-z.

## Figures and Tables

**Table 1:** Demographic characteristics

Characteristic	*n* (%)
Gender:	
Woman	79 (84.95%)
Man	12 (12.90%)
Other identity	1 (1.75%)
Transgender Man	0 (0%)
Transgender Woman	0 (0%)
Race:	
White/European Origin	65 (69.89%)
East Asian	8 (8.60%)
Other or Unknown	7 (7.53%)
South Asian	6 (6.45%)
Black/African Origin	4 (4.30%)
Participant selected more than one race	3 (3.23%)
American Indian/Alaska Native	0 (0%)
Native Hawaiian/Pacific Islander	0 (0%)
Ethnicity:	
Not Hispanic or Latino	89 (95.70%)
Hispanic or Latino	4 (4.30%)

**Table 2:** Descriptive statistics

	*n*	Mean	SD	Range
Completed Microdoses per Domain*		204.5	55.12	184
Academics/Work/Career Development	326			
Discrimination	142			
Family & Home Life	224			
Finances	166			
Mental Health	190			
Physical Health	210			
Romantic Relationships	192			
Social Situations	186			
Not available	1			
Completed Microdoses per Scenario Type*		232.4	289.30	696
Fill-in-the-blank	71			
Long scenario	72			
One-letter	720			
Two-letter	275			
Write-your-own	24			
Not available	475			
Post-Microdose Recommendations Received by Type*		412	59.10	107
Emotion Regulation Tip	344			
Resource/Referral	451			
Apply to Daily Life Tip	441			
Unknown (due to coding error)	401			
Ecological Momentary Assessment Scores				
Pre-Microdose EMA [out of 5]		4.26	0.94	4
Post-Microdose EMA [out of 7]		4.50	1.06	6
Cognitive Reappraisal Efficacy [out of 7]		4.28	1.42	6
Emotion Regulation Efficacy [out of 7]		4.52	1.22	6

* Mean, SD, and range based off of microdoses that have the data available

**Table 3:** Summary of outcomes

Predictor	Post-microdose affect (controlling for pre-microdose affect)	Cognitive reappraisal efficacy	Cognitive reappraisal efficacy (controlling for pre-microdose affect)	Emotion regulation efficacy	Emotion regulation efficacy (controlling for pre-microdose affect)
Stressor domain	√	√	√	√	√
Writing demand	√	X	N/A	X	N/A

A checkmark indicates that the test was significant, indicating there is a relationship between the two variables. An “X” indicates that the test was not significant, indicating there is not a reliable relationship between the two variables. “N/A” indicates that the test was not run

**Table 4:** Results for Hypothesis 1a: Scenario domain and affect score, Pairwise comparisons (Bolded values are significant at alpha 0.05)

Domain		*B*	SE	df	*t*	*P*
Academics/Work/Career	Discrimination	0.2736	0.0792	1613	3.456	**0.0118**
	Family/Home	0.0530	0.0658	1584	0.806	1.0000
	Finances	0.0455	0.0723	1576	0.630	1.0000
	Mental	0.0722	0.0689	1577	1.048	1.0000
	Physical	− 0.0754	0.0669	1581	− 1.127	1.0000
	Romantic	0.4230	0.0697	1593	6.073	**<.0001**
	Social Situations	0.0578	0.0696	1573	0.831	1.0000
Discrimination	Family/Home	− 0.2206	0.0832	1605	− 2.651	0.1621
	Finances	− 0.2280	0.0888	1606	− 2.568	0.1960
	Mental	− 0.2014	0.0863	1606	− 2.333	0.3359
	Physical	− 0.3489	0.0837	1598	− 4.169	**0.0007**
	Romantic	0.1494	0.0868	1610	1.722	1.0000
	Social Situations	− 0.2158	0.0869	1606	− 2.482	0.2368
Family & Home Life	Finances	− 0.0075	0.0768	1573	− 0.098	1.0000
	Mental	0.0192	0.0740	1570	0.259	1.0000
	Physical	− 0.1284	0.0720	1575	− 1.784	1.0000
	Romantic	0.3700	0.0746	1586	4.963	**<.0001**
	Social Situations	0.0048	0.0747	1577	0.064	1.0000
Finances	Mental	0.0267	0.0801	1576	0.334	1.0000
	Physical	− 0.1209	0.0779	1574	− 1.551	1.0000
	Romantic	0.3775	0.0800	1577	4.718	**0.0001**
	Social Situations	0.0123	0.0804	1574	0.153	1.0000
Mental Health	Physical	− 0.1476	0.0754	1572	− 1.958	0.8069
	Romantic	0.3508	0.0774	1580	4.530	**0.0001**
	Social Situations	− 0.0144	0.0782	1575	− 0.184	1.0000
Physical Health	Romantic	0.4984	0.0752	1577	6.629	**<.0001**
	Social Situations	0.1332	0.0757	1576	1.758	1.0000
Romantic Relationships	Social Situations	− 0.3652	0.0779	1580	− 4.687	**0.0001**

**Table 5:** Results for Hypothesis 1a: Scenario domain and affect score, Model comparison

	*npar*	AIC	BIC	Log Likelihood	Deviance	Chi-Square	Df	*P*
Baseline	4	3835.6	3857.2	− 1913.8	3827.6			
Random effects	11	3789.2	3848.6	− 1883.6	3767.2	60.478	7	<.0001

**Table 6:** Results for Hypothesis 1b: Scenario domain and emotion regulation efficacy, Pairwise comparisons (Bolded values are significant at alpha 0.05)

Domain		*B*	SE	df	*t*	*P*
Academics/Work/Career	Discrimination	0.5164	0.0972	1565	5.310	**<.0001**
	Family/Home	0.1041	0.0801	1552	1.299	1.0000
	Finances	0.0806	0.0878	1547	0.918	1.0000
	Mental	0.0208	0.0840	1548	0.248	1.0000
	Physical	− 0.0422	0.0814	1551	− 0.518	1.0000
	Romantic	0.3513	0.0851	1553	4.131	**0.0009**
	Social Situations	0.0555	0.0846	1544	0.656	1.0000
Discrimination	Family/Home	− 0.4123	0.1022	1562	− 4.036	**0.0013**
	Finances	− 0.4358	0.1091	1564	− 3.994	**0.0014**
	Mental	− 0.4955	0.1059	1562	− 4.680	**0.0001**
	Physical	− 0.5585	0.1026	1559	− 5.442	**<.0001**
	Romantic	− 0.1650	0.1066	1563	− 1.549	1.0000
	Social Situations	− 0.4609	0.1068	1561	− 4.317	**0.0004**
Family & Home Life	Finances	− 0.0235	0.0935	1549	− 0.251	1.0000
	Mental	− 0.0832	0.0901	1545	− 0.924	1.0000
	Physical	− 0.1462	0.0877	1549	− 1.667	1.0000
	Romantic	0.2473	0.0909	1552	2.719	0.1126
	Social Situations	− 0.0486	0.0910	1547	− 0.534	1.0000
Finances	Mental	− 0.0598	0.0975	1550	− 0.613	1.0000
	Physical	− 0.1228	0.0949	1551	− 1.293	1.0000
	Romantic	0.2707	0.0974	1549	2.781	0.0989
	Social Situations	− 0.0251	0.0979	1547	− 0.257	1.0000
Mental Health	Physical	− 0.0630	0.0917	1546	− 0.687	1.0000
	Romantic	0.3305	0.0944	1549	3.501	**0.0095**
	Social Situations	0.0346	0.0952	1546	0.364	1.0000
Physical Health	Romantic	0.3935	0.0915	1548	4.298	**0.0004**
	Social Situations	0.0976	0.0923	1547	1.058	1.0000
Romantic Relationships	Social Situations	− 0.2958	0.0949	1547	− 3.117	**0.0354**

**Table 7:** Results for Hypothesis 1b: Scenario domain and emotion regulation efficacy, Model comparisons

	*npar*	AIC	BIC	Log Likelihood	Deviance	Chi-Square	Df	*P*
Baseline	3	4561.0	4577.2	− 2277.5	4555.0			
Random effects	10	4524.5	4578.5	− 2252.2	4504.5	50.455	7	<.0001
Random effects controlling for affect	11	4386.9	4446.3	− 2182.4	4364.9	139.64	1	<.0001

**Table 8:** Results for Hypothesis 1c: Scenario domain and reappraisal, Pairwise comparisons (Bolded values are significant at alpha 0.05)

Domain		*B*	SE	df	*t*	*P*
Academics/Work/Career	Discrimination	0.8854	0.1130	1565	7.833	**<.0001**
	Family/Home	0.1302	0.0934	1552	1.393	1.0000
	Finances	0.0676	0.1024	1548	0.660	1.0000
	Mental	0.1521	0.0979	1549	1.554	1.0000
	Physical	0.0307	0.0949	1552	0.324	1.0000
	Romantic	0.4693	0.0992	1553	4.731	**0.0001**
	Social Situations	0.1035	0.0987	1545	1.049	1.0000
Discrimination	Family/Home	− 0.7552	0.1188	1562	− 5.357	**<.0001**
	Finances	− 0.8178	0.1269	1564	− 6.445	**<.0001**
	Mental	− 0.7332	0.1232	1563	− 5.952	**<.0001**
	Physical	− 0.8546	0.1194	1559	− 7.160	**<.0001**
	Romantic	− 0.4161	0.1239	1563	− 3.358	**0.0153**
	Social Situations	− 0.7819	0.1241	1561	− 6.300	**<.0001**
Family & Home Life	Finances	− 0.0626	0.1091	1549	− 0.574	1.0000
	Mental	− 0.0220	0.1050	1546	0.209	1.0000
	Physical	− 0.0994	0.1023	1550	− 0.972	1.0000
	Romantic	0.3391	0.1061	1553	3.197	**0.0240**
	Social Situations	− 0.0267	0.1061	1548	− 0.251	1.0000
Finances	Mental	0.0845	0.1137	1550	0.744	1.0000
	Physical	− 0.0368	0.1107	1551	− 0.333	1.0000
	Romantic	0.4017	0.1136	1549	3.537	**0.0083**
	Social Situations	0.0359	0.1142	1548	0.314	1.0000
Mental Health	Physical	− 0.1214	0.1070	1546	− 1.135	1.0000
	Romantic	0.3171	0.1101	1549	2.881	0.0644
	Social Situations	− 0.0486	0.1110	1546	− 0.438	1.0000
Physical Health	Romantic	0.4385	0.1068	1549	4.108	**0.0009**
	Social Situations	0.0727	0.1076	1548	0.676	1.0000
Romantic Relationships	Social Situations	− 0.3658	0.1107	1548	− 3.304	**0.0175**

**Table 9:** Results for Hypothesis 1c: Scenario domain and reappraisal, Model comparisons

	*npar*	AIC	BIC	Log Likelihood	Deviance	Chi-Square	Df	*P*
Baseline	3	5102.0	5118.2	− 2548.0	5096.0			
Random effects	10	5032.5	5086.5	− 2506.2	5012.5	83.532	7	<.0001
Random effects controlling for affect	11	4906.2	4965.6	− 2442.1	4884.2	128.25	1	<.0001

**Table 10:** Results for Hypotheses 2a and 2b: Writing demand and affect score, Pairwise comparisons with contrasts (Bolded values are significant at alpha 0.05)

Contrast	*B*	SE	df	*t*	*P*
No writing—Fill-in-the-blank	0.0304	0.0981	1113	0.310	0.7567
No writing—More writing	0.3073	0.0975	1122	3.152	**0.0017**
Fill-in-the-blank—More writing	0.2769	0.1296	1100	2.136	**0.0329**

**Table 11:** Results for Hypotheses 2a and 2b: Writing demand and affect score, Model comparison

	*npar*	AIC	BIC	Log Likelihood	Deviance	Chi-Square	Df	*P*
Baseline	4	2798.8	2819.1	−1395.4	2790.8			
Random effects	8	2792.6	2833.0	−1388.3	2776.6	14.299	4	0.0064

**Table 12:** Results for Hypotheses 2a and 2b: Writing demand and emotion regulation efficacy, Pairwise comparisons with grouping by contrasts

Contrast	*B*	SE	df	*t*	*P*
No writing—Fill-in-the-blank	0.0401	0.114	1089	0.352	0.7246
No writing—More writing	− 0.0794	0.114	1101	− 0.699	0.4850
Fill-in-the-blank—More writing	− 0.1194	0.150	1089	− 0.795	0.4266

**Table 13:** Results for Hypotheses 2a and 2b: Writing demand and emotion regulation efficacy, Model comparison

	*npar*	AIC	BIC	Log Likelihood	Deviance	Chi-Square	Df	*P*
Baseline	3	3214.6	3229.7	− 1604.3	3208.6			
Random effects	7	3213.5	3248.9	− 1599.7	3199.5	9.1184	4	0.05821

**Table 14:** Results for Hypotheses 2a and 2b: Writing demand and reappraisal, Pairwise comparisons with grouping by contrasts

Contrast	*B*	SE	df	*t*	*P*
No writing—Fill-in-the-blank	0.210	0.131	1087	1.600	0.11
No writing—More writing	− 0.136	0.131	1100	− 1.037	0.30
Fill-in-the-blank—More writing	− 0.346	0.173	1088	− 1.996	0.05

**Table 15:** Results for Hypotheses 2a and 2b: Writing demand and reappraisal, Model comparison

	*npar*	AIC	BIC	Log Likelihood	Deviance	Chi-Square	Df	*P*
Baseline	3	3551.6	3566.7	− 1772.8	3545.6			
Random effects	7	3552.7	3588.1	− 1769.3	3538.7	6.8512	4	0.144

**Table 16:** Pre- and post-microdose affect score means & model p-values for within-domain and within-writing demand analyses (Bolded values are significant at alpha 0.05)

Predictor		Mean (Pre)	Mean (Post)	*p*-value
Domain	Academics/Work/Career	4.14	4.53	**<.001**
	Discrimination	4.26	4.34	0.4368
	Family & Home Life	4.32	4.58	**<.001**
	Finances	4.39	4.62	**0.003**
	Mental Health	4.22	4.45	**0.002**
	Physical Health	4.31	4.71	**<.001**
	Romantic Relationships	4.14	4.05	0.3592
	Social Situations	4.36	4.66	**<.001**
Writing demand	One-letter missing	4.21	4.48	**<.001**
	Two-letters missing	4.29	4.65	**<.001**
	Fill-in-the-blank	4.13	4.46	**0.02262**
	Write your own	4.29	4.25	0.8469
	Long	4.29	4.38	0.5622

**Table 17:** Means and standard deviations of pre-microdose affect across stressor domains

Domain	Mean (SD) [out of 7]	Mean (SD) [out of 5]
Academics/Work/Career Development	4.90 (1.31)	4.14 (0.98)
Discrimination	5.17 (1.33)	4.26 (1.01)
Family & Home Life	5.10 (1.22)	4.32 (0.88)
Finances	5.17 (1.19)	4.39 (0.88)
Mental Health	4.99 (1.33)	4.22 (0.97)
Physical Health	5.10 (1.25)	4.31 (0.89)
Romantic Relationships	4.89 (1.35)	4.14 (1.05)
Social Situations	5.16 (1.21)	4.36 (0.83)

**Table 18:** Planned contrasts for Test 2: Writing demand

Substantive meaning: Compare write-in to fill in the blank to word fragment					
Writing demand:	Short–1 Tile	Short–2 Tiles	Fill-in-the-Blank	Write Your Own	Long
	0.5	0.5	−1	0	0
Contrast codes:	0.5	0.5	0	−0.5	−0.5
	0	0	−1	0.5	0.5

## Data Availability

The data used and statistical analysis scripts for this investigation, as well as the preregistration, are available in the Open Science Framework repository at https://osf.io/czyjm/.

## Footnotes

1 Note, we had initially also planned to report on the effect of different post-microdose recommendations (e.g., suggesting an emotion regulation tip vs. a referral or other resource), as noted in our preregistration, but based on feedback that the paper was covering too many features and needed to be condensed, we moved these analyses and discussion to Supplemental Material A.

## Appendix

See Table 18.
