# Supplementary material for: Examining State Affective and Cognitive Outcomes Following Brief Mobile Phone-Based Training Sessions to Reduce Anxious Interpretations
Source: Cognit Ther Res. 2025 Jun 16;50(1):96–118. doi: 10.1007/s10608-025-10623-z (PMC12890198; doi:10.1007/s10608-025-10623-z)
Supplement: Supplementary file 1 [file 10608_2025_10623_MOESM1_ESM.docx]

**Supplemental Material A: Secondary analyses**

Table of Contents

[Post-CBM-I Recommendation Outcome Variable Materials 6](#_Toc176377138)

[Methods 6](#_Toc176377139)

[Results with Pre-Microdose Affect Scores of 1-5 7](#_Toc176377140)

[Results with All Pre-Microdose Affect Scores (1-7) 8](#_Toc176377141)

[Discussion 9](#_Toc176377142)

[Tables 10](#_Toc176377143)

[Table A.1.1: Percent of total variance explained by fixed and random effects and intraclass correlation (ICC) for all models 10](#_Toc176377144)

[Table A.1.2: Results for Hypothesis 1a: Post-CBM-I recommendation and affect score, Model results including pre-microdose affect scores 1-5 11](#_Toc176377145)

[Table A.1.3: Results for Hypothesis 1a: Post-CBM-I recommendation and emotion regulation efficacy, Model results including pre-microdose affect scores 1-5 12](#_Toc176377146)

[Table A.1.4: Results for Hypothesis 1b: Post-CBM-I recommendation and reappraisal, Model results including pre-microdose affect scores 1-5 13](#_Toc176377147)

[Table A.1.5: Results for Hypothesis 1a: Post-CBM-I recommendation and affect score, Model results including pre-microdose affect scores 1-7 14](#_Toc176377148)

[Table A.1.6: Results for Hypothesis 1a: Post-CBM-I recommendation and emotion regulation efficacy, Model results including pre-microdose affect scores 1-7 15](#_Toc176377149)

[Table A.1.7: Results for Hypothesis 1b: Post-CBM-I recommendation and reappraisal efficacy, Model results including pre-microdose affect scores 1-7 16](#_Toc176377150)

[Table A.1.8a: Results for Hypothesis 1a: Post-CBM-I recommendation and affect score, Pairwise comparisons including pre-microdose affect scores 1-5 17](#_Toc176377151)

[Table A.1.8b: Results for Hypothesis 1a: Post-CBM-I recommendation and affect score, Model comparison including pre-microdose affect scores 1-5 17](#_Toc176377152)

[Table A.1.9a: Results for Hypothesis 1a: Post-CBM-I recommendation and emotion regulation efficacy, Pairwise comparisons including pre-microdose affect scores 1-5 18](#_Toc176377153)

[Table A.1.9b: Results for Hypothesis 1a: Post-CBM-I recommendation and emotion regulation efficacy, Model comparison including pre-microdose affect scores 1-5 18](#_Toc176377154)

[Table A.1.10a: Results for Hypothesis 1b: Post-CBM-I recommendation and reappraisal efficacy, Pairwise comparisons including pre-microdose affect scores 1-5 18](#_Toc176377155)

[Table A.1.10b: Results for Hypothesis 1b: Post-CBM-I recommendation and reappraisal efficacy, Model comparison including pre-microdose affect scores 1-5 19](#_Toc176377156)

[Table A.1.11a: Results for Hypothesis 1a: Post-CBM-I recommendation and affect score, Pairwise comparisons including pre-microdose affect scores 1-7 19](#_Toc176377157)

[Table A.1.11b: Results for Hypothesis 1a: Post-CBM-I recommendation and affect score, Model comparison including pre-microdose affect scores 1-7 19](#_Toc176377158)

[Table A.1.12a: Results for Hypothesis 1a: Post-CBM-I recommendation and emotion regulation efficacy, Pairwise comparisons including pre-microdose affect scores 1-7 20](#_Toc176377159)

[Table A.1.12b: Results for Hypothesis 1a: Post-CBM-I recommendation and emotion regulation efficacy, Model comparison including pre-microdose affect scores 1-7 20](#_Toc176377160)

[Table A.1.13a: Results for Hypothesis 1b: Post-CBM-I recommendation and reappraisal efficacy, Pairwise comparisons including pre-microdose affect scores 1-7 20](#_Toc176377161)

[Table A.1.13b: Results for Hypothesis 1b: Post-CBM-I recommendation and reappraisal efficacy, Model comparison including pre-microdose affect scores 1-7 21](#_Toc176377162)

[Faculty Descriptives & Analyses including pre-microdose affect scores of 1-5 21](#_Toc176377163)

[Results 21](#_Toc176377164)

[Tables 24](#_Toc176377165)

[Table A.2.1: Demographic Characteristics 24](#_Toc176377166)

[Table A.2.2: Descriptive Statistics 24](#_Toc176377167)

[Table A.2.3: Percent of total variance explained by fixed and random effects and intraclass correlation (ICC) for all models 25](#_Toc176377168)

[Table A.2.4a: Results for Hypothesis 1a: Scenario domain and affect score, Model results 26](#_Toc176377169)

[Table A.2.4b: Results for Hypothesis 1a: Scenario domain and affect score, Pairwise comparisons 28](#_Toc176377170)

[Table A.2.4c: Results for Hypothesis 1a: Scenario domain and affect score, Model comparison 29](#_Toc176377171)

[Table A.2.5a: Results for Hypothesis 1b: Scenario domain and emotion regulation efficacy, Model results 29](#_Toc176377172)

[Table A.2.5b: Results for Hypothesis 1b: Scenario domain and emotion regulation efficacy, Pairwise 31](#_Toc176377173)

[comparisons 31](#_Toc176377174)

[Table A.2.5c: Results for Hypothesis 1b: Scenario domain and emotion regulation efficacy, Model comparisons 32](#_Toc176377175)

[Table A.2.6a: Results for Hypothesis 1c: Scenario domain and reappraisal efficacy, Model results 33](#_Toc176377176)

[Table A.2.6b: Results for Hypothesis 1c: Scenario domain and reappraisal efficacy, Pairwise comparisons 34](#_Toc176377177)

[Table A.2.6c: Results for Hypothesis 1c: Scenario domain and reappraisal efficacy, Model comparisons 36](#_Toc176377178)

[Table A.2.7a: Results for Hypotheses 2a and 2b: Writing demand and affect score, Model results 36](#_Toc176377179)

[Table A.2.7b: Results for Hypotheses 2a and 2b: Writing demand and affect score, Pairwise comparisons with contrasts 37](#_Toc176377180)

[Table A.2.7c: Results for Hypotheses 2a and 2b: Writing demand and affect score, Model comparison 37](#_Toc176377181)

[Table A.2.8a: Results for Hypotheses 2a and 2b: Writing demand and emotion regulation efficacy, Model results 38](#_Toc176377182)

[Table A.2.8b: Results for Hypotheses 2a and 2b: Writing demand and emotion regulation efficacy, Pairwise comparisons with grouping by contrasts 39](#_Toc176377183)

[Table A.2.8c: Results for Hypotheses 2a and 2b: Writing demand and emotion regulation efficacy, Model comparison 39](#_Toc176377184)

[Table A.2.9a: Results for Hypotheses 2a and 2b: Writing demand and reappraisal efficacy, Model results 39](#_Toc176377185)

[Table A.2.9b: Results for Hypotheses 2a and 2b: Writing demand and reappraisal efficacy, Pairwise comparisons with grouping by contrasts 41](#_Toc176377186)

[Table 2.9c: Results for Hypotheses 2a and 2b: Writing demand and reappraisal efficacy, Model comparison 41](#_Toc176377187)

[Table A.2.10a: Results for Hypothesis 3a: Post-CBM-I recommendation and affect score, Model results 41](#_Toc176377188)

[Table A.2.10b: Results for Hypothesis 3a: Post-CBM-I recommendation and affect score, Pairwise comparisons 42](#_Toc176377189)

[Table A.2.10c: Results for Hypothesis 3a: Post-CBM-I recommendation and affect score, Model comparison 43](#_Toc176377190)

[Table A.2.11a: Results for Hypothesis 3a: Post-CBM-I recommendation and emotion regulation efficacy, Model results 43](#_Toc176377191)

[Table A.2.11b: Results for Hypothesis 3a: Post-CBM-I recommendation and emotion regulation efficacy, Pairwise comparisons 44](#_Toc176377192)

[Table A.2.11c: Results for Hypothesis 3a: Post-CBM-I recommendation and emotion regulation efficacy, Model comparison 44](#_Toc176377193)

[Table A.2.12a: Results for Hypothesis 3b: Post-CBM-I recommendation and reappraisal efficacy, Model results 44](#_Toc176377194)

[Table A.2.12b: Results for Hypothesis 3b: Post-CBM-I recommendation and reappraisal efficacy, Pairwise comparisons 45](#_Toc176377195)

[Table A.2.12c: Results for Hypothesis 3b: Post-CBM-I recommendation and reappraisal efficacy, Model comparison 46](#_Toc176377196)

[Faculty Descriptives & Analyses including all pre-microdose affect scores (1-7) 46](#_Toc176377197)

[Results 46](#_Toc176377198)

[Tables 50](#_Toc176377199)

[Table A.3.1: Demographic Characteristics 50](#_Toc176377200)

[Table A.3.2: Descriptive Statistics 50](#_Toc176377201)

[Table A.3.3: Percent of total variance explained by fixed and random effects and intraclass correlation (ICC) for all models 51](#_Toc176377202)

[Table A.3.4a: Results for Hypothesis 1a: Scenario domain and affect score, Model results 52](#_Toc176377203)

[Table A.3.4b: Results for Hypothesis 1a: Scenario domain and affect score, Pairwise comparisons 54](#_Toc176377204)

[Table A.3.4c: Results for Hypothesis 1a: Scenario domain and affect score, Model comparison 55](#_Toc176377205)

[Table A.3.5a: Results for Hypothesis 1b: Scenario domain and emotion regulation efficacy, Model results 56](#_Toc176377206)

[Table A.3.5b: Results for Hypothesis 1b: Scenario domain and emotion regulation efficacy, Pairwise 58](#_Toc176377207)

[comparisons 58](#_Toc176377208)

[Table A.3.5c: Results for Hypothesis 1b: Scenario domain and emotion regulation efficacy, Model comparisons 59](#_Toc176377209)

[Table A.3.6a: Results for Hypothesis 1c: Scenario domain and reappraisal efficacy, Model results 60](#_Toc176377210)

[Table A.3.6c: Results for Hypothesis 1c: Scenario domain and reappraisal efficacy, Model comparisons 63](#_Toc176377211)

[Table A.3.7a: Results for Hypotheses 2a and 2b: Writing demand and affect score, Model results 64](#_Toc176377212)

[Table A.3.7b: Results for Hypotheses 2a and 2b: Writing demand and affect score, Pairwise comparisons with contrasts 65](#_Toc176377213)

[Table A.3.7c: Results for Hypotheses 2a and 2b: Writing demand and affect score, Model comparison 65](#_Toc176377214)

[Table A.3.8a: Results for Hypotheses 2a and 2b: Writing demand and emotion regulation efficacy, Model results 66](#_Toc176377215)

[Table A.3.8b: Results for Hypotheses 2a and 2b: Writing demand and emotion regulation efficacy, Pairwise comparisons with grouping by contrasts 67](#_Toc176377216)

[Table A.3.8c: Results for Hypotheses 2a and 2b: Writing demand and emotion regulation efficacy, Model comparison 67](#_Toc176377217)

[Table A.3.9a: Results for Hypotheses 2a and 2b: Writing demand and reappraisal efficacy, Model results 67](#_Toc176377218)

[Table A.3.9b: Results for Hypotheses 2a and 2b: Writing demand and reappraisal efficacy, Pairwise comparisons with grouping by contrasts 68](#_Toc176377219)

[Table A.3.9c: Results for Hypotheses 2a and 2b: Writing demand and reappraisal efficacy, Model comparison 69](#_Toc176377220)

[Table A.3.10a: Results for Hypothesis 3a: Post-CBM-I recommendation and affect score, Model results 69](#_Toc176377221)

[Table A.3.10b: Results for Hypothesis 3a: Post-CBM-I recommendation and affect score, Pairwise comparisons 70](#_Toc176377222)

[Table A.3.10c: Results for Hypothesis 3a: Post-CBM-I recommendation and affect score, Model comparison 70](#_Toc176377223)

[Table A.3.11a: Results for Hypothesis 3a: Post-CBM-I recommendation and emotion regulation efficacy, Model results 71](#_Toc176377224)

[Table A.3.11b: Results for Hypothesis 3a: Post-CBM-I recommendation and emotion regulation efficacy, Pairwise comparisons 72](#_Toc176377225)

[Table A.3.11c: Results for Hypothesis 3a: Post-CBM-I recommendation and emotion regulation efficacy, Model comparison 72](#_Toc176377226)

[Table A.3.12a: Results for Hypothesis 3b: Post-CBM-I recommendation and reappraisal efficacy, Model results 72](#_Toc176377227)

[Table A.3.12b: Results for Hypothesis 3b: Post-CBM-I recommendation and reappraisal efficacy, Pairwise comparisons 73](#_Toc176377228)

[Table A.3.12c: Results for Hypothesis 3b: Post-CBM-I recommendation and reappraisal efficacy, Model comparison 73](#_Toc176377229)

# Post-CBM-I Recommendation Outcome Variable Materials

## **Methods**

**Variables**

Below are examples of each of these messages that a participant might have received as a follow-up to a microdose:

● ***Emotion Regulation Tip:*** “Try the STOPP method: S-Stop and pause for a moment. T- Take a breath. O- Observe what you're feeling. P- Pull back and put the situation into perspective. P- Practice what works, do something you enjoy/something that calms you.”

● ***Resource/Referral:*** “Check out CAVA for counseling needs regarding the impact of mental illness, substance abuse, trauma, and/or other life stressors. CAVA offers a variety of clinical specialists who practice several different approaches to treatment.”

● ***Applying Lessons Learned:*** “Think about a challenging situation that's coming up soon. What are some ways you could apply what you learned from this training to manage your own situation?”

**Hypotheses**

We will examine whether there is a significant difference in our outcome variables across microdoses with different post-CBM-I recommendations (i.e., emotion regulation tip; resource/referral; or applying lessons in daily life). As previously stated, we have competing hypotheses about the immediate effects of CBM-I on affect. However, emotion regulation tips give in-the-moment strategies for managing difficult emotions. Thus, we predict that that:

**Hypothesis 1a:** Momentary affect post-microdose and emotion regulation efficacy will be greater/more positive following microdoses including an emotion regulation tip compared to resources/referrals or application strategies.

The tips for applying lessons in daily life directly prompt participants to reflect on how they can use mechanisms from CBM-I training in their own situations. This encourages participants to think flexibly about events in their own lives. Thus, we predict that:

**Hypothesis 1b:** Reappraisal efficacy will be greater following microdoses including an application strategy compared to resources/referrals or emotion regulation tips.

**Statistical Analyses**

After building our baseline models for each outcome variable (i.e., post-microdose affect, reappraisal, emotion regulation efficacy), post-CBM-I recommendation type was added into the random intercepts models as a categorical predictor, and the *emmeans* package will be used to explore all pairwise differences with a Holm correction.

The total variances explained by the fixed effects and the total variances explained by both fixed and random effects, as well as the intraclass correlation coefficients (ICCs), are reported in Table A.1.1. Additionally, all null and random intercepts model results are reported in Tables A.1.2- A.1.7.

## **Results with Pre-Microdose Affect Scores of 1-5**

**How did post-microdose affect scores (controlling for pre-microdose affect) differ based on post-CBM-I recommendation? (H1a)**

Post-CBM-I recommendation data was collected for 81 (out of 100) participants, including 1,236 (out of 1,637) microdoses. Analyses were conducted only on the subset of microdoses that contain post-CBM-I recommendation data.

There was no significant difference between post-microdose affect scores across post-CBM-I recommendation types. See Table A.1.8a for pairwise comparisons. The random intercepts model with post-CBM-I recommendation as a predictor did not perform significantly better than the null model with no predictor (Chi-square=5.15, df=2, *P*=.076). See Table A.1.8b for full model comparison.

**How did post-microdose emotion regulation efficacy scores differ based on post-CBM-I recommendation? (H1a)**

There was no significant difference between post-microdose emotion regulation efficacy scores across post-CBM-I recommendation types. See Table A.1.9a for pairwise comparisons. The random intercepts model with post-CBM-I recommendation types as a predictor did not perform significantly better than the null model with no predictor (Chi-square=4.25, df=2, *P*=.12). See Table A.1.9b for full model comparison.

**How did post-microdose reappraisal efficacy scores differ based on post-CBM-I recommendation? (H1b)**

There was no significant difference in reappraisal efficacy scores across post-CBM-I recommendation types. See Table A.1.10a for pairwise comparisons. The random intercepts model with post-CBM-I recommendation types as a predictor did not perform significantly better than the null model with no predictor (Chi-square=1.30, df=2, *P*=.53). See Table A.1.10b for full model comparison.

## **Results with All Pre-Microdose Affect Scores (1-7)**

**How did post-microdose affect scores (controlling for pre-microdose affect) differ based on post-CBM-I recommendation? (H1a)**

Post-CBM-I recommendation data was collected for 85 (out of 105) participants, including 1,910 (out of 2,660) microdoses. Analyses were conducted only on the subset of microdoses that contain post-CBM-I recommendation data.

There was no significant difference between post-microdose affect scores across post-CBM-I recommendation types. See Table A.1.11a for pairwise comparisons. The random intercepts model with post-CBM-I recommendation as a predictor did not perform significantly better than the null model with no predictor (Chi-square=4.90, df=2, *P*=.086). See Table A.1.11b for full model comparison.

**How did post-microdose emotion regulation efficacy scores differ based on post-CBM-I recommendation? (H1a)**

There was no significant difference between post-microdose emotion regulation efficacy scores across post-CBM-I recommendation types. See Table A.1.12a for pairwise comparisons. However, the random intercepts model with post-CBM-I recommendation as a predictor performed significantly better than the null model with no predictor (Chi-square=6.63, df=2, *P*=.04), but only explained 0.2% more variance than the null model. See Table A.1.12b for full model comparison.

**How did post-microdose reappraisal efficacy scores differ based on post-CBM-I recommendation? (H1b)**

There was no significant difference in reappraisal efficacy scores across post-CBM-I recommendation types. See Table A.1.13a for pairwise comparisons. The random intercepts model with post-CBM-I recommendation types as a predictor did not perform significantly better than the null model with no predictor (Chi-square=1.90, df=2, *P*=.39). See Table A.1.13b for full model comparison.

## **Discussion**

Contrary to hypotheses, post-CBM-I recommendation was not a significant predictor of affect, reappraisal efficacy, or emotion regulation efficacy. The three recommendation types vary in the content provided to participants (e.g., emotional, cognitive, practical) and map onto our post-microdose survey questions well, so it is surprising to see no significant differences. Timing of the recommendations may help explain the lack of differences across recommendation types. Specifically, post-CBM-I recommendations are presented immediately before the post-EMA survey, so participants still have a brief survey to fill out before their microdose is complete. Thus, participants might not have tried the strategies or tips given in the recommendations prior to when they reported on their post-microdose states due to the app sequence. Recommendations may have been helpful but used outside of when we assessed the participants’ outcomes.

Specific aspects of the different recommendation types may also help explain why we failed to observe an association between recommendation type and post-microdose state outcomes. Considering that participants felt generally positive both before and after microdoses on average, participants may not have needed to use the recommended emotion regulation strategies in the moment, and therefore may not have felt that they feel any more or less able to manage difficult emotions. Indeed, one ecological momentary assessment study found that people often report feeling as though they do not need to regulate or change their emotions in daily life (Daniel et al., 2020). The tips to apply lessons learned revolve around teaching participants how to use the cognitive processes involved in CBM-I in their daily lives, and like mentioned before, participants likely are not applying these tips to any real-life situations in the time between seeing the tip and filling out the post-microdose survey. Thus, tips to apply lessons learned may reduce negative interpretation biases in the long term, but not strongly impact affect or reappraisal efficacy in the moment. Resources and referrals are inherently not usable in-the-moment, as participants would likely complete their microdose before looking into a resource, as mentioned above. Additionally, many of the resources provided are future-oriented and require planning, such as meeting with psychological services providers, joining clubs or other groups, or scheduling advising or other meetings. Thus, these recommendations may not change participants’ affect, or their feelings about reappraisal or emotion regulation efficacy, in the short length of a microdose.

## **Tables**

### **Table A.1.1:** *Percent of total variance explained by fixed and random effects and intraclass correlation (ICC) for all models*

|  |  | Fixed effects | Fixed and random effects | ICC |
| --- | --- | --- | --- | --- |
| Test | Model |  |  |  |
| Recommendation and affect score (1-5) | Null | 36.42% | 49.31% | 0.203 |
|  | Random Intercepts | 36.59% | 49.43% | 0.202 |
| Recommendation and ER efficacy (1-5) | Null | 0.00% | 50.41% | 0.504 |
|  | Random Intercepts | 0.18% | 50.57% | 0.505 |
| Recommendation and reappraisal efficacy (1-5) | Null | 0.00% | 52.61% | 0.526 |
|  | Random Intercepts | 0.05% | 52.54% | 0.525 |
| Recommendation and affect score (1-7) | Null | 47.86% | 56.33% | 0.162 |
|  | Random Intercepts | 47.92% | 56.39% | 0.163 |
| Recommendation and ER efficacy (1-7) | Null | 0.00% | 54.75% | 0.547 |
|  | Random Intercepts | 0.16% | 54.84% | 0.548 |
| Recommendation and reappraisal efficacy (1-7) | Null | 0.00% | 54.20% | 0.542 |
|  | Random Intercepts | 0.05% | 54.19% | 0.542 |

### **Table A.1.2***: Results for Hypothesis 1a: Post-CBM-I recommendation and affect score, Model results including pre-microdose affect scores 1-5*

| Random Effects |  |  |  |  |
| --- | --- | --- | --- | --- |
|  | Groups | Name | Variance | SD |
| *Null model* |  |  |  |  |
|  | ParticipantID | Intercept | 0.1497 | 0.3869 |
|  | Residual |  | 0.5890 | 0.7675 |
| *Random intercepts model* |  |  |  |  |
|  | ParticipantID | Intercept | 0.1492 | 0.3862 |
|  | Residual |  | 0.5876 | 0.7665 |

| Fixed Effects |  |  |  |  |
| --- | --- | --- | --- | --- |
|  |  | *B* | SE | *t* |
| *Null model* |  |  |  |  |
|  | Intercept | 1.67601 | 0.11820 | 14.18 |
|  | PreEMA | 0.67925 | 0.02537 | 26.78 |
| *Random intercepts model* |  |  |  |  |
|  | Intercept | 1.72615 | 0.12383 | 13.940 |
|  | PreEMA | 0.67838 | 0.02534 | 26.774 |
|  | Resource | -0.11195 | 0.05569 | -2.010 |
|  | Tip | -0.01517 | 0.05642 | -0.269 |

### **Table A.1.3:** *Results for Hypothesis 1a: Post-CBM-I recommendation and emotion regulation efficacy, Model results including pre-microdose affect scores 1-5*

| Random Effects |  |  |  |  |
| --- | --- | --- | --- | --- |
|  | Groups | Name | Variance | SD |
| *Null model* |  |  |  |  |
|  | ParticipantID | Intercept | 0.7787 | 0.8824 |
|  | Residual |  | 0.7661 | 0.8753 |
| *Random intercepts model* |  |  |  |  |
|  | ParticipantID | Intercept | 0.7796 | 0.8829 |
|  | Residual |  | 0.7645 | 0.8744 |

| Fixed Effects |  |  |  |  |
| --- | --- | --- | --- | --- |
|  |  | *B* | SE | *t* |
| *Null model* |  |  |  |  |
|  | Intercept | 4.5596 | 0.1042 | 43.75 |
| *Random intercepts model* |  |  |  |  |
|  | Intercept | 4.61333 | 0.11220 | 41.116 |
|  | Resource | -0.12059 | 0.06380 | -1.890 |
|  | Tip | -0.02509 | 0.06482 | -0.387 |

### **Table A.1.4:** *Results for Hypothesis 1b: Post-CBM-I recommendation and reappraisal, Model results including pre-microdose affect scores 1-5*

| Random Effects |  |  |  |  |
| --- | --- | --- | --- | --- |
|  | Groups | Name | Variance | SD |
| *Null model* |  |  |  |  |
|  | ParticipantID | Intercept | 1.142 | 1.069 |
|  | Residual |  | 1.029 | 1.014 |
| *Random intercepts model* |  |  |  |  |
|  | ParticipantID | Intercept | 1.138 | 1.067 |
|  | Residual |  | 1.029 | 1.015 |

| Fixed Effects |  |  |  |  |
| --- | --- | --- | --- | --- |
|  |  | *B* | SE | *t* |
| *Null model* |  |  |  |  |
|  | Intercept | 4.3908 | 0.1257 | 34.94 |
| *Random intercepts model* |  |  |  |  |
|  | Intercept | 4.44406 | 0.13440 | 33.065 |
|  | Resource | -0.06391 | 0.07405 | -0.863 |
|  | Tip | -0.08264 | 0.07523 | -1.098 |

### **Table A.1.5***: Results for Hypothesis 1a: Post-CBM-I recommendation and affect score, Model results including pre-microdose affect scores 1-7*

| Random Effects |  |  |  |  |
| --- | --- | --- | --- | --- |
|  | Groups | Name | Variance | SD |
| *Null model* |  |  |  |  |
|  | ParticipantID | Intercept | 0.1152 | 0.3394 |
|  | Residual |  | 0.5938 | 0.7706 |
| *Random intercepts model* |  |  |  |  |
|  | ParticipantID | Intercept | 0.1152 | 0.3395 |
|  | Residual |  | 0.5928 | 0.7700 |

| Fixed Effects |  |  |  |  |
| --- | --- | --- | --- | --- |
|  |  | *B* | SE | *t* |
| *Null model* |  |  |  |  |
|  | Intercept | 1.83921 | 0.08899 | 20.67 |
|  | PreEMA | 0.63705 | 0.01584 | 40.21 |
| *Random intercepts model* |  |  |  |  |
|  | Intercept | 1.868939 | 0.093741 | 19.937 |
|  | PreEMA | 0.636442 | 0.015834 | 40.194 |
|  | Resource | -0.079478 | 0.044998 | -1.766 |
|  | Tip | 0.004423 | 0.045243 | 0.098 |

### **Table A.1.6:** *Results for Hypothesis 1a: Post-CBM-I recommendation and emotion regulation efficacy, Model results including pre-microdose affect scores 1-7*

| Random Effects |  |  |  |  |
| --- | --- | --- | --- | --- |
|  | Groups | Name | Variance | SD |
| *Null model* |  |  |  |  |
|  | ParticipantID | Intercept | 1.0462 | 1.0228 |
|  | Residual |  | 0.8647 | 0.9299 |
| *Random intercepts model* |  |  |  |  |
|  | ParticipantID | Intercept | 1.0446 | 1.0220 |
|  | Residual |  | 0.8626 | 0.9288 |

| Fixed Effects |  |  |  |  |
| --- | --- | --- | --- | --- |
|  |  | *B* | SE | *t* |
| *Null model* |  |  |  |  |
|  | Intercept | 4.7029 | 0.1144 | 41.1 |
| *Random intercepts model* |  |  |  |  |
|  | Intercept | 4.75934 | 0.11973 | 39.751 |
|  | Resource | -0.12888 | 0.05445 | -2.367 |
|  | Tip | -0.02781 | 0.05485 | -0.507 |

### **Table A.1.7:** *Results for Hypothesis 1b: Post-CBM-I recommendation and reappraisal efficacy, Model results including pre-microdose affect scores 1-7*

| Random Effects |  |  |  |  |
| --- | --- | --- | --- | --- |
|  | Groups | Name | Variance | SD |
| *Null model* |  |  |  |  |
|  | ParticipantID | Intercept | 1.360 | 1.166 |
|  | Residual |  | 1.149 | 1.072 |
| *Random intercepts model* |  |  |  |  |
|  | ParticipantID | Intercept | 1.072 | 1.165 |
|  | Residual |  | 1.149 | 1.072 |

| Fixed Effects |  |  |  |  |
| --- | --- | --- | --- | --- |
|  |  | *B* | SE | *t* |
| *Null model* |  |  |  |  |
|  | Intercept | 4.5387 | 0.1305 | 34.78 |
| *Random intercepts model* |  |  |  |  |
|  | Intercept | 4.5641238 | 0.1367104 | 33.385 |
|  | Resource | -0.0713315 | 0.0627687 | -1.136 |
|  | Tip | -0.0001608 | 0.0632486 | -0.003 |

### **Table A.1.8a:** *Results for Hypothesis 1a: Post-CBM-I recommendation and affect score, Pairwise comparisons including pre-microdose affect scores 1-5*

| Recommendation | *B* | SE | df | *t* | *P* |
| --- | --- | --- | --- | --- | --- |
| ER - Resource | 0.1119 | 0.0557 | 1181 | 2.010 | 0.1340 |
| ER - Tip | 0.0152 | 0.0564 | 1193 | 0.269 | 0.7881 |
| Resource - Tip | -0.0968 | 0.0523 | 1187 | -1.849 | 0.1340 |

### **Table A.1.8b:** *Results for Hypothesis 1a: Post-CBM-I recommendation and affect score, Model comparison including pre-microdose affect scores 1-5*

|  | *npar* | AIC | BIC | Log Likelihood | Deviance | Chi-Square | Df | *P* |
| --- | --- | --- | --- | --- | --- | --- | --- | --- |
| Baseline | 4 | 2972.0 | 2992.5 | -1482.0 | 2964.0 |  |  |  |
| Random effects | 6 | 2970.8 | 3001.6 | -1479.4 | 2958.8 | 5.1459 | 2 | 0.07631 |

### **Table A.1.9a:** *Results for Hypothesis 1a: Post-CBM-I recommendation and emotion regulation efficacy, Pairwise comparisons including pre-microdose affect scores 1-5*

###

| Recommendation | *B* | SE | df | *t* | *P* |
| --- | --- | --- | --- | --- | --- |
| ER - Resource | 0.1206 | 0.0638 | 1166 | 1.890 | 0.1771 |
| ER - Tip | 0.0251 | 0.0648 | 1171 | 0.387 | 0.6988 |
| Resource - Tip | -0.0955 | 0.0601 | 1169 | -1.590 | 0.2241 |

### **Table A.1.9b:** *Results for Hypothesis 1a: Post-CBM-I recommendation and emotion regulation efficacy, Model comparison including pre-microdose affect scores 1-5*

|  | *npar* | AIC | BIC | Log Likelihood | Deviance | Chi-Square | Df | *P* |
| --- | --- | --- | --- | --- | --- | --- | --- | --- |
| Baseline | 3 | 3385.9 | 3401.3 | -1690.0 | 3379.9 |  |  |  |
| Random effects | 5 | 3385.7 | 3411.3 | -1687.8 | 3375.7 | 4.2483 | 2 | 0.1195 |

### **Table A.1.10a:** *Results for Hypothesis 1b: Post-CBM-I recommendation and reappraisal efficacy, Pairwise comparisons including pre-microdose affect scores 1-5*

| Recommendation | *B* | SE | df | *t* | *P* |
| --- | --- | --- | --- | --- | --- |
| ER - Resource | 0.0639 | 0.0741 | 1165 | 0.863 | 0.8170 |
| ER - Tip | 0.0826 | 0.0752 | 1170 | 1.098 | 0.8170 |
| Resource - Tip | 0.0187 | 0.0697 | 1168 | 0.269 | 0.8170 |

### **Table A.1.10b:** *Results for Hypothesis 1b: Post-CBM-I recommendation and reappraisal efficacy, Model comparison including pre-microdose affect scores 1-5*

|  | *npar* | AIC | BIC | Log Likelihood | Deviance | Chi-Square | Df | *P* |
| --- | --- | --- | --- | --- | --- | --- | --- | --- |
| Baseline | 3 | 3756.6 | 3771.9 | -1875.3 | 3750.6 |  |  |  |
| Random effects | 5 | 3759.3 | 3784.9 | -1874.6 | 3749.3 | 1.2973 | 2 | 0.5228 |

### **Table A.1.11a:** *Results for Hypothesis 1a: Post-CBM-I recommendation and affect score, Pairwise comparisons including pre-microdose affect scores 1-7*

| Recommendation | *B* | SE | df | *t* | *P* |
| --- | --- | --- | --- | --- | --- |
| ER - Resource | 0.07948 | 0.0450 | 1842 | 1.766 | 0.1551 |
| ER - Tip | -0.00442 | 0.0453 | 1856 | -0.098 | 0.9221 |
| Resource - Tip | -0.08390 | 0.0420 | 1856 | -1.998 | 0.1377 |

### **Table A.1.11b:** *Results for Hypothesis 1a: Post-CBM-I recommendation and affect score, Model comparison including pre-microdose affect scores 1-7*

|  | *npar* | AIC | BIC | Log Likelihood | Deviance | Chi-Square | Df | *P* |
| --- | --- | --- | --- | --- | --- | --- | --- | --- |
| Baseline | 4 | 4560.1 | 4582.3 | -2276.1 | 4552.1 |  |  |  |
| Random effects | 6 | 4592.5 | 4592.5 | -2273.6 | 4547.2 | 4.9018 | 2 | 0.08622 |

### **Table A.1.12a:** *Results for Hypothesis 1a: Post-CBM-I recommendation and emotion regulation efficacy, Pairwise comparisons including pre-microdose affect scores 1-7*

| Recommendation | *B* | SE | df | *t* | *P* |
| --- | --- | --- | --- | --- | --- |
| ER - Resource | 0.1289 | 0.0545 | 1825 | 2.367 | 0.0542 |
| ER - Tip | 0.0278 | 0.0549 | 1830 | 0.507 | 0.6122 |
| Resource - Tip | -0.1011 | 0.0509 | 1830 | -1.987 | 0.0941 |

### **Table A.1.12b:** *Results for Hypothesis 1a: Post-CBM-I recommendation and emotion regulation efficacy, Model comparison including pre-microdose affect scores 1-7*

|  | *npar* | AIC | BIC | Log Likelihood | Deviance | Chi-Square | Df | *P* |
| --- | --- | --- | --- | --- | --- | --- | --- | --- |
| Baseline | 3 | 5405.5 | 5422.2 | -2699.7 | 5399.5 |  |  |  |
| Random effects | 5 | 5430.6 | 5430.6 | -2696.4 | 5392.9 | 6.6259 | 2 | 0.0364 |

### **Table A.1.13a:** *Results for Hypothesis 1b: Post-CBM-I recommendation and reappraisal efficacy, Pairwise comparisons including pre-microdose affect scores 1-7*

| Recommendation | *B* | SE | df | *t* | *P* |
| --- | --- | --- | --- | --- | --- |
| ER - Resource | 0.071332 | 0.0628 | 1828 | 1.136 | 0.6763 |
| ER - Tip | 0.000161 | 0.0633 | 1832 | 0.003 | 0.9980 |
| Resource - Tip | -0.071171 | 0.0587 | 1832 | -1.213 | 0.6763 |

### **Table A.1.13b:** *Results for Hypothesis 1b: Post-CBM-I recommendation and reappraisal efficacy, Model comparison including pre-microdose affect scores 1-7*

|  | *npar* | AIC | BIC | Log Likelihood | Deviance | Chi-Square | Df | *P* |
| --- | --- | --- | --- | --- | --- | --- | --- | --- |
| Baseline | 3 | 5951.8 | 5968.4 | -2972.9 | 5945.8 |  |  |  |
| Random effects | 5 | 5953.9 | 5981.7 | -2971.9 | 5943.9 | 1.8984 | 2 | 0.3871 |

# Faculty Descriptives & Analyses including pre-microdose affect scores of 1-5

## **Results**

**Demographics**

Six faculty member participants were included in our sample, and five of them provided demographic data; out of the nine faculty members in the full dataset, only six of them remain after removing all microdoses with pre-microdose affect scores of 6 or 7 (meaning three faculty members only ever reported affect scores of 6 and 7 before a microdose). All five participants identified as White/European (100%) and non-Hispanic (100%), and two-thirds of the participants identified as women (60%). Participant ages ranged from 35 to 54 (*M* = 45.75, *SD* = 8.88). See Table A.2.1 for full demographic information.

**Microdose Characteristics**

Across the full sample, participants completed 94 microdoses in total. The total number of microdoses completed by participants during their study participation ranged from two (2.4% of total expected) to 58 (over 100% of total expected) microdoses (*M* = 15.67, *SD* = 21.60). Due to a technical error with how the mobile application collected data at the beginning of the study (October 2022 - January 2023), writing demand and recommendation data are missing for a portion of the microdoses. Specifically, the microdose domain is known for all but one microdose (98.94%), writing demand is known for 85 microdoses (90.43%), and recommendation type is known for 87 microdoses (91.81%). On average, participants rated their affect as being somewhat positive to fairly neutral both immediately prior to (4.00 out of 7) and immediately after (3.86 out of 7) completing a microdose; participants rated their cognitive reappraisal efficacy (3.09 out of 7) as being fairly neutral (as opposed to better or worse) after completing a microdose; participants rated their their emotion regulation efficacy (4.73 out of 7) as being somewhat better immediately after completing a microdose. Family and Home Life was the most frequently chosen domain (*n =* 28); the least chosen domains were Discrimination (*n =* 3) and Romantic Relationships (*n =* 3). See Table A.2.2 for full descriptives.

For each model, the total variance explained by the fixed effects and the total variance explained by both fixed and random effects, as well as the intraclass correlation coefficient (ICC), is reported in Table A.2.3.

**How did post-microdose affect scores (controlling for pre-microdose affect) differ based on domain? (Hypothesis 1a)**

There was no significant difference in post-microdose affect scores across stressor domains. See Tables A.2.4a and A.2.4b for model results and pairwise comparisons. The random intercepts model with domain as a predictor did not perform significantly better than the null model with no predictor (Chi-square=3.6511, df=7, *P*=0.819). See Table A.2.4c for full model comparison, including Akaike Information Criterion (AIC) values.

**How did post-microdose emotion regulation efficacy differ based on domain? (Hypothesis 1b)**

There was no significant difference in emotion regulation efficacy scores across stressor domains. See Tables A.2.5a and A.2.5b for model results and pairwise comparisons, respectively. The random intercepts model with domain as a predictor did not perform significantly better than the null model with no predictor (Chi-square=8.6429, df=7, *P*=0.2793). See Table A.2.5c for full model comparisons.

**How did post-microdose reappraisal efficacy differ based on domain? (Hypothesis 1c)**

There was no significant difference in reappraisal efficacy scores across stressor domains. See Tables A.2.6a and A.2.6b for model results and pairwise comparisons, respectively. The random intercepts model with domain as a predictor did not perform significantly better than the null model with no predictor (Chi-square=6.7444, df=7, *P*=0.456). See Table A.2.6c for full model comparisons.

**How did post-microdose affect scores (controlling for pre-microdose affect) differ based on writing demand? (Hypotheses 2a and 2b)**

There was no significant difference in post-microdose affect scores across writing demands. See Tables A.2.7a and A.2.7b for model results and pairwise comparisons. The random intercepts model with writing demand as a predictor performed significantly better than the null model with no predictor (Chi-square=17.743, df=4, *P*=.001), but given the insignificant pairwise comparisons, we are concluding that there is insufficient evidence that writing extent is a good predictor of post-microdose affect score. See Table A.2.7c for full model comparison.

**How did post-microdose emotion regulation efficacy differ based on writing demand? (Hypotheses 2a and 2b)**

There was no significant difference in emotion regulation efficacy scores across writing demands. See Tables A.2.8a and A.2.8b for model results and pairwise comparisons. The random intercepts model with writing demand as a predictor did not perform significantly better than the null model with no predictor (Chi-square=4.1338, df=4, *P*=0.3882). See Table A.2.8c for full model comparison.

**How did post-microdose reappraisal efficacy differ based on writing demand? (Hypotheses 2a and 2b)**

There was no significant difference in reappraisal efficacy scores across writing demands. See Tables A.2.9a and A.2.9b for model results and pairwise comparisons. The random intercepts model with scenario format as a predictor did not perform significantly better than the null model with no predictor (Chi-square=1.3705, df=4, *P*=0.8493). See Table A.2.9c for full model comparison.

**How did post-microdose affect scores (controlling for pre-microdose affect) differ based on post-CBM-I recommendation? (Hypothesis 3a)**

There was no significant difference between post-microdose affect scores across post-CBM-I recommendation types. See Tables A.2.10a and A.2.10b for model results and pairwise comparisons. The random intercepts model with post-CBM-I recommendation as a predictor did not perform significantly better than the null model with no predictor (Chi-square=1.285, df=2, *P*=0.526). See Table A.2.10c for full model comparison.

**How did post-microdose emotion regulation efficacy scores differ based on post-CBM-I recommendation? (Hypothesis 3a)**

There was no significant difference between post-microdose emotion regulation efficacy scores across post-CBM-I recommendation types. See Tables A.2.11a and A.2.11b for model results and pairwise comparisons. The random intercepts model with post-CBM-I recommendation as a predictor did not perform significantly better than the null model with no predictor (Chi-square=0.1536, df=2, *P*=0.9261). See Table A.2.11c for full model comparison.

**How did post-microdose reappraisal efficacy scores differ based on post-CBM-I recommendation? (Hypothesis 3b)**

There was no significant difference in reappraisal efficacy scores across post-CBM-I recommendation types. See Tables A.2.12a and A.2.12b for model results and pairwise comparisons. The random intercepts model with post-CBM-I recommendation types as a predictor did not perform significantly better than the null model with no predictor (Chi-square=5.2429, df=2, *P*=0.07). See Table A.2.12c for full model comparison.

## **Tables**

| **Table A.2.1:** *Demographic Characteristics* |  |
| --- | --- |
| Characteristic | *n* (%) |
| Gender:  Man  Woman  Transgender Man  Transgender Woman  Other identity  Not Reported  Race:  White/European Origin  East Asian  South Asian  Other or Unknown  Black/African Origin  Participant selected more than one race  American Indian/Alaska Native  Native Hawaiian/Pacific Islander  Ethnicity:  Not Hispanic or Latino  Hispanic or Latino | 2 (40%)  3 (60%)  0 (0%)  0 (0%)  0 (0%)  0 (0%)  5 (100%)  0 (0%)  0 (0%)  0 (0%)  0 (0%)  0 (0%)  0 (0%)  0 (0%)  5 (100%)  0 (0%) |

### **Table A.2.2:** *Descriptive Statistics*

|  | *n* | Mean | SD | Range |
| --- | --- | --- | --- | --- |
| Microdoses per Domain*  Academics/Work/Career Development  Discrimination  Family & Home Life  Finances  Mental Health  Physical Health  Romantic Relationships  Social Situations  Not available | 9  3  28  4  25  9  3  12  1 | 10.44 | 9.80 | 27 |
| Microdoses per Scenario Type*  Fill-in-the-blank  Long scenario  One-letter  Two-letter  Write-your-own  Not available | 8  5  53  14  5  9 | 15.67 | 18.59 | 48 |
| Microdoses per Recommendation Type*  Emotion Regulation  Resource  Tip  Not available | 34  27  26  7 | 23.5 | 11.56 | 27 |
| Ecological Momentary Assessment Scores  Pre-Microdose EMA [out of 5]  Post-Microdose EMA [out of 7]  Cognitive Reappraisal Efficacy [out of 7]  Emotion Regulation Efficacy [out of 7]  *Mean, SD, and range based off of microdoses that have the data available |  | 4.00  3.86  3.09  4.73 | 0.79  1.03  1.37  1.68 | 4  6  6  6 |

### **Table A.2.3:** *Percent of total variance explained by fixed and random effects and intraclass correlation (ICC) for all models*

|  |  | Fixed effects | Fixed and random effects | ICC |
| --- | --- | --- | --- | --- |
| Test | Model |  |  |  |
| Scenario domain and affect score | Null | 22.44% | 46.71% | 0.313 |
|  | Random Intercepts | 23.63% | 45.95% | 0.292 |
| Scenario domain and ER efficacy | Null | 0.00% | 0.00% | 0.00 |
|  | Random Intercepts | 8.26% | 8.26% | 0.00 |
| Scenario domain and reappraisal efficacy | Null | 0.00% | 62.88% | 0.629 |
|  | Random Intercepts | 2.69% | 66.85% | 0.659 |
| Writing demand and affect score | Null | 19.34% | 44.09% | 0.307 |
|  | Random Intercepts | 34.94% | 51.49% | 0.254 |
| Writing demand and ER efficacy | Null | 0.00% | 0.00% | 0.00 |
|  | Random Intercepts | 4.53% | 4.53% | 0.00 |
| Writing demand and reappraisal efficacy | Null | 0.00% | 62.65% | 0.626 |
|  | Random Intercepts | 0.67% | 62.77% | 0.625 |
| Recommendation and affect score | Null | 21.54% | 47.36% | 0.329 |
|  | Random Intercepts | 21.92% | 47.60% | 0.329 |
| Recommendation and ER efficacy | Null | 0.00% | 0.00% | 0.00 |
|  | Random Intercepts | 0.17% | 0.17% | 0.00 |
| Recommendation and reappraisal efficacy | Null | 0.00% | 69.71% | 0.697 |
|  | Random Intercepts | 1.78% | 71.15% | 0.706 |

### **Table A.2.4a:** *Results for Hypothesis 1a: Scenario domain and affect score, Model results*

| Random Effects |  |  |  |  |
| --- | --- | --- | --- | --- |
|  | Groups | Name | Variance | SD |
| *Null model* |  |  |  |  |
|  | ParticipantID | Intercept | 0.2752 | 0.5246 |
|  | Residual |  | 0.6040 | 0.7772 |
| *Random intercepts model* |  |  |  |  |
|  | ParticipantID | Intercept | 0.2609 | 0.5107 |
|  | Residual |  | 0.6317 | 0.7948 |

| Fixed Effects |  |  |  |  |
| --- | --- | --- | --- | --- |
|  |  | *B* | SE | *t* |
| *Null model* |  |  |  |  |
|  | Intercept | 1.6825 | 0.5201 | 3.235 |
|  | PreEMA | 0.6352 | 0.1073 | 5.917 |
| *Random intercepts model* |  |  |  |  |
|  | Intercept | 1.92108 | 0.59938 | 3.205 |
|  | PreEMA | 0.60697 | 0.11144 | 5.446 |
|  | Discrimination | -0.27414 | 0.54894 | -0.499 |
|  | Family & Home Life | -0.11247 | 0.31124 | -0.361 |
|  | Finances | -0.16907 | 0.49718 | -0.340 |
|  | Mental Health | -0.34027 | 0.31395 | -1.084 |
|  | Physical Health | -0.03796 | 0.37677 | -0.101 |
|  | Romantic Relationships | -0.08287 | 0.53936 | -0.154 |
|  | Social Situations | 0.12347 | 0.35713 | 0.346 |

### **Table A.2.4b:** *Results for Hypothesis 1a: Scenario domain and affect score, Pairwise comparisons*

| Domain |  | *B* | SE | df | *t* | *P* |
| --- | --- | --- | --- | --- | --- | --- |
| Academics/Work/Career | Discrimination | 0.2741 | 0.553 | 81.7 | 0.495 | 1.0000 |
|  | Family/Home | 0.1125 | 0.312 | 80.4 | 0.360 | 1.0000 |
|  | Finances | 0.1691 | 0.501 | 81.7 | 0.337 | 1.0000 |
|  | Mental | 0.3403 | 0.316 | 81.4 | 1.078 | 1.0000 |
|  | Physical | 0.0380 | 0.377 | 80.0 | 0.101 | 1.0000 |
|  | Romantic | 0.0829 | 0.541 | 80.3 | 0.153 | 1.0000 |
|  | Social Situations | -0.1235 | 0.359 | 81.6 | -0.344 | 1.0000 |
| Discrimination | Family/Home | -0.1617 | 0.524 | 82.9 | -0.308 | 1.0000 |
|  | Finances | -0.1051 | 0.609 | 79.3 | -0.173 | 1.0000 |
|  | Mental | 0.0661 | 0.524 | 83.4 | 0.126 | 1.0000 |
|  | Physical | -0.2362 | 0.553 | 82.0 | -0.427 | 1.0000 |
|  | Romantic | -0.1913 | 0.662 | 80.9 | -0.289 | 1.0000 |
|  | Social Situations | -0.3976 | 0.559 | 83.6 | -0.712 | 1.0000 |
| Family & Home Life | Finances | 0.0566 | 0.469 | 83.0 | 0.121 | 1.0000 |
|  | Mental | 0.2278 | 0.226 | 81.4 | 1.009 | 1.0000 |
|  | Physical | -0.0745 | 0.317 | 80.9 | -0.235 | 1.0000 |
|  | Romantic | -0.0296 | 0.501 | 80.6 | -0.059 | 1.0000 |
|  | Social Situations | -0.2359 | 0.290 | 82.1 | -0.813 | 1.0000 |
| Finances | Mental | 0.1712 | 0.469 | 83.5 | 0.365 | 1.0000 |
|  | Physical | -0.1311 | 0.501 | 82.1 | -0.262 | 1.0000 |
|  | Romantic | -0.0862 | 0.616 | 80.5 | -0.140 | 1.0000 |
|  | Social Situations | -0.2925 | 0.505 | 83.8 | -0.580 | 1.0000 |
| Mental Health | Physical | -0.3023 | 0.314 | 80.7 | -0.961 | 1.0000 |
|  | Romantic | -0.2574 | 0.501 | 81.0 | -0.514 | 1.0000 |
|  | Social Situations | -0.4637 | 0.285 | 80.0 | -1.626 | 1.0000 |
| Physical Health | Romantic | 0.0449 | 0.542 | 80.6 | 0.083 | 1.0000 |
|  | Social Situations | -0.1614 | 0.359 | 81.3 | -0.450 | 1.0000 |
| Romantic Relationships | Social Situations | -0.2063 | 0.536 | 81.7 | -0.385 | 1.0000 |

### **Table A.2.4c:** *Results for Hypothesis 1a: Scenario domain and affect score, Model comparison*

|  | *npar* | AIC | BIC | Log Likelihood | Deviance | Chi-Square | Df | *P* |
| --- | --- | --- | --- | --- | --- | --- | --- | --- |
| Baseline | 4 | 232.67 | 242.80 | -112.33 | 224.67 |  |  |  |
| Random effects | 11 | 243.01 | 270.87 | -110.51 | 221.01 | 3.6511 | 7 | 0.819 |

### **Table A.2.5a:** *Results for Hypothesis 1b: Scenario domain and emotion regulation efficacy, Model results*

| Random Effects |  |  |  |  |
| --- | --- | --- | --- | --- |
|  | Groups | Name | Variance | SD |
| *Null model* |  |  |  |  |
|  | ParticipantID | Intercept | 0.000 | 0.000 |
|  | Residual |  | 2.846 | 1.687 |
| *Random intercepts model* |  |  |  |  |
|  | ParticipantID | Intercept | 0.000 | 0.000 |
|  | Residual |  | 2.807 | 1.675 |

| Fixed Effects |  |  |  |  |
| --- | --- | --- | --- | --- |
|  |  | *B* | SE | *t* |
| *Null model* |  |  |  |  |
|  | Intercept | 4.7419 | 0.1749 | 27.11 |
| *Random intercepts model* |  |  |  |  |
|  | Intercept | 5.0000 | 0.5584 | 8.953 |
|  | Discrimination | -0.6667 | 1.1169 | -0.597 |
|  | Family & Home Life | 0.1071 | 0.6419 | 0.167 |
|  | Finances | -0.5000 | 1.0067 | -0.497 |
|  | Mental Health | -0.7600 | 0.6513 | -1.167 |
|  | Physical Health | -0.6667 | 0.7898 | -0.844 |
|  | Romantic Relationships | -1.3333 | 1.1169 | -1.194 |
|  | Social Situations | 0.5000 | 0.7388 | 0.677 |

### **Table A.2.5b:** *Results for Hypothesis 1b: Scenario domain and emotion regulation efficacy, Pairwise*

### comparisons

| Domain |  | *B* | SE | df | *t* | *P* |
| --- | --- | --- | --- | --- | --- | --- |
| Academics/Work/Career | Discrimination | 0.6667 | 1.202 | 79.3 | 0.555 | 1.0000 |
|  | Family/Home | -0.1071 | 0.675 | 84.0 | -0.159 | 1.0000 |
|  | Finances | 0.5000 | 1.112 | 72.2 | 0.450 | 1.0000 |
|  | Mental | 0.7600 | 0.655 | 84.0 | 1.159 | 1.0000 |
|  | Physical | 0.6667 | 0.793 | 83.2 | 0.841 | 1.0000 |
|  | Romantic | 1.3333 | 1.154 | 84.9 | 1.156 | 1.0000 |
|  | Social Situations | -0.5000 | 0.743 | 84.2 | -0.673 | 1.0000 |
| Discrimination | Family/Home | -0.7738 | 1.205 | 46.7 | -0.642 | 1.0000 |
|  | Finances | -0.1667 | 1.280 | 82.4 | -0.130 | 1.0000 |
|  | Mental | 0.0933 | 1.144 | 64.4 | 0.082 | 1.0000 |
|  | Physical | 0.0000 | 1.182 | 82.1 | 0.000 | 1.0000 |
|  | Romantic | 0.6667 | 1.385 | 84.6 | 0.481 | 1.0000 |
|  | Social Situations | -1.1667 | 1.195 | 66.7 | -0.977 | 1.0000 |
| Family & Home Life | Finances | 0.6071 | 1.119 | 31.8 | 0.543 | 1.0000 |
|  | Mental | 0.8671 | 0.483 | 83.8 | 1.794 | 1.0000 |
|  | Physical | 0.7738 | 0.698 | 76.6 | 1.108 | 1.0000 |
|  | Romantic | 1.4405 | 1.122 | 74.3 | 1.284 | 1.0000 |
|  | Social Situations | -0.3929 | 0.603 | 82.7 | -0.651 | 1.0000 |
| Finances | Mental | 0.2600 | 1.050 | 50.1 | 0.248 | 1.0000 |
|  | Physical | 0.1667 | 1.089 | 76.9 | 0.153 | 1.0000 |
|  | Romantic | 0.8333 | 1.298 | 84.5 | 0.642 | 1.0000 |
|  | Social Situations | -1.0000 | 1.106 | 53.9 | -0.904 | 1.0000 |
| Mental Health | Physical | -0.0933 | 0.665 | 84.8 | -0.140 | 1.0000 |
|  | Romantic | 0.5733 | 1.080 | 82.8 | 0.531 | 1.0000 |
|  | Social Situations | -1.2600 | 0.590 | 83.5 | -2.134 | 1.0000 |
| Physical Health | Romantic | 0.6667 | 1.144 | 85.0 | 0.583 | 1.0000 |
|  | Social Situations | -1.1667 | 0.749 | 84.6 | -1.558 | 1.0000 |
| Romantic Relationships | Social Situations | -1.8333 | 1.138 | 82.0 | -1.610 | 1.0000 |

### **Table A.2.5c:** *Results for Hypothesis 1b: Scenario domain and emotion regulation efficacy, Model comparisons*

|  | *npar* | AIC | BIC | Log Likelihood | Deviance | Chi-Square | Df | *P* |
| --- | --- | --- | --- | --- | --- | --- | --- | --- |
| Baseline | 3 | 366.18 | 373.78 | -180.09 | 360.18 |  |  |  |
| Random effects | 10 | 371.54 | 396.86 | -175.77 | 351.54 | 8.6429 | 7 | 0.2793 |

### **Table A.2.6a:** *Results for Hypothesis 1c: Scenario domain and reappraisal efficacy, Model results*

| Random Effects |  |  |  |  |
| --- | --- | --- | --- | --- |
|  | Groups | Name | Variance | SD |
| *Null model* |  |  |  |  |
|  | ParticipantID | Intercept | 1.6829 | 1.2973 |
|  | Residual |  | 0.9934 | 0.9967 |
| *Random intercepts model* |  |  |  |  |
|  | ParticipantID | Intercept | 1.9191 | 1.3853 |
|  | Residual |  | 0.9916 | 0.9958 |

| Fixed Effects |  |  |  |  |
| --- | --- | --- | --- | --- |
|  |  | *B* | SE | *t* |
| *Null model* |  |  |  |  |
|  | Intercept | 3.4818 | 0.5578 | 6.242 |
| *Random intercepts model* |  |  |  |  |
|  | Intercept | 3.0155 | 0.6737 | 4.476 |
|  | Discrimination | 0.1067 | 0.6910 | 0.154 |
|  | Family & Home Life | 0.7344 | 0.3900 | 1.883 |
|  | Finances | -0.1294 | 0.6268 | -0.206 |
|  | Mental Health | 0.3739 | 0.3944 | 0.948 |
|  | Physical Health | 0.7526 | 0.4728 | 1.592 |
|  | Romantic Relationships | 0.4566 | 0.6766 | 0.675 |
|  | Social Situations | 0.7197 | 0.4504 | 1.598 |

### **Table A.2.6b:** *Results for Hypothesis 1c: Scenario domain and reappraisal efficacy, Pairwise comparisons*

| Domain |  | *B* | SE | df | *t* | *P* |
| --- | --- | --- | --- | --- | --- | --- |
| Academics/Work/Career | Discrimination | -0.1067 | 0.693 | 80.8 | -0.154 | 1.0000 |
|  | Family/Home | -0.7344 | 0.390 | 80.3 | -1.882 | 1.0000 |
|  | Finances | 0.1294 | 0.628 | 80.8 | 0.206 | 1.0000 |
|  | Mental | -0.3739 | 0.395 | 80.8 | -0.946 | 1.0000 |
|  | Physical | -0.7526 | 0.473 | 80.2 | -1.591 | 1.0000 |
|  | Romantic | -0.4566 | 0.677 | 80.3 | -0.674 | 1.0000 |
|  | Social Situations | -0.7197 | 0.452 | 81.2 | -1.593 | 1.0000 |
| Discrimination | Family/Home | -0.6277 | 0.658 | 81.3 | -0.955 | 1.0000 |
|  | Finances | 0.2361 | 0.762 | 80.1 | 0.310 | 1.0000 |
|  | Mental | -0.2672 | 0.660 | 81.8 | -0.405 | 1.0000 |
|  | Physical | -0.6459 | 0.693 | 81.0 | -0.932 | 1.0000 |
|  | Romantic | -0.3500 | 0.830 | 80.6 | -0.421 | 1.0000 |
|  | Social Situations | -0.6131 | 0.701 | 82.1 | -0.875 | 1.0000 |
| Family & Home Life | Finances | 0.8637 | 0.588 | 81.3 | 1.470 | 1.0000 |
|  | Mental | 0.3605 | 0.283 | 80.8 | 1.272 | 1.0000 |
|  | Physical | -0.0182 | 0.397 | 80.5 | -0.046 | 1.0000 |
|  | Romantic | 0.2777 | 0.627 | 80.3 | 0.443 | 1.0000 |
|  | Social Situations | 0.0146 | 0.362 | 81.3 | 0.040 | 1.0000 |
| Finances | Mental | -0.5033 | 0.589 | 81.8 | -0.855 | 1.0000 |
|  | Physical | -0.8820 | 0.629 | 81.0 | -1.402 | 1.0000 |
|  | Romantic | -0.5860 | 0.773 | 80.4 | -0.758 | 1.0000 |
|  | Social Situations | -0.8491 | 0.635 | 82.1 | -1.337 | 1.0000 |
| Mental Health | Physical | -0.3787 | 0.394 | 80.6 | -0.961 | 1.0000 |
|  | Romantic | -0.0827 | 0.628 | 80.5 | -0.132 | 1.0000 |
|  | Social Situations | -0.3459 | 0.354 | 80.4 | -0.978 | 1.0000 |
| Physical Health | Romantic | 0.2959 | 0.679 | 80.4 | 0.436 | 1.0000 |
|  | Social Situations | 0.0328 | 0.450 | 81.0 | 0.073 | 1.0000 |
| Romantic Relationships | Social Situations | -0.2631 | 0.671 | 80.9 | -0.392 | 1.0000 |

### **Table A.2.6c:** *Results for Hypothesis 1c: Scenario domain and reappraisal efficacy, Model comparisons*

|  | *npar* | AIC | BIC | Log Likelihood | Deviance | Chi-Square | Df | *P* |
| --- | --- | --- | --- | --- | --- | --- | --- | --- |
| Baseline | 3 | 284.48 | 292.08 | -139.24 | 278.48 |  |  |  |
| Random effects | 10 | 291.74 | 317.06 | -135.87 | 271.74 | 6.7444 | 7 | 0.456 |

### **Table A.2.7a:** *Results for Hypotheses 2a and 2b: Writing demand and affect score, Model results*

| Random Effects |  |  |  |  |
| --- | --- | --- | --- | --- |
|  | Groups | Name | Variance | SD |
| *Null model* |  |  |  |  |
|  | ParticipantID | Intercept | 0.2791 | 0.5283 |
|  | Residual |  | 0.6307 | 0.7942 |
| *Random intercepts model* |  |  |  |  |
|  | ParticipantID | Intercept | 0.1859 | 0.4311 |
|  | Residual |  | 0.5446 | 0.7380 |

| Fixed Effects |  |  |  |  |
| --- | --- | --- | --- | --- |
|  |  | *B* | SE | *t* |
| *Null model* |  |  |  |  |
|  | Intercept | 1.6552 | 0.5950 | 2.782 |
|  | PreEMA | 0.6390 | 0.1253 | 5.098 |
| *Random intercepts model* |  |  |  |  |
|  | Intercept | 1.28682 | 0.65326 | 1.970 |
|  | PreEMA | 0.71439 | 0.12121 | 5.894 |
|  | Long | 0.18581 | 0.43541 | 0.427 |
|  | One-letter | 0.09844 | 0.30275 | 0.325 |
|  | Two-letter | -0.39679 | 0.32853 | -1.208 |
|  | Write your own | -1.32858 | 0.42082 | -3.157 |

### **Table A.2.7b:** *Results for Hypotheses 2a and 2b: Writing demand and affect score, Pairwise comparisons with contrasts*

| Contrast | *B* | SE | df | *t* | *P* |
| --- | --- | --- | --- | --- | --- |
| No writing - Fill-in-the-blank | -0.149 | 0.294 | 76.0 | -0.508 | 0.6129 |
| No writing - More writing | 0.422 | 0.268 | 75.5 | 1.574 | 0.1196 |
| Fill-in-the-blank - More writing | 0.571 | 0.354 | 74.4 | 1.614 | 0.1107 |

### **Table A.2.7c:** *Results for Hypotheses 2a and 2b: Writing demand and affect score, Model comparison*

|  | *npar* | AIC | BIC | Log Likelihood | Deviance | Chi-Square | Df | *P* |
| --- | --- | --- | --- | --- | --- | --- | --- | --- |
| Baseline | 4 | 217.21 | 226.98 | -104.603 | 209.21 |  |  |  |
| Random effects | 8 | 207.46 | 227.00 | -95.731 | 191.46 | 17.743 | 4 | 0.001 |

### **Table A.2.8a:** *Results for Hypotheses 2a and 2b: Writing demand and emotion regulation efficacy, Model results*

| Random Effects |  |  |  |  |
| --- | --- | --- | --- | --- |
|  | Groups | Name | Variance | SD |
| *Null model* |  |  |  |  |
|  | ParticipantID | Intercept | 0.000 | 0.000 |
|  | Residual |  | 2.739 | 1.655 |
| *Random intercepts model* |  |  |  |  |
|  | ParticipantID | Intercept | < 0.001 | < 0.001 |
|  | Residual |  | 2.739 | 1.655 |

| Fixed Effects |  |  |  |  |
| --- | --- | --- | --- | --- |
|  |  | *B* | SE | *t* |
| *Null model* |  |  |  |  |
|  | Intercept | 4.6941 | 0.1795 | 26.15 |
| *Random intercepts model* |  |  |  |  |
|  | Intercept | 4.5000 | 0.5851 | 7.691 |
|  | Long | 0.7000 | 0.9435 | 0.742 |
|  | One-letter | 0.1981 | 0.6277 | 0.316 |
|  | Two-letter | -0.2857 | 0.7335 | -0.390 |
|  | Write your own | 1.3000 | 0.9435 | 1.378 |

### **Table A.2.8b:** *Results for Hypotheses 2a and 2b: Writing demand and emotion regulation efficacy, Pairwise comparisons with grouping by contrasts*

| Contrast | *B* | SE | df | *t* | *P* |
| --- | --- | --- | --- | --- | --- |
| No writing - Fill-in-the-blank | -0.0438 | 0.685 | 76.4 | -0.064 | 0.9492 |
| No writing - More writing | -1.0438 | 0.633 | 73.4 | -1.650 | 0.1032 |
| Fill-in-the-blank - More writing | -1.0000 | 0.785 | 77.0 | -1.274 | 0.2066 |

### **Table A.2.8c:** *Results for Hypotheses 2a and 2b: Writing demand and emotion regulation efficacy, Model comparison*

|  | *npar* | AIC | BIC | Log Likelihood | Deviance | Chi-Square | Df | *P* |
| --- | --- | --- | --- | --- | --- | --- | --- | --- |
| Baseline | 3 | 331.85 | 339.18 | -162.92 | 325.85 |  |  |  |
| Random effects | 7 | 335.71 | 352.81 | -160.86 | 321.71 | 4.1338 | 4 | 0.3882 |

### **Table A.2.9a:** *Results for Hypotheses 2a and 2b: Writing demand and reappraisal efficacy, Model results*

| Random Effects |  |  |  |  |
| --- | --- | --- | --- | --- |
|  | Groups | Name | Variance | SD |
| *Null model* |  |  |  |  |
|  | ParticipantID | Intercept | 1.716 | 1.310 |
|  | Residual |  | 1.023 | 1.012 |
| *Random intercepts model* |  |  |  |  |
|  | ParticipantID | Intercept | 1.764 | 1.328 |
|  | Residual |  | 1.057 | 1.028 |

| Fixed Effects |  |  |  |  |
| --- | --- | --- | --- | --- |
|  |  | *B* | SE | *t* |
| *Null model* |  |  |  |  |
|  | Intercept | 3.5002 | 0.5651 | 6.194 |
| *Random intercepts model* |  |  |  |  |
|  | Intercept | 3.9265 | 0.7003 | 5.607 |
|  | Long | -0.4750 | 0.5862 | -0.810 |
|  | One-letter | -0.4469 | 0.4236 | -1.055 |
|  | Two-letter | -0.2873 | 0.4563 | -0.630 |
|  | Write your own | -0.4750 | 0.5862 | -0.810 |

### **Table A.2.9b:** *Results for Hypotheses 2a and 2b: Writing demand and reappraisal efficacy, Pairwise comparisons with grouping by contrasts*

| Contrast | *B* | SE | df | *t* | *P* |
| --- | --- | --- | --- | --- | --- |
| No writing - Fill-in-the-blank | -0.367 | 0.406 | 75.3 | -0.904 | 0.3688 |
| No writing - More writing | 0.108 | 0.372 | 75.4 | 0.290 | 0.7726 |
| Fill-in-the-blank - More writing | 0.475 | 0.488 | 75.0 | 0.974 | 0.3332 |

### **Table 2.9c:** *Results for Hypotheses 2a and 2b: Writing demand and reappraisal efficacy, Model comparison*

|  | *npar* | AIC | BIC | Log Likelihood | Deviance | Chi-Square | Df | *P* |
| --- | --- | --- | --- | --- | --- | --- | --- | --- |
| Baseline | 3 | 263.92 | 271.24 | -128.96 | 257.92 |  |  |  |
| Random effects | 7 | 270.55 | 287.64 | -128.27 | 256.55 | 1.3705 | 4 | 0.8493 |

### **Table A.2.10a:** *Results for Hypothesis 3a: Post-CBM-I recommendation and affect score, Model results*

| Random Effects |  |  |  |  |
| --- | --- | --- | --- | --- |
|  | Groups | Name | Variance | SD |
| *Null model* |  |  |  |  |
|  | ParticipantID | Intercept | 0.2915 | 0.5399 |
|  | Residual |  | 0.5943 | 0.7709 |
| *Random intercepts model* |  |  |  |  |
|  | ParticipantID | Intercept | 0.2938 | 0.5421 |
|  | Residual |  | 0.5996 | 0.7743 |

| Fixed Effects |  |  |  |  |
| --- | --- | --- | --- | --- |
|  |  | *B* | SE | *t* |
| *Null model* |  |  |  |  |
|  | Intercept | 1.7310 | 0.5430 | 3.188 |
|  | PreEMA | 0.6226 | 0.1119 | 5.566 |
| *Random intercepts model* |  |  |  |  |
|  | Intercept | 1.522475 | 0.576695 | 2.640 |
|  | PreEMA | 0.657733 | 0.117485 | 5.598 |
|  | Resource | 0.218020 | 0.211666 | 1.030 |
|  | Tip | 0.005981 | 0.204989 | 0.029 |

### **Table A.2.10b:** *Results for Hypothesis 3a: Post-CBM-I recommendation and affect score, Pairwise comparisons*

| Recommendation | *B* | SE | df | *t* | *P* |
| --- | --- | --- | --- | --- | --- |
| ER - Resource | -0.21802 | 0.214 | 81.4 | -1.021 | 0.9313 |
| ER - Tip | -0.00598 | 0.206 | 79.4 | -0.029 | 0.9769 |
| Resource - Tip | 0.21204 | 0.225 | 79.2 | 0.941 | 0.9313 |

### **Table A.2.10c:** *Results for Hypothesis 3a: Post-CBM-I recommendation and affect score, Model comparison*

|  | *npar* | AIC | BIC | Log Likelihood | Deviance | Chi-Square | Df | *P* |
| --- | --- | --- | --- | --- | --- | --- | --- | --- |
| Baseline | 4 | 217.28 | 227.14 | -104.64 | 209.28 |  |  |  |
| Random effects | 6 | 219.99 | 234.79 | -104.00 | 207.99 | 1.285 | 2 | 0.526 |

### **Table A.2.11a:** *Results for Hypothesis 3a: Post-CBM-I recommendation and emotion regulation efficacy, Model results*

| Random Effects |  |  |  |  |
| --- | --- | --- | --- | --- |
|  | Groups | Name | Variance | SD |
| *Null model* |  |  |  |  |
|  | ParticipantID | Intercept | 0.000 | 0.000 |
|  | Residual |  | 2.821 | 1.68 |
| *Random intercepts model* |  |  |  |  |
|  | ParticipantID | Intercept | 0.000 | 0.000 |
|  | Residual |  | 2.883 | 1.698 |

| Fixed Effects |  |  |  |  |
| --- | --- | --- | --- | --- |
|  |  | *B* | SE | *t* |
| *Null model* |  |  |  |  |
|  | Intercept | 4.6897 | 0.1801 | 26.04 |
| *Random intercepts model* |  |  |  |  |
|  | Intercept | 4.70588 | 0.29121 | 16.160 |
|  | Resource | -0.11329 | 0.43771 | -0.259 |
|  | Tip | 0.06335 | 0.44237 | 0.143 |

### **Table A.2.11b:** *Results for Hypothesis 3a: Post-CBM-I recommendation and emotion regulation efficacy, Pairwise comparisons*

| Recommendation | *B* | SE | df | *t* | *P* |
| --- | --- | --- | --- | --- | --- |
| ER - Resource | 0.1133 | 0.461 | 81.3 | 0.246 | 1.0000 |
| ER - Tip | -0.0633 | 0.447 | 83.0 | -0.142 | 1.0000 |
| Resource - Tip | -0.1766 | 0.474 | 83.6 | -0.373 | 1.0000 |

### **Table A.2.11c:** *Results for Hypothesis 3a: Post-CBM-I recommendation and emotion regulation efficacy, Model comparison*

|  | *npar* | AIC | BIC | Log Likelihood | Deviance | Chi-Square | Df | *P* |
| --- | --- | --- | --- | --- | --- | --- | --- | --- |
| Baseline | 3 | 342.12 | 349.52 | -168.06 | 336.12 |  |  |  |
| Random effects | 5 | 345.97 | 358.30 | -167.98 | 335.97 | 0.1536 | 2 | 0.9261 |

### **Table A.2.12a:** *Results for Hypothesis 3b: Post-CBM-I recommendation and reappraisal efficacy, Model results*

| Random Effects |  |  |  |  |
| --- | --- | --- | --- | --- |
|  | Groups | Name | Variance | SD |
| *Null model* |  |  |  |  |
|  | ParticipantID | Intercept | 1.7959 | 1.3401 |
|  | Residual |  | 0.7803 | 0.8834 |
| *Random intercepts model* |  |  |  |  |
|  | ParticipantID | Intercept | 1.8028 | 1.343 |
|  | Residual |  | 0.7499 | 0.866 |

| Fixed Effects |  |  |  |  |
| --- | --- | --- | --- | --- |
|  |  | *B* | SE | *t* |
| *Null model* |  |  |  |  |
|  | Intercept | 3.4839 | 0.5701 | 6.111 |
| *Random intercepts model* |  |  |  |  |
|  | Intercept | 3.36863 | 0.58176 | 5.790 |
|  | Resource | -0.07836 | 0.23446 | -0.334 |
|  | Tip | 0.42739 | 0.22865 | 1.869 |

### **Table A.2.12b:** *Results for Hypothesis 3b: Post-CBM-I recommendation and reappraisal efficacy, Pairwise comparisons*

| Recommendation | *B* | SE | df | *t* | *P* |
| --- | --- | --- | --- | --- | --- |
| ER - Resource | 0.0784 | 0.235 | 80.2 | 0.333 | 0.7399 |
| ER - Tip | -0.4274 | 0.229 | 79.5 | -1.867 | 0.1312 |
| Resource - Tip | -0.5057 | 0.241 | 79.3 | -2.094 | 0.1182 |

### **Table A.2.12c:** *Results for Hypothesis 3b: Post-CBM-I recommendation and reappraisal efficacy, Model comparison*

|  | *npar* | AIC | BIC | Log Likelihood | Deviance | Chi-Square | Df | *P* |
| --- | --- | --- | --- | --- | --- | --- | --- | --- |
| Baseline | 3 | 247.81 | 255.21 | -120.91 | 241.81 |  |  |  |
| Random effects | 5 | 246.57 | 258.90 | -118.28 | 236.57 | 5.2429 | 2 | 0.0727 |

# Faculty Descriptives & Analyses including all pre-microdose affect scores (1-7)

## **Results**

**Demographics**

Nine faculty member participants were included in our sample. All nine participants identified as White/European (100%) and non-Hispanic (100%), and a majority of the participants identified as women (66.67%). Participant ages ranged from 35 to 67 (*M* = 52.00, *SD* = 10.69). See Table A.3.1 for full demographic information.

**Microdose Characteristics**

Across the full sample, participants completed 178 microdoses in total. Microdoses missing either pre- or post-microdose affect, cognitive reappraisal, or emotion regulation efficacy scores (as a result of either participants choosing to skip the question in the app or technical issues in the data being recorded) were removed (*n* = 7), leaving 171 microdoses completed across 9 participants. The total number of microdoses completed by participants during their study participation ranged from one (2.4% of total expected) to 68 (over 100% of total expected) microdoses (*M* = 21.4, *SD* = 25.5). Due to a technical error with how the mobile application collected data at the beginning of the study (October 2022 - January 2023), scenario format and recommendation data are missing for a portion of the microdoses. Specifically, the microdose domain is known for all but one microdose (99.42%), but scenario format is known for 151 microdoses (88.3%) and recommendation type is known for 157 microdoses (91.81%). On average, participants rated their affect as being somewhat positive both immediately prior to (5.23 out of 7) and immediately after (4.90 out of 7) completing a microdose; participants rated their cognitive reappraisal efficacy (3.32 out of 7) as being fairly neutral (as opposed to better or worse) after completing a microdose; participants rated their their emotion regulation efficacy (5.39 out of 7) as being somewhat better immediately after completing a microdose. Family and Home Life was the most frequently chosen domain (*n =* 35); the least chosen domains were Discrimination (*n =* 8) and Romantic Relationships (*n =* 11). See Table A.3.2 for full descriptives.

For each model, the total variance explained by the fixed effects and the total variance explained by both fixed and random effects, as well as the intraclass correlation coefficient (ICC), is reported in Table A.3.3.

**How did post-microdose affect scores (controlling for pre-microdose affect) differ based on domain? (Hypothesis 1a)**

There was no significant difference in post-microdose affect scores across stressor domains. See Tables A.3.4a and A.3.4b for model results and pairwise comparisons. The random intercepts model with domain as a predictor did not perform significantly better than the null model with no predictor (Chi-square=14.011, df=2, *P*=0.05099). See Table A.3.4c for full model comparison, including Akaike Information Criterion (AIC) values.

**How did post-microdose emotion regulation efficacy differ based on domain? (Hypothesis 1b)**

There was no significant difference in emotion regulation efficacy scores across stressor domains. See Tables A.3.5a and A.3.5b for model results and pairwise comparisons, respectively. The random intercepts model with domain as a predictor performed significantly better than the null model with no predictor (Chi-square=16.291, df=7, *P*=0.02259). Additionally, we ran a random intercepts model with pre-microdose affect score as a predictor along with domain to control for the effect of affect on emotion regulation efficacy. The random intercepts model controlling for pre-microdose affect performed significantly better than the random intercepts model without controlling for pre-microdose affect (Chi-square=42.29, df=1, *P<*.001). See Table A.3.5c for full model comparisons.

**How did post-microdose reappraisal efficacy differ based on domain? (Hypothesis 1c)**

Reappraisal scores were significantly better after microdoses completed in the Finances domain compared to all other domains; see Tables A.3.6a and A.3.6b for model results and pairwise comparisons. The random intercepts model with domain as a predictor performed significantly better than the null model with no predictor (Chi-square=19.637, df=7, *P*=.006). Additionally, we ran a random intercepts model with pre-microdose affect score as a predictor along with domain to control for the effect of affect on reappraisal; the random intercepts model controlling for pre-microdose affect performed significantly better than the random intercepts model without controlling for pre-microdose affect (Chi-square=16.719, df=1, *P<*.001). See Table A.3.6c for full model comparisons.

**How did post-microdose affect scores (controlling for pre-microdose affect) differ based on writing demand? (Hypotheses 2a and 2b)**

Writing demand data was collected for 8 (out of 9) participants, including 151 (out of 178) microdoses. Analyses were conducted only on the subset of microdoses that contain scenario format data.

Both scenarios with no writing (one- and two-letters missing; *B=*0.5881, *SE=*0.195, *P=*0.0030) and some writing (fill-in-the-blank scenarios; *B=*0.6495, *SE=*0.240, *P=*0.0077) were associated with significantly more positive post-microdose affect than scenarios containing a large amount of writing (write your own and long scenarios). There was no significant difference between post-microdose affect scores for scenarios with no writing (one- and two-letters missing) and fill-in-the-blank scenarios (*B=*-0.0614, *SE=*0.193, *P=*0.7508). See Tables A.3.7a and A.3.7b for model results and pairwise comparisons. The random intercepts model with writing demand as a predictor performed significantly better than the null model with no predictor (Chi-square=36.746, df=4, *P*<0.001). See Table A.3.7c for full model comparison.

**How did post-microdose emotion regulation efficacy differ based on writing demand? (Hypotheses 2a and 2b)**

Scenarios with no writing (one- and two-letters missing) were associated with significantly more negative emotion regulation efficacy scores than scenarios containing a large amount of writing (write your own and long scenarios; *B=*-0.769, *SE=*0.374, *P=*0.0414). There was no significant difference between emotion regulation efficacy scores for scenarios with no writing (one- and two-letters missing) and fill-in-the-blank scenarios (*B=*-0.159, *SE=*0.371, *P=*0.6677), or fill-in-the-blank scenarios and scenarios containing a large amount of writing (write your own and long scenarios; *B=*-0.610, *SE=*0.465, *P=*0.1919). See Tables A.3.8a and A.3.8b for model results and pairwise comparisons. The random intercepts model with writing demand as a predictor did not perform significantly better than the null model with no predictor (Chi-square=4.6462, df=4, *P*=0.3256). See Table A.3.8c for full model comparison.

**How did post-microdose reappraisal efficacy differ based on writing demand? (Hypotheses 2a and 2b)**

There was no significant difference in reappraisal efficacy scores across scenario formats. See Tables A.3.9a and 3 A..9b for model results and pairwise comparisons. The random intercepts model with writing demand as a predictor did not perform significantly better than the null model with no predictor (Chi-square=2.5166, df=4, *P*=0.6417). See Table A.3.9c for full model comparison.

**How did post-microdose affect scores (controlling for pre-microdose affect) differ based on post-CBM-I recommendation? (Hypothesis 3a)**

Post-CBM-I recommendation data was collected for 8 (out of 9) participants, including 157 (out of 178) microdoses. Analyses were conducted only on the subset of microdoses that contain post-CBM-I recommendation data.

There was no significant difference between post-microdose affect scores across post-CBM-I recommendation types. See Tables A.3.10a and A.3.10b for model results and pairwise comparisons. The random intercepts model with post-CBM-I recommendation as a predictor did not perform significantly better than the null model with no predictor (Chi-square=1.3588, df=2, *P*=0.5069). See Table A.3.10c for full model comparison.

**How did post-microdose emotion regulation efficacy scores differ based on post-CBM-I recommendation? (Hypothesis 3a)**

There was no significant difference between post-microdose emotion regulation efficacy scores across post-CBM-I recommendation types. See Tables A.3.11a and A.3.11b for model results and pairwise comparisons. The random intercepts model with post-CBM-I recommendation as a predictor did not perform significantly better than the null model with no predictor (Chi-square=1.0231, df=2, *P*=0.5996). See Table A.3.11c for full model comparison.

**How did post-microdose reappraisal efficacy scores differ based on post-CBM-I recommendation? (Hypothesis 3b)**

There was no significant difference in reappraisal efficacy scores across post-CBM-I recommendation types. See Tables A.3.12a and A.3.12b for model results and pairwise comparisons. The random intercepts model with post-CBM-I recommendation types as a predictor did not perform significantly better than the null model with no predictor (Chi-square=3.3386, df=2, *P*=0.1884). See Table A.3.12c for full model comparison.

## **Tables**

| **Table A.3.1:** *Demographic Characteristics* |  |
| --- | --- |
| Characteristic | *n* (%) |
| Gender:  Man  Woman  Transgender Man  Transgender Woman  Other identity  Not Reported  Race:  White/European Origin  East Asian  South Asian  Other or Unknown  Black/African Origin  Participant selected more than one race  American Indian/Alaska Native  Native Hawaiian/Pacific Islander  Ethnicity:  Not Hispanic or Latino  Hispanic or Latino | 2 (22.22%)  6 (66.67%)  0 (0%)  0 (0%)  0 (0%)  1 (1.03%)  9 (100%)  0 (0%)  0 (0%)  0 (0%)  0 (0%)  0 (0%)  0 (0%)  0 (0%)  9 (100%)  0 (0%) |

### **Table A.3.2:** *Descriptive Statistics*

|  | *n* | Mean | SD | Range |
| --- | --- | --- | --- | --- |
| Microdoses per Domain*  Academics/Work/Career Development  Discrimination  Family & Home Life  Finances  Mental Health  Physical Health  Romantic Relationships  Social Situations  Not available | 27  8  35  14  28  24  11  23  1 | 19 | 11.04 | 27 |
| Microdoses per Scenario Type*  Fill-in-the-blank  Long scenario  One-letter  Two-letter  Write-your-own  Not available | 19  10  84  29  9  20 | 28.5 | 28.16 | 75 |
| Microdoses per Recommendation Type*  Emotion Regulation  Resource  Tip  Not available | 63  48  46  14 | 42.75 | 20.61 | 49 |
| Ecological Momentary Assessment Scores  Pre-Microdose EMA [out of 7]  Post-Microdose EMA [out of 7]  Cognitive Reappraisal Efficacy [out of 7]  Emotion Regulation Efficacy [out of 7]  *Mean, SD, and range based off of microdoses that have the data available |  | 5.23  4.90  3.32  5.39 | 1.51  1.54  1.67  1.55 | 6  6  6  6 |

### **Table A.3.3:** *Percent of total variance explained by fixed and random effects and intraclass correlation (ICC) for all models*

|  |  | Fixed effects | Fixed and random effects | ICC |
| --- | --- | --- | --- | --- |
| Test | Model |  |  |  |
| Scenario domain and affect score | Null | 47.44% | 64.39% | 0.323 |
|  | Random Intercepts | 48.08% | 65.89% | 0.343 |
| Scenario domain and ER efficacy | Null | 0.00% | 17.46% | 0.175 |
|  | Random Intercepts | 7.51% | 23.97% | 0.178 |
|  | Random Intercepts controlling for affect | 34.09% | 34.31% | 0.003 |
| Scenario domain and reappraisal efficacy | Null | 0.00% | 45.58% | 0.456 |
|  | Random Intercepts | 6.57% | 48.16% | 0.445 |
|  | Random Intercepts controlling for affect | 17.24% | 59.84% | 0.515 |
| Writing demand and affect score | Null | 41.70% | 62.06% | 0.349 |
|  | Random Intercepts | 56.58% | 70.94% | 0.331 |
| Writing demand and ER efficacy | Null | 0.00% | 18.43% | 0.184 |
|  | Random Intercepts | 2.75% | 21.82% | 0.196 |
| Writing demand and reappraisal efficacy | Null | 0.00% | 47.88% | 0.479 |
|  | Random Intercepts | 0.89% | 47.37% | 0.469 |
| Recommendation and affect score | Null | 56.90% | 70.27% | 0.31 |
|  | Random Intercepts | 56.31% | 70.19% | 0.318 |
| Recommendation and ER efficacy | Null | 0.00% | 18.66% | 0.187 |
|  | Random Intercepts | 0.55% | 20.11% | 0.197 |
| Recommendation and reappraisal efficacy | Null | 0.00% | 46.88% | 0.469 |
|  | Random Intercepts | 1.13% | 48.45% | 0.479 |

### **Table A.3.4a:** *Results for Hypothesis 1a: Scenario domain and affect score, Model results*

| Random Effects |  |  |  |  |
| --- | --- | --- | --- | --- |
|  | Groups | Name | Variance | SD |
| *Null model* |  |  |  |  |
|  | ParticipantID | Intercept | 0.3183 | 0.5642 |
|  | Residual |  | 0.6687 | 0.8177 |
| *Random intercepts model* |  |  |  |  |
|  | ParticipantID | Intercept | 0.3343 | 0.5782 |
|  | Residual |  | 0.6403 | 0.8002 |
|  |  |  |  |  |

| Fixed Effects |  |  |  |  |
| --- | --- | --- | --- | --- |
|  |  | *B* | SE | *t* |
| *Null model* |  |  |  |  |
|  | Intercept | 1.81975 | 0.39789 | 4.573 |
|  | PreEMA | 0.62409 | 0.06258 | 9.972 |
| *Random intercepts model* |  |  |  |  |
|  | Intercept | 2.48834 | 0.46171 | 5.389 |
|  | PreEMA | 0.56771 | 0.06462 | 8.785 |
|  | Discrimination | -0.06160 | 0.32748 | -0.188 |
|  | Family & Home Life | -0.52984 | 0.21909 | -2.418 |
|  | Finances | -0.70844 | 0.27372 | -2.588 |
|  | Mental Health | -0.59528 | 0.23066 | -2.581 |
|  | Physical Health | -0.35665 | 0.23115 | -1.543 |
|  | Romantic Relationships | -0.46622 | 0.29676 | -1.571 |
|  | Social Situations | -0.13190 | 0.23055 | -0.572 |

### **Table A.3.4b:** *Results for Hypothesis 1a: Scenario domain and affect score, Pairwise comparisons*

| Domain |  | *B* | SE | df | *t* | *P* |
| --- | --- | --- | --- | --- | --- | --- |
| Academics/Work/Career | Discrimination | 0.0616 | 0.328 | 156 | 0.188 | 1.0000 |
|  | Family/Home | 0.5298 | 0.219 | 156 | 2.415 | 0.4397 |
|  | Finances | 0.7084 | 0.275 | 157 | 2.581 | 0.3018 |
|  | Mental | 0.5953 | 0.231 | 156 | 2.577 | 0.3018 |
|  | Physical | 0.3566 | 0.232 | 157 | 1.539 | 1.0000 |
|  | Romantic | 0.4662 | 0.298 | 160 | 1.563 | 1.0000 |
|  | Social Situations | 0.1319 | 0.231 | 156 | 0.571 | 1.0000 |
| Discrimination | Family/Home | 0.4682 | 0.331 | 156 | 1.414 | 1.0000 |
|  | Finances | 0.6468 | 0.357 | 155 | 1.813 | 1.0000 |
|  | Mental | 0.5337 | 0.336 | 156 | 1.589 | 1.0000 |
|  | Physical | 0.2951 | 0.330 | 155 | 0.894 | 1.0000 |
|  | Romantic | 0.4046 | 0.379 | 158 | 1.069 | 1.0000 |
|  | Social Situations | 0.0703 | 0.334 | 156 | 0.210 | 1.0000 |
| Family & Home Life | Finances | 0.1786 | 0.281 | 157 | 0.636 | 1.0000 |
|  | Mental | 0.0654 | 0.208 | 156 | 0.314 | 1.0000 |
|  | Physical | -0.1732 | 0.233 | 157 | -0.743 | 1.0000 |
|  | Romantic | -0.0636 | 0.300 | 160 | -0.212 | 1.0000 |
|  | Social Situations | -0.3979 | 0.229 | 157 | -1.736 | 1.0000 |
| Finances | Mental | -0.1132 | 0.284 | 157 | -0.398 | 1.0000 |
|  | Physical | -0.3518 | 0.272 | 155 | -1.292 | 1.0000 |
|  | Romantic | -0.2422 | 0.330 | 159 | -0.734 | 1.0000 |
|  | Social Situations | -0.5765 | 0.279 | 156 | -2.069 | 1.0000 |
| Mental Health | Physical | -0.2386 | 0.237 | 155 | -1.007 | 1.0000 |
|  | Romantic | -0.1291 | 0.303 | 159 | -0.425 | 1.0000 |
|  | Social Situations | -0.4634 | 0.234 | 155 | -1.976 | 1.0000 |
| Physical Health | Romantic | 0.1096 | 0.299 | 159 | 0.366 | 1.0000 |
|  | Social Situations | -0.2247 | 0.235 | 155 | -0.955 | 1.0000 |
| Romantic Relationships | Social Situations | -0.3343 | 0.303 | 160 | -1.104 | 1.0000 |

### **Table A.3.4c:** *Results for Hypothesis 1a: Scenario domain and affect score, Model comparison*

|  | *npar* | AIC | BIC | Log Likelihood | Deviance | Chi-Square | Df | *P* |
| --- | --- | --- | --- | --- | --- | --- | --- | --- |
| Baseline | 4 | 435.21 | 447.75 | -213.6 | 427.21 |  |  |  |
| Random effects | 11 | 435.20 | 469.69 | -206.6 | 413.20 | 14.011 | 7 | 0.05099 |

### **Table A.3.5a:** *Results for Hypothesis 1b: Scenario domain and emotion regulation efficacy, Model results*

| Random Effects |  |  |  |  |
| --- | --- | --- | --- | --- |
|  | Groups | Name | Variance | SD |
| *Null model* |  |  |  |  |
|  | ParticipantID | Intercept | 0.4419 | 0.6648 |
|  | Residual |  | 2.0886 | 1.4452 |
| *Random intercepts model* |  |  |  |  |
|  | ParticipantID | Intercept | 0.4281 | 0.6543 |
|  | Residual |  | 1.9781 | 1.4065 |
| *Random intercepts model controlling for affect* |  |  |  |  |
|  | ParticipantID | Intercept | 0.005452 | 0.07384 |
|  | Residual |  | 1.650108 | 1.28457 |

| Fixed Effects |  |  |  |  |
| --- | --- | --- | --- | --- |
|  |  | *B* | SE | *t* |
| *Null model* |  |  |  |  |
|  | Intercept | 5.4462 | 0.2921 | 18.65 |
| *Random intercepts model* |  |  |  |  |
|  | Intercept | 5.87604 | 0.38453 | 15.281 |
|  | Discrimination | -0.07359 | 0.57399 | -0.128 |
|  | Family & Home Life | -0.06058 | 0.37460 | -0.162 |
|  | Finances | -0.11353 | 0.47716 | -0.238 |
|  | Mental Health | -1.14544 | 0.38688 | -2.961 |
|  | Physical Health | -0.75554 | 0.40025 | -1.888 |
|  | Romantic Relationships | -0.76486 | 0.51591 | -1.483 |
|  | Social Situations | -0.05753 | 0.40054 | -0.144 |
| *Random intercepts model controlling for affect* |  |  |  |  |
|  | Intercept | 2.431659 | 0.511808 | 4.751 |
|  | PreEMA | 0.578715 | 0.076478 | 7.567 |
|  | Discrimination | -0.066603 | 0.517763 | -0.129 |
|  | Family & Home Life | 0.442648 | 0.346648 | 1.277 |
|  | Finances | 0.001611 | 0.424706 | 0.004 |
|  | Mental Health | -0.481096 | 0.367346 | -1.310 |
|  | Physical Health | -0.474432 | 0.361312 | -1.313 |
|  | Romantic Relationships | -0.608223 | 0.460269 | -1.321 |
|  | Social Situations | 0.247573 | 0.365624 | 0.677 |

### **Table A.3.5b:** *Results for Hypothesis 1b: Scenario domain and emotion regulation efficacy, Pairwise*

### comparisons

| Domain |  | *B* | SE | df | *t* | *P* |
| --- | --- | --- | --- | --- | --- | --- |
| Academics/Work/Career | Discrimination | 0.07359 | 0.575 | 158 | 0.128 | 1.0000 |
|  | Family/Home | 0.06058 | 0.376 | 159 | 0.161 | 1.0000 |
|  | Finances | 0.11353 | 0.479 | 159 | 0.237 | 1.0000 |
|  | Mental | 1.14544 | 0.388 | 159 | 2.951 | 0.0985 |
|  | Physical | 0.75554 | 0.401 | 158 | 1.884 | 1.0000 |
|  | Romantic | 0.76486 | 0.519 | 161 | 1.475 | 1.0000 |
|  | Social Situations | 0.05753 | 0.401 | 157 | 0.144 | 1.0000 |
| Discrimination | Family/Home | -0.01301 | 0.579 | 159 | -0.022 | 1.0000 |
|  | Finances | 0.03994 | 0.626 | 157 | 0.064 | 1.0000 |
|  | Mental | 1.07185 | 0.583 | 159 | 1.839 | 1.0000 |
|  | Physical | 0.68195 | 0.578 | 157 | 1.179 | 1.0000 |
|  | Romantic | 0.69127 | 0.662 | 160 | 1.044 | 1.0000 |
|  | Social Situations | -0.01606 | 0.586 | 158 | -0.027 | 1.0000 |
| Family & Home Life | Finances | 0.05295 | 0.494 | 161 | 0.107 | 1.0000 |
|  | Mental | 1.08486 | 0.364 | 159 | 2.982 | 0.0928 |
|  | Physical | 0.69496 | 0.410 | 161 | 1.697 | 1.0000 |
|  | Romantic | 0.70428 | 0.522 | 162 | 1.349 | 1.0000 |
|  | Social Situations | -0.00305 | 0.402 | 160 | -0.008 | 1.0000 |
| Finances | Mental | 1.03191 | 0.495 | 161 | 2.083 | 0.9715 |
|  | Physical | 0.64201 | 0.478 | 157 | 1.343 | 1.0000 |
|  | Romantic | 0.65133 | 0.577 | 160 | 1.130 | 1.0000 |
|  | Social Situations | -0.05600 | 0.489 | 159 | -0.114 | 1.0000 |
| Mental Health | Physical | -0.38990 | 0.414 | 160 | -0.942 | 1.0000 |
|  | Romantic | -0.38058 | 0.523 | 162 | -0.728 | 1.0000 |
|  | Social Situations | -1.08791 | 0.408 | 159 | -2.664 | 0.2214 |
| Physical Health | Romantic | 0.00932 | 0.521 | 160 | 0.018 | 1.0000 |
|  | Social Situations | -0.69801 | 0.413 | 157 | -1.689 | 1.0000 |
| Romantic Relationships | Social Situations | -0.70733 | 0.527 | 161 | -1.341 | 1.0000 |

### **Table A.3.5c:** *Results for Hypothesis 1b: Scenario domain and emotion regulation efficacy, Model comparisons*

|  | *npar* | AIC | BIC | Log Likelihood | Deviance | Chi-Square | Df | *P* |
| --- | --- | --- | --- | --- | --- | --- | --- | --- |
| Baseline | 3 | 623.21 | 632.62 | -308.61 | 617.21 |  |  |  |
| Random effects | 10 | 620.92 | 652.28 | -300.46 | 600.92 | 16.291 | 7 | 0.02259 |
| Random effects controlling for affect | 11 | 580.63 | 615.12 | -279.31 | 558.63 | 42.29 | 1 | <.0001 |

### **Table A.3.6a:** *Results for Hypothesis 1c: Scenario domain and reappraisal efficacy, Model results*

| Random Effects |  |  |  |  |
| --- | --- | --- | --- | --- |
|  | Groups | Name | Variance | SD |
| *Null model* |  |  |  |  |
|  | ParticipantID | Intercept | 1.759 | 1.326 |
|  | Residual |  | 2.100 | 1.449 |
| *Random intercepts model* |  |  |  |  |
|  | ParticipantID | Intercept | 1.568 | 1.252 |
|  | Residual |  | 1.954 | 1.398 |
| *Random intercepts model controlling for affect* |  |  |  |  |
|  | ParticipantID | Intercept | 1.869 | 1.367 |
|  | Residual |  | 1.763 | 1.328 |

| Fixed Effects |  |  |  |  |
| --- | --- | --- | --- | --- |
|  |  | *B* | SE | *t* |
| *Null model* |  |  |  |  |
|  | Intercept | 3.8040 | 0.5106 | 7.45 |
| *Random intercepts model* |  |  |  |  |
|  | Intercept | 4.1788 | 0.5488 | 7.614 |
|  | Discrimination | -0.5235 | 0.5720 | -0.915 |
|  | Family & Home Life | -0.5216 | 0.3738 | -1.395 |
|  | Finances | 1.0669 | 0.4761 | 2.241 |
|  | Mental Health | -0.7715 | 0.3860 | -1.999 |
|  | Physical Health | -0.7459 | 0.3986 | -1.871 |
|  | Romantic Relationships | -0.3790 | 0.5198 | -0.729 |
|  | Social Situations | -0.4955 | 0.3985 | -1.243 |
| *Random intercepts model controlling for affect* |  |  |  |  |
|  | Intercept | 1.5760 | 0.8526 | 1.848 |
|  | PreEMA | 0.4498 | 0.1090 | 4.125 |
|  | Discrimination | -0.4215 | 0.5438 | -0.775 |
|  | Family & Home Life | -0.1901 | 0.3639 | -0.522 |
|  | Finances | 1.2735 | 0.4551 | 2.799 |
|  | Mental Health | -0.3057 | 0.3831 | -0.798 |
|  | Physical Health | -0.4742 | 0.3842 | -1.234 |
|  | Romantic Relationships | -0.2273 | 0.4963 | -0.458 |
|  | Social Situations | -0.2534 | 0.3829 | -0.662 |

**Table A.3.6b**

*Results for Hypothesis 1c: Scenario domain and reappraisal efficacy, Pairwise comparisons*

| Domain |  | *B* | SE | df | *t* | *P* |
| --- | --- | --- | --- | --- | --- | --- |
| Academics/Work/Career | Discrimination | 0.52350 | 0.572 | 156 | 0.914 | 1.0000 |
|  | Family/Home | 0.52161 | 0.374 | 156 | 1.394 | 1.0000 |
|  | Finances | -1.06691 | 0.477 | 156 | -2.238 | 0.5857 |
|  | Mental | 0.77151 | 0.386 | 157 | 1.996 | 1.0000 |
|  | Physical | 0.74594 | 0.399 | 156 | 1.870 | 1.0000 |
|  | Romantic | 0.37898 | 0.522 | 161 | 0.726 | 1.0000 |
|  | Social Situations | 0.49549 | 0.399 | 156 | 1.243 | 1.0000 |
| Discrimination | Family/Home | -0.00189 | 0.576 | 156 | -0.003 | 1.0000 |
|  | Finances | -1.59041 | 0.623 | 156 | -2.554 | 0.2788 |
|  | Mental | 0.24801 | 0.580 | 157 | 0.428 | 1.0000 |
|  | Physical | 0.22244 | 0.575 | 156 | 0.387 | 1.0000 |
|  | Romantic | -0.14452 | 0.663 | 159 | -0.218 | 1.0000 |
|  | Social Situations | -0.02802 | 0.583 | 156 | -0.048 | 1.0000 |
| Family & Home Life | Finances | -1.58852 | 0.490 | 157 | -3.241 | 0.0378 |
|  | Mental | 0.24990 | 0.362 | 157 | 0.690 | 1.0000 |
|  | Physical | 0.22433 | 0.407 | 157 | 0.551 | 1.0000 |
|  | Romantic | -0.14263 | 0.525 | 161 | -0.272 | 1.0000 |
|  | Social Situations | -0.02613 | 0.400 | 157 | -0.065 | 1.0000 |
| Finances | Mental | 1.83842 | 0.493 | 158 | 3.726 | 0.0073 |
|  | Physical | 1.81285 | 0.475 | 156 | 3.813 | 0.0055 |
|  | Romantic | 1.44589 | 0.578 | 160 | 2.499 | 0.3094 |
|  | Social Situations | 1.56239 | 0.487 | 157 | 3.208 | 0.0405 |
| Mental Health | Physical | -0.02557 | 0.411 | 157 | -0.062 | 1.0000 |
|  | Romantic | -0.39253 | 0.526 | 161 | -0.746 | 1.0000 |
|  | Social Situations | -0.27603 | 0.406 | 156 | -0.680 | 1.0000 |
| Physical Health | Romantic | -0.36696 | 0.524 | 160 | -0.701 | 1.0000 |
|  | Social Situations | -0.25045 | 0.411 | 156 | -0.609 | 1.0000 |
| Romantic Relationships | Social Situations | 0.11650 | 0.531 | 160 | 0.220 | 1.0000 |

### **Table A.3.6c:** *Results for Hypothesis 1c: Scenario domain and reappraisal efficacy, Model comparisons*

|  | *npar* | AIC | BIC | Log Likelihood | Deviance | Chi-Square | Df | *P* |
| --- | --- | --- | --- | --- | --- | --- | --- | --- |
| Baseline | 3 | 632.41 | 641.82 | -313.21 | 626.41 |  |  |  |
| Random effects | 10 | 626.78 | 658.13 | -303.39 | 606.78 | 19.637 | 7 | .0064 |
| Random effects controlling for affect | 11 | 612.06 | 646.55 | -295.03 | 590.06 | 16.719 | 1 | <.0001 |

### **Table A.3.7a:** *Results for Hypotheses 2a and 2b: Writing demand and affect score, Model results*

| Random Effects |  |  |  |  |
| --- | --- | --- | --- | --- |
|  | Groups | Name | Variance | SD |
| *Null model* |  |  |  |  |
|  | ParticipantID | Intercept | 0.3600 | 0.6000 |
|  | Residual |  | 0.6712 | 0.8193 |
| *Random intercepts model* |  |  |  |  |
|  | ParticipantID | Intercept | 0.2682 | 0.5179 |
|  | Residual |  | 0.5425 | 0.7366 |

| Fixed Effects |  |  |  |  |
| --- | --- | --- | --- | --- |
|  |  | *B* | SE | *t* |
| *Null model* |  |  |  |  |
|  | Intercept | 1.99561 | 0.44827 | 4.452 |
|  | PreEMA | 0.58859 | 0.07238 | 8.133 |
| *Random intercepts model* |  |  |  |  |
|  | Intercept | 1.62908 | 0.46979 | 3.468 |
|  | PreEMA | 0.66251 | 0.06692 | 9.901 |
|  | Long | 0.29831 | 0.28891 | 1.033 |
|  | One-letter | 0.02794 | 0.20546 | 0.136 |
|  | Two-letter | -0.15074 | 0.21769 | -0.692 |
|  | Write your own | -1.59735 | 0.30099 | -5.307 |

### **Table A.3.7b:** *Results for Hypotheses 2a and 2b: Writing demand and affect score, Pairwise comparisons with contrasts*

| Contrast | *B* | SE | df | *t* | *P* |
| --- | --- | --- | --- | --- | --- |
| No writing - Fill-in-the-blank | -0.0614 | 0.193 | 140 | -0.318 | 0.7508 |
| No writing - More writing | 0.5881 | 0.195 | 141 | 3.024 | 0.0030 |
| Fill-in-the-blank - More writing | 0.6495 | 0.240 | 138 | 2.703 | 0.0077 |

### **Table A.3.7c:** *Results for Hypotheses 2a and 2b: Writing demand and affect score, Model comparison*

|  | *npar* | AIC | BIC | Log Likelihood | Deviance | Chi-Square | Df | *P* |
| --- | --- | --- | --- | --- | --- | --- | --- | --- |
| Baseline | 4 | 389.79 | 401.86 | -190.89 | 381.79 |  |  |  |
| Random effects | 8 | 361.04 | 385.18 | -172.52 | 345.04.8 | 36.746 | 4 | <.001 |

### **Table A.3.8a:** *Results for Hypotheses 2a and 2b: Writing demand and emotion regulation efficacy, Model results*

| Random Effects |  |  |  |  |
| --- | --- | --- | --- | --- |
|  | Groups | Name | Variance | SD |
| *Null model* |  |  |  |  |
|  | ParticipantID | Intercept | 0.462 | 0.6797 |
|  | Residual |  | 2.045 | 1.4300 |
| *Random intercepts model* |  |  |  |  |
|  | ParticipantID | Intercept | 0.4957 | 0.7041 |
|  | Residual |  | 2.0317 | 1.4254 |

| Fixed Effects |  |  |  |  |
| --- | --- | --- | --- | --- |
|  |  | *B* | SE | *t* |
| *Null model* |  |  |  |  |
|  | Intercept | 5.4300 | 0.2978 | 18.24 |
| *Random intercepts model* |  |  |  |  |
|  | Intercept | 5.5701 | 0.4637 | 12.014 |
|  | Long | 0.4635 | 0.5572 | 0.832 |
|  | One-letter | -0.2072 | 0.3887 | -0.533 |
|  | Two-letter | -0.1117 | 0.4209 | -0.265 |
|  | Write your own | 0.7562 | 0.5808 | 1.302 |

### **Table A.3.8b:** *Results for Hypotheses 2a and 2b: Writing demand and emotion regulation efficacy, Pairwise comparisons with grouping by contrasts*

| Contrast | *B* | SE | df | *t* | *P* |
| --- | --- | --- | --- | --- | --- |
| No writing - Fill-in-the-blank | -0.159 | 0.371 | 143 | -0.430 | 0.6677 |
| No writing - More writing | -0.769 | 0.374 | 143 | -2.058 | 0.0414 |
| Fill-in-the-blank - More writing | -0.610 | 0.465 | 140 | -1.311 | 0.1919 |

### **Table A.3.8c:** *Results for Hypotheses 2a and 2b: Writing demand and emotion regulation efficacy, Model comparison*

|  | *npar* | AIC | BIC | Log Likelihood | Deviance | Chi-Square | Df | *P* |
| --- | --- | --- | --- | --- | --- | --- | --- | --- |
| Baseline | 3 | 552.03 | 561.09 | -273.02 | 546.03 |  |  |  |
| Random effects | 7 | 555.39 | 576.51 | -270.69 | 541.39 | 4.6462 | 4 | 0.3256 |

### **Table A.3.9a:** *Results for Hypotheses 2a and 2b: Writing demand and reappraisal efficacy, Model results*

| Random Effects |  |  |  |  |
| --- | --- | --- | --- | --- |
|  | Groups | Name | Variance | SD |
| *Null model* |  |  |  |  |
|  | ParticipantID | Intercept | 1.837 | 1.355 |
|  | Residual |  | 2.000 | 1.414 |
| *Random intercepts model* |  |  |  |  |
|  | ParticipantID | Intercept | 1.788 | 1.337 |
|  | Residual |  | 2.024 | 1.423 |

| Fixed Effects |  |  |  |  |
| --- | --- | --- | --- | --- |
|  |  | *B* | SE | *t* |
| *Null model* |  |  |  |  |
|  | Intercept | 3.8033 | 0.5197 | 7.319 |
| *Random intercepts model* |  |  |  |  |
|  | Intercept | 3.4799 | 0.6262 | 5.557 |
|  | Long | 0.6801 | 0.5562 | 1.223 |
|  | One-letter | 0.3458 | 0.3926 | 0.881 |
|  | Two-letter | 0.3273 | 0.4201 | 0.779 |
|  | Write your own | -0.1472 | 0.5799 | -0.254 |

### **Table A.3.9b:** *Results for Hypotheses 2a and 2b: Writing demand and reappraisal efficacy, Pairwise comparisons with grouping by contrasts*

| Contrast | *B* | SE | df | *t* | *P* |
| --- | --- | --- | --- | --- | --- |
| No writing - Fill-in-the-blank | 0.3366 | 0.370 | 140 | 0.909 | 0.3648 |
| No writing - More writing | 0.0701 | 0.373 | 140 | 0.188 | 0.8513 |
| Fill-in-the-blank - More writing | -0.2665 | 0.464 | 139 | -0.574 | 0.5668 |

### **Table A.3.9c:** *Results for Hypotheses 2a and 2b: Writing demand and reappraisal efficacy, Model comparison*

|  | *npar* | AIC | BIC | Log Likelihood | Deviance | Chi-Square | Df | *P* |
| --- | --- | --- | --- | --- | --- | --- | --- | --- |
| Baseline | 3 | 557.17 | 566.23 | -275.59 | 551.17 |  |  |  |
| Random effects | 7 | 562.66 | 583.78 | -274.33 | 548.66 | 2.5166 | 4 | 0.6417 |

### **Table A.3.10a:** *Results for Hypothesis 3a: Post-CBM-I recommendation and affect score, Model results*

| Random Effects |  |  |  |  |
| --- | --- | --- | --- | --- |
|  | Groups | Name | Variance | SD |
| *Null model* |  |  |  |  |
|  | ParticipantID | Intercept | 0.2595 | 0.5094 |
|  | Residual |  | 0.5775 | 0.7600 |
| *Random intercepts model* |  |  |  |  |
|  | ParticipantID | Intercept | 0.2696 | 0.5193 |
|  | Residual |  | 0.5792 | 0.7610 |

| Fixed Effects |  |  |  |  |
| --- | --- | --- | --- | --- |
|  |  | *B* | SE | *t* |
| *Null model* |  |  |  |  |
|  | Intercept | 1.44764 | 0.39216 | 3.691 |
|  | PreEMA | 0.69566 | 0.06386 | 10.894 |
| *Random intercepts model* |  |  |  |  |
|  | Intercept | 1.37738 | 0.40933 | 3.365 |
|  | PreEMA | 0.69325 | 0.06585 | 10.528 |
|  | Resource | 0.14534 | 0.14870 | 0.977 |
|  | Tip | 0.14948 | 0.15100 | 0.990 |

### **Table A.3.10b:** *Results for Hypothesis 3a: Post-CBM-I recommendation and affect score, Pairwise comparisons*

| Recommendation | *B* | SE | df | *t* | *P* |
| --- | --- | --- | --- | --- | --- |
| ER - Resource | -0.14534 | 0.149 | 149 | -0.975 | 0.9755 |
| ER - Tip | -0.14948 | 0.151 | 149 | -0.987 | 0.9755 |
| Resource - Tip | -0.00414 | 0.164 | 151 | -0.025 | 0.9799 |

### **Table A.3.10c:** *Results for Hypothesis 3a: Post-CBM-I recommendation and affect score, Model comparison*

|  | *npar* | AIC | BIC | Log Likelihood | Deviance | Chi-Square | Df | *P* |
| --- | --- | --- | --- | --- | --- | --- | --- | --- |
| Baseline | 4 | 379.82 | 392.05 | -185.91 | 371.82 |  |  |  |
| Random effects | 6 | 382.46 | 400.80 | -185.23 | 370.46 | 1.3588 | 2 | 0.5069 |

### **Table A.3.11a:** *Results for Hypothesis 3a: Post-CBM-I recommendation and emotion regulation efficacy, Model results*

| Random Effects |  |  |  |  |
| --- | --- | --- | --- | --- |
|  | Groups | Name | Variance | SD |
| *Null model* |  |  |  |  |
|  | ParticipantID | Intercept | 0.505 | 0.7106 |
|  | Residual |  | 2.063 | 1.4363 |
| *Random intercepts model* |  |  |  |  |
|  | ParticipantID | Intercept | 1.0446 | 1.0220 |
|  | Residual |  | 0.8626 | 0.9288 |

| Fixed Effects |  |  |  |  |
| --- | --- | --- | --- | --- |
|  |  | *B* | SE | *t* |
| *Null model* |  |  |  |  |
|  | Intercept | 5.4324 | 0.2999 | 18.11 |
| *Random intercepts model* |  |  |  |  |
|  | Intercept | 5.48424 | 0.33597 | 16.324 |
|  | Resource | -0.21469 | 0.27838 | -0.771 |
|  | Tip | 0.08053 | 0.28177 | 0.286 |

### **Table A.3.11b:** *Results for Hypothesis 3a: Post-CBM-I recommendation and emotion regulation efficacy, Pairwise comparisons*

| Recommendation | *B* | SE | df | *t* | *P* |
| --- | --- | --- | --- | --- | --- |
| ER - Resource | 0.2147 | 0.279 | 150 | 0.770 | 0.9751 |
| ER - Tip | -0.0805 | 0.282 | 149 | -0.285 | 0.9751 |
| Resource - Tip | -0.2952 | 0.299 | 150 | -0.987 | 0.9751 |

### **Table A.3.11c:** *Results for Hypothesis 3a: Post-CBM-I recommendation and emotion regulation efficacy, Model comparison*

|  | *npar* | AIC | BIC | Log Likelihood | Deviance | Chi-Square | Df | *P* |
| --- | --- | --- | --- | --- | --- | --- | --- | --- |
| Baseline | 3 | 574.25 | 583.42 | -284.12 | 568.25 |  |  |  |
| Random effects | 5 | 577.23 | 592.51 | -283.61 | 567.23 | 1.0231 | 2 | 0.5996 |

### **Table A.3.12a:** *Results for Hypothesis 3b: Post-CBM-I recommendation and reappraisal efficacy, Model results*

| Random Effects |  |  |  |  |
| --- | --- | --- | --- | --- |
|  | Groups | Name | Variance | SD |
| *Null model* |  |  |  |  |
|  | ParticipantID | Intercept | 1.813 | 1.346 |
|  | Residual |  | 2.054 | 1.433 |
| *Random intercepts model* |  |  |  |  |
|  | ParticipantID | Intercept | 1.866 | 1.366 |
|  | Residual |  | 2.033 | 1.426 |

| Fixed Effects |  |  |  |  |
| --- | --- | --- | --- | --- |
|  |  | *B* | SE | *t* |
| *Null model* |  |  |  |  |
|  | Intercept | 3.8102 | 0.5174 | 7.364 |
| *Random intercepts model* |  |  |  |  |
|  | Intercept | 3.8037 | 0.5411 | 7.029 |
|  | Resource | -0.2094 | 0.2774 | -0.755 |
|  | Tip | 0.3310 | 0.2803 | 1.181 |

### **Table A.3.12b:** *Results for Hypothesis 3b: Post-CBM-I recommendation and reappraisal efficacy, Pairwise comparisons*

| Recommendation | *B* | SE | df | *t* | *P* |
| --- | --- | --- | --- | --- | --- |
| ER - Resource | 0.209 | 0.278 | 149 | 0.754 | 0.4798 |
| ER - Tip | -0.331 | 0.281 | 148 | -1.180 | 0.4798 |
| Resource - Tip | -0.540 | 0.298 | 149 | -1.815 | 0.2146 |

### **Table A.3.12c:** *Results for Hypothesis 3b: Post-CBM-I recommendation and reappraisal efficacy, Model comparison*

|  | *npar* | AIC | BIC | Log Likelihood | Deviance | Chi-Square | Df | *P* |
| --- | --- | --- | --- | --- | --- | --- | --- | --- |
| Baseline | 3 | 582.41 | 591.58 | -288.20 | 576.41 |  |  |  |
| Random effects | 5 | 583.07 | 598.35 | -286.54 | 573.07 | 3.3386 | 2 | 0.1884 |
