# Supplementary material for: Examining State Affective and Cognitive Outcomes Following Brief Mobile Phone-Based Training Sessions to Reduce Anxious Interpretations
Source: Cognit Ther Res. 2025 Jun 16;50(1):96–118. doi: 10.1007/s10608-025-10623-z (PMC12890198; doi:10.1007/s10608-025-10623-z)
Supplement: Supplementary file 2 [file 10608_2025_10623_MOESM2_ESM.docx]

**Supplemental Material B: Additional results related to primary analyses**

Table of Contents

[Results including all pre-microdose affect scores (1-7) 4](#_Toc176377674)

[Results 4](#_Toc176377675)

[Tables 6](#_Toc176377676)

[Table B.1.1: Demographic Characteristics 6](#_Toc176377677)

[Table B.1.2: Descriptive Statistics 7](#_Toc176377678)

[Table B.1.3: Percent of total variance explained by fixed and random effects and intraclass correlation (ICC) for all models 8](#_Toc176377679)

[Table B.1.4a: Results for Hypothesis 1a: Scenario domain and affect score, Pairwise comparisons 9](#_Toc176377680)

[Table B.1.4b: Results for Hypothesis 1a: Scenario domain and affect score, Model comparison 10](#_Toc176377681)

[Table B.1.5a: Results for Hypothesis 1b: Scenario domain and emotion regulation efficacy, Pairwise 11](#_Toc176377682)

[comparisons 11](#_Toc176377683)

[Table B.1.5b: Results for Hypothesis 1b: Scenario domain and emotion regulation efficacy, Model comparisons 12](#_Toc176377684)

[Table B.1.6a: Results for Hypothesis 1c: Scenario domain and reappraisal, Pairwise comparisons 13](#_Toc176377685)

[Table B.1.6b: Results for Hypothesis 1c: Scenario domain and reappraisal efficacy, Model comparisons 14](#_Toc176377686)

[Table B.1.7a: Results for Hypotheses 2a and 2b: Writing demand and affect score, Pairwise comparisons with contrasts 14](#_Toc176377687)

[Table B.1.7b: Results for Hypotheses 2a and 2b: Writing demand and affect score, Model comparison 15](#_Toc176377688)

[Table B.1.8a: Results for Hypotheses 2a and 2b: Writing demand and emotion regulation efficacy, Pairwise comparisons with grouping by contrasts 15](#_Toc176377689)

[Table B.1.8b: Results for Hypotheses 2a and 2b: Writing demand and emotion regulation efficacy, Model comparison 15](#_Toc176377690)

[Table B.1.9a: Results for Hypotheses 2a and 2b: Writing demand and reappraisal efficacy, Pairwise comparisons with grouping by contrasts 16](#_Toc176377691)

[Table B.1.9b: Results for Hypotheses 2a and 2b: Writing demand and reappraisal efficacy, Model comparison 16](#_Toc176377692)

[Model Variances & Intraclass Correlation Coefficients 16](#_Toc176377693)

[Table B.2: Percent of total variance explained by fixed and random effects and intraclass correlation (ICC) for all models 16](#_Toc176377694)

[Null & Random Intercepts Model Results 17](#_Toc176377695)

[Table B.3.1: Results for Hypothesis 1a: Scenario domain and affect score, Model results 17](#_Toc176377696)

[Table B.3.2: Results for Hypothesis 1b: Scenario domain and emotion regulation efficacy, Model results 19](#_Toc176377697)

[Table B.3.3: Results for Hypothesis 1c: Scenario domain and reappraisal, Model results 21](#_Toc176377698)

[Table B.3.4: Results for Hypotheses 2a and 2b: Writing demand and affect score, Model results 23](#_Toc176377699)

[Table B.3.5: Results for Hypotheses 2a and 2b: Writing demand and emotion regulation efficacy, Model results 24](#_Toc176377700)

[Table B.3.5: Results for Hypotheses 2a and 2b: Writing demand and reappraisal efficacy, Model results 25](#_Toc176377701)

# Results including all pre-microdose affect scores (1-7)

## **Results**

**Demographics**

Ninety-seven participants (out of 104 included in our sample) provided demographic information. Most participants identified as White/European (69.07%), non-Hispanic (95.88%), women (84.54%). Participant ages ranged from 19 to 62 (*M* = 31.51, *SD* = 11.49). See Table B.1.1 for full demographic information.

**Microdose Characteristics**

Across the full sample, participants completed 2,759 microdoses in total. Microdoses missing either pre- or post-microdose affect, reappraisal efficacy, or emotion regulation efficacy scores (as a result of either participants choosing to skip the question in the app or technical issues in the data being recorded) were removed (*n* = 99), leaving 2,660 microdoses completed across 105 participants. The total number of microdoses completed by participants during their study participation ranged from two (4.8% of total expected) to 79 (over 100% of total expected) microdoses (*M* = 25.30, *SD* = 18.77). Due to a technical error with how the mobile application collected data at the beginning of the study (October 2022 - January 2023), writing demand and recommendation data are missing for a portion of the microdoses. Specifically, the microdose stressor domain is known for all but one microdose (99.96%), but writing demand is known for 1,803 microdoses (67.78%) and recommendation type is known for 1,910 microdoses (71.80%). On average, participants rated their affect as being somewhat positive both immediately prior to (5.05 out of 7) and immediately after (5.06 out of 7) completing a microdose; participants rated their reappraisal efficacy (4.54 out of 7) and their emotion regulation efficacy (4.86 out of 7) as being somewhat better immediately after completing a microdose. Academics/work/career development was the most frequently chosen domain (*n =* 508); the least chosen domains were discrimination (*n =* 252) and finances (*n =* 284). See Table B.1.2 for full descriptives.

For each model, the total variance explained by the fixed effects and the total variance explained by both fixed and random effects, as well as the intraclass correlation coefficient (ICC), is reported in Table B.1.3.

**How did post-microdose affect scores (controlling for pre-microdose affect) differ based on domain? (H1a)**

Post-microdose affect scores were significantly more positive for the academics/work/career development, family and home life, finances, mental health, physical health, and social situations stressor domains compared to the romantic relationships and discrimination domains; see Table B.1.4a for pairwise comparisons. No other pairwise comparisons across domains were significant. The random intercepts model with domain as a predictor performed significantly better than the null model with no predictor (Chi-square=72.86, df=7, *P<*.001). See Table B.1.4b for full model comparison, including Akaike Information Criterion (AIC) values.

**How did post-microdose emotion regulation efficacy differ based on domain? (H1b)**

Emotion regulation efficacy scores were significantly higher following microdoses completed in the academics/work/career development, family and home life, finances, mental health, physical health, and social situations stressor domains compared to the romantic relationships and discrimination domains; see Table B.1.5a for pairwise comparisons. No other pairwise comparisons across domains were significant. The random intercepts model with domain as a predictor performed significantly better than the null model with no predictor (Chi-square=185.40, df=35, *P<*.001). Additionally, we ran a random intercepts model with pre-microdose affect score as a predictor along with domain to control for the effect of affect on emotion regulation efficacy. Results were virtually the same with the same significant pairwise comparisons, suggesting that we do not need to control for pre-session affect (i.e., pre-session affect is not affecting the relationship between domain and emotion regulation efficacy). See Table B.1.5b for full model comparisons.

**How did post-microdose reappraisal efficacy differ based on domain? (H1c)**

Reappraisal scores were significantly higher after microdoses completed in the academics/work/career development, family and home life, finances, mental health, physical health, and social situations stressor domains compared to the romantic relationships and discrimination domains; see Table B.1.6a pairwise comparisons. No other pairwise comparisons across domains were significant. The random intercepts model with domain as a predictor performed significantly better than the null model with no predictor (Chi-square=124.24, df=7, *P<*.001). Additionally, we ran a random intercepts model with pre-microdose affect score as a predictor along with domain to control for the effect of affect on reappraisal efficacy. Results were virtually the same with the same significant pairwise comparisons, suggesting that we do not need to control for pre-session affect (i.e., pre-session affect is not affecting the relationship between domain and reappraisal efficacy). See Table B.1.6b for full model comparisons.

**How did post-microdose affect scores (controlling for pre-microdose affect) differ based on writing demand? (H2a and H2b)**

Writing demand data was collected for 85 (out of 105) participants, including 1,803 (out of 2,660) microdoses. Analyses were conducted only on the subset of microdoses that contain writing demand data.

Both scenarios with no writing (one- and two-letters missing; *B=*0.25, *SE=*.08, *P=*.001) and some writing (fill-in-the-blank scenarios; *B=*0.27, *SE=*.10, *P=*.007) were associated with significantly more positive post-microdose affect than scenarios containing a large amount of writing (write your own and long scenarios). There was no significant difference between post-microdose affect scores for scenarios with no writing (one- and two-letters missing) and fill-in-the-blank scenarios (*B=*-0.02, *SE=*.08, *P=*.77). See Table B.1.7a for pairwise comparisons. The random intercepts model with writing demand as a predictor performed significantly better than the null model with no predictor (Chi-square=17.56, df=4, *P*=.002). See Table B.1.7b for full model comparison.

**How did post-microdose emotion regulation efficacy differ based on writing demand? (H2a and H2b)**

There was no significant difference in emotion regulation efficacy scores across writing demands. See Table B.1.8a for pairwise comparisons. However, the random intercepts model with writing demands as a predictor performed significantly better than the null model with no predictor (Chi-square=22.72, df=4, *P*<.001). See Table B.1.8b for full model comparison. Considering that no pairwise comparisons were significant, we concluded that neither model is a good fit for the data even though the random intercepts model may outperform the null model.

**How did post-microdose reappraisal efficacy differ based on writing demand? (H2a and H2b)**

There was no significant difference in reappraisal efficacy scores across writing demands. See Table B.1.9a for pairwise comparisons. The random intercepts model with writing demands as a predictor did not perform significantly better than the null model with no predictor (Chi-square=6.78, df=4, *P*=.15). See Table B.1.9b for full model comparison.

## **Tables**

| **Table B.1.1:** *Demographic Characteristics* |  |
| --- | --- |
| Characteristic | *n* (%) |
| Gender:  Man  Woman  Transgender Man  Transgender Woman  Other identity  Race:  White/European Origin  East Asian  South Asian  Other or Unknown  Black/African Origin  Participant selected more than one race  American Indian/Alaska Native  Native Hawaiian/Pacific Islander  Ethnicity:  Not Hispanic or Latino  Hispanic or Latino | 14 (14.43%)  82 (84.54%)  0 (0%)  0 (0%)  1 (1.03%)    67 (69.07%)  9 (9.28%)  7 (7.23%)  7 (7.23%)  4 (4.12%)  3 (3.09%)  0 (0%)  0 (0%)    93 (95.88%)  4 (3.96%) |

### **Table B.1.2:** *Descriptive Statistics*

|  | *n* | Mean | SD | Range |
| --- | --- | --- | --- | --- |
| Microdoses per Domain*  Academics/Work/Career Development  Discrimination  Family & Home Life  Finances  Mental Health  Physical Health  Romantic Relationships  Social Situations | 508  252  371  284  294  343  295  312 | 332.4 | 79.8 | 256 |
| Microdoses per Scenario Type*  Fill-in-the-blank  Long scenario  One-letter  Two-letter  Write-your-own  Not available | 117  102  1117  422  45  857 | 360.6 | 447.7 | 1072 |
| Microdoses per Recommendation Type*  Emotion Regulation  Resource  Tip  Not available | 524  691  695  750 | 636.7 | 97.6 | 226 |
| Ecological Momentary Assessment Scores  Pre-Microdose EMA [out of 7]  Post-Microdose EMA [out of 7]  Cognitive Reappraisal Efficacy [out of 7]  Emotion Regulation Efficacy [out of 7]    *Mean, SD, and range based off of microdoses that have the data available |  | 5.05  5.06  4.54  4.86 | 1.28  1.22  1.59  1.36 | 6  6  6  7 |

### **Table B.1.3***: Percent of total variance explained by fixed and random effects and intraclass correlation (ICC) for all models*

|  |  | Fixed effects | Fixed and random effects | ICC |
| --- | --- | --- | --- | --- |
| Test | Model |  |  |  |
| Scenario domain and affect score | Null | 49.32% | 57.04% | 0.152 |
|  | Random Intercepts | 50.52% | 58.18% | 0.155 |
| Scenario domain and ER efficacy | Null | 0.00% | 55.11% | 0.551 |
|  | Random Intercepts | 1.04% | 55.95% | 0.555 |
|  | Random Intercepts controlling for affect | 7.53% | 57.05% | 0.535 |
| Scenario domain and reappraisal efficacy | Null | 0.00% | 52.49% | 0.525 |
|  | Random Intercepts | 2.38% | 54.48% | 0.535 |
|  | Random Intercepts controlling for affect | 6.61% | 55.04% | 0.519 |
| Scenario format and affect score | Null | 47.73% | 56.11% | 0.16 |
|  | Random Intercepts | 48.13% | 56.53% | 0.162 |
| Scenario format and ER efficacy | Null | 0.00% | 53.99% | 0.54 |
|  | Random Intercepts | 0.71% | 53.90% | 0.536 |
| Scenario format and reappraisal efficacy | Null | 0.00% | 53.75% | 0.538 |
|  | Random Intercepts | 0.19% | 53.79% | 0.537 |

### **Table B.1.4a:** *Results for Hypothesis 1a: Scenario domain and affect score, Pairwise comparisons*

| Domain |  | *B* | SE | df | *t* | *P* |
| --- | --- | --- | --- | --- | --- | --- |
| Academics/Work/Career | Discrimination | 0.2089 | 0.0610 | 2636 | 3.427 | 0.0124 |
|  | Family/Home | 0.0330 | 0.0523 | 2603 | 0.631 | 1.0000 |
|  | Finances | 0.0176 | 0.0566 | 2596 | 0.311 | 1.0000 |
|  | Mental | 0.0446 | 0.0559 | 2595 | 0.798 | 1.0000 |
|  | Physical | -0.0823 | 0.0534 | 2601 | -1.542 | 1.0000 |
|  | Romantic | 0.3552 | 0.0564 | 2614 | 6.299 | <.0001 |
|  | Social Situations | -0.0261 | 0.0551 | 2598 | -0.473 | 1.0000 |
| Discrimination | Family/Home | -0.1759 | 0.0640 | 2626 | -2.747 | 0.1090 |
|  | Finances | -0.1913 | 0.0676 | 2622 | -2.831 | 0.0887 |
|  | Mental | -0.1643 | 0.0673 | 2626 | -2.439 | 0.2512 |
|  | Physical | -0.2912 | 0.0647 | 2620 | -4.503 | 0.0002 |
|  | Romantic | 0.1463 | 0.0677 | 2634 | 2.160 | 0.4937 |
|  | Social Situations | -0.2350 | 0.0660 | 2621 | -3.561 | 0.0079 |
| Family & Home Life | Finances | -0.0154 | 0.0598 | 2585 | -0.258 | 1.0000 |
|  | Mental | 0.0116 | 0.0594 | 2587 | 0.196 | 1.0000 |
|  | Physical | -0.1153 | 0.0566 | 2579 | -2.036 | 0.5853 |
|  | Romantic | 0.3222 | 0.0598 | 2608 | 5.387 | <.0001 |
|  | Social Situations | -0.0591 | 0.0584 | 2584 | -1.012 | 1.0000 |
| Finances | Mental | 0.0270 | 0.0633 | 2590 | 0.427 | 1.0000 |
|  | Physical | -0.0999 | 0.0607 | 2582 | -1.645 | 1.0000 |
|  | Romantic | 0.3376 | 0.0632 | 2590 | 5.341 | <.0001 |
|  | Social Situations | -0.0437 | 0.0623 | 2586 | -0.700 | 1.0000 |
| Mental Health | Physical | -0.1269 | 0.0604 | 2585 | -2.101 | 0.5362 |
|  | Romantic | 0.3105 | 0.0631 | 2604 | 4.920 | <.0001 |
|  | Social Situations | -0.0707 | 0.0621 | 2591 | -1.138 | 1.0000 |
| Physical Health | Romantic | 0.4375 | 0.0605 | 2598 | 7.231 | <.0001 |
|  | Social Situations | 0.0562 | 0.0593 | 2583 | 0.947 | 1.0000 |
| Romantic Relationships | Social Situations | -0.3813 | 0.0621 | 2601 | -6.136 | <.0001 |

### **Table B.1.4b:** *Results for Hypothesis 1a: Scenario domain and affect score, Model comparison*

|  | *npar* | AIC | BIC | Log Likelihood | Deviance | Chi-Square | Df | *P* |
| --- | --- | --- | --- | --- | --- | --- | --- | --- |
| Baseline | 4 | 6238.0 | 6261.5 | -3115.0 | 6230.0 |  |  |  |
| Random effects | 11 | 6179.1 | 6243.9 | -3078.6 | 6157.1 | 72.856 | 7 | <.0001 |

### **Table B.1.5a:** *Results for Hypothesis 1b: Scenario domain and emotion regulation efficacy, Pairwise*

### comparisons

| Domain |  | *B* | SE | df | *t* | *P* |
| --- | --- | --- | --- | --- | --- | --- |
| Academics/Work/Career | Discrimination | 0.3178 | 0.0764 | 2567 | 4.160 | 0.0007 |
|  | Family/Home | 0.0533 | 0.0652 | 2558 | 0.817 | 1.0000 |
|  | Finances | 0.0780 | 0.0704 | 2556 | 1.108 | 1.0000 |
|  | Mental | -0.0167 | 0.0696 | 2555 | -0.240 | 1.0000 |
|  | Physical | -0.0542 | 0.0665 | 2558 | -0.815 | 1.0000 |
|  | Romantic | 0.3529 | 0.0704 | 2561 | 5.014 | <.0001 |
|  | Social Situations | -0.0568 | 0.0686 | 2555 | -0.829 | 1.0000 |
| Discrimination | Family/Home | -0.2645 | 0.0802 | 2563 | -3.299 | 0.0177 |
|  | Finances | -0.2397 | 0.0845 | 2562 | -2.837 | 0.0781 |
|  | Mental | -0.3345 | 0.0842 | 2563 | -3.971 | 0.0015 |
|  | Physical | -0.3720 | 0.0808 | 2560 | -4.602 | 0.0001 |
|  | Romantic | 0.0352 | 0.0848 | 2566 | 0.415 | 1.0000 |
|  | Social Situations | -0.3746 | 0.0825 | 2561 | -4.539 | 0.0001 |
| Family & Home Life | Finances | 0.0247 | 0.0744 | 2554 | 0.332 | 1.0000 |
|  | Mental | -0.0700 | 0.0739 | 2553 | -0.947 | 1.0000 |
|  | Physical | -0.1075 | 0.0705 | 2553 | -1.526 | 1.0000 |
|  | Romantic | 0.2996 | 0.0746 | 2560 | 4.015 | 0.0013 |
|  | Social Situations | -0.1101 | 0.0726 | 2552 | -1.516 | 1.0000 |
| Finances | Mental | -0.0947 | 0.0788 | 2555 | -1.203 | 1.0000 |
|  | Physical | -0.1322 | 0.0755 | 2554 | -1.751 | 1.0000 |
|  | Romantic | 0.2749 | 0.0786 | 2554 | 3.499 | 0.0090 |
|  | Social Situations | -0.1348 | 0.0775 | 2553 | -1.739 | 1.0000 |
| Mental Health | Physical | -0.0375 | 0.0751 | 2553 | -0.499 | 1.0000 |
|  | Romantic | 0.3696 | 0.0787 | 2558 | 4.700 | 0.0001 |
|  | Social Situations | -0.0401 | 0.0773 | 2553 | -0.519 | 1.0000 |
| Physical Health | Romantic | 0.4071 | 0.0753 | 2558 | 5.404 | <.0001 |
|  | Social Situations | -0.0026 | 0.0738 | 2553 | -0.035 | 1.0000 |
| Romantic Relationships | Social Situations | -0.4097 | 0.0774 | 2558 | -5.296 | <.0001 |

### **Table B.1.5b:** *Results for Hypothesis 1b: Scenario domain and emotion regulation efficacy, Model comparisons*

|  | *npar* | AIC | BIC | Log Likelihood | Deviance | Chi-Square | Df | *P* |
| --- | --- | --- | --- | --- | --- | --- | --- | --- |
| Baseline | 3 | 7530.7 | 7548.4 | -3762.4 | 7524.7 |  |  |  |
| Random effects | 10 | 7486.6 | 7545.5 | -3733.3 | 7466.6 | 58.074856 | 7 | <.0001 |
| Random effects controlling for affect | 11 | 7226.3 | 7291.1 | -3602.2 | 7204.3 | 262.31 | 1 | <.0001 |

### **Table B.1.6a:** *Results for Hypothesis 1c: Scenario domain and reappraisal, Pairwise comparisons*

| Domain |  | *B* | SE | df | *t* | *P* |
| --- | --- | --- | --- | --- | --- | --- |
| Academics/Work/Career | Discrimination | 0.78031 | 0.0893 | 2571 | 8.737 | <.0001 |
|  | Family/Home | 0.17460 | 0.0764 | 2562 | 2.286 | 0.3571 |
|  | Finances | 0.16537 | 0.0825 | 2560 | 2.005 | 0.5863 |
|  | Mental | 0.11662 | 0.0816 | 2559 | 1.429 | 1.0000 |
|  | Physical | 0.08809 | 0.0779 | 2562 | 1.131 | 1.0000 |
|  | Romantic | 0.56437 | 0.0825 | 2565 | 6.843 | <.0001 |
|  | Social Situations | -0.00986 | 0.0804 | 2559 | -0.123 | 1.0000 |
| Discrimination | Family/Home | -0.60570 | 0.0938 | 2567 | -6.460 | <.0001 |
|  | Finances | -0.61494 | 0.0989 | 2567 | -6.217 | <.0001 |
|  | Mental | -0.66368 | 0.0986 | 2567 | -6.731 | <.0001 |
|  | Physical | -0.69222 | 0.0946 | 2564 | -7.316 | <.0001 |
|  | Romantic | -0.21593 | 0.0993 | 2571 | -2.176 | 0.4451 |
|  | Social Situations | -0.79016 | 0.0966 | 2566 | -8.182 | <.0001 |
| Family & Home Life | Finances | -0.00923 | 0.0872 | 2558 | -0.106 | 1.0000 |
|  | Mental | -0.05798 | 0.0866 | 2557 | -0.670 | 1.0000 |
|  | Physical | -0.08651 | 0.0825 | 2556 | -1.048 | 1.0000 |
|  | Romantic | 0.38977 | 0.0874 | 2564 | 4.459 | 0.0002 |
|  | Social Situations | -0.18446 | 0.0851 | 2556 | -2.168 | 0.4451 |
| Finances | Mental | -0.04874 | 0.0923 | 2558 | -0.528 | 1.0000 |
|  | Physical | -0.07728 | 0.0886 | 2558 | -0.873 | 1.0000 |
|  | Romantic | 0.39901 | 0.0921 | 2558 | 4.332 | 0.0003 |
|  | Social Situations | -0.17522 | 0.0909 | 2557 | -1.928 | 0.6483 |
| Mental Health | Physical | -0.02853 | 0.0881 | 2557 | -0.324 | 1.0000 |
|  | Romantic | 0.44775 | 0.0922 | 2562 | 4.856 | <.0001 |
|  | Social Situations | -0.12648 | 0.0906 | 2557 | -1.396 | 1.0000 |
| Physical Health | Romantic | 0.47628 | 0.0883 | 2562 | 5.392 | <.0001 |
|  | Social Situations | -0.09795 | 0.0865 | 2557 | -1.132 | 1.0000 |
| Romantic Relationships | Social Situations | -0.57423 | 0.0907 | 2562 | -6.331 | <.0001 |

### **Table B.1.6b:** *Results for Hypothesis 1c: Scenario domain and reappraisal efficacy, Model comparisons*

|  | *npar* | AIC | BIC | Log Likelihood | Deviance | Chi-Square | Df | *P* |
| --- | --- | --- | --- | --- | --- | --- | --- | --- |
| Baseline | 3 | 8442.9 | 8460.5 | -4218.4 | 8436.9 |  |  |  |
| Random effects | 10 | 8332.6 | 8391.5 | -4156.3 | 8312.6 | 124.24 | 7 | <.0001 |
| Random effects controlling for affect | 11 | 8170.4 | 8235.1 | -4074.2 | 8148.4 | 164.25 | 1 | <.0001 |

### **Table B.1.7a:** *Results for Hypotheses 2a and 2b: Writing demand and affect score, Pairwise comparisons with contrasts*

| Contrast | *B* | SE | df | *t* | *P* |
| --- | --- | --- | --- | --- | --- |
| No writing - Fill-in-the-blank | -0.0227 | 0.0775 | 1764 | -0.293 | 0.7694 |
| No writing - More writing | 0.2486 | 0.0752 | 1762 | 3.308 | 0.0010 |
| Fill-in-the-blank - More writing | 0.2713 | 0.0997 | 1722 | 2.722 | 0.0066 |

### **Table B.1.7b:** *Results for Hypotheses 2a and 2b: Writing demand and affect score, Model comparison*

|  | *npar* | AIC | BIC | Log Likelihood | Deviance | Chi-Square | Df | *P* |
| --- | --- | --- | --- | --- | --- | --- | --- | --- |
| Baseline | 4 | 4325.4 | 4347.4 | -2158.7 | 4317.4 |  |  |  |
| Random effects | 8 | 4315.8 | 4359.8 | -2149.9 | 4299.8 | 17.563 | 4 | 0.0015 |

### **Table B.1.8a:** *Results for Hypotheses 2a and 2b: Writing demand and emotion regulation efficacy, Pairwise comparisons with grouping by contrasts*

| Contrast | *B* | SE | df | *t* | *P* |
| --- | --- | --- | --- | --- | --- |
| No writing - Fill-in-the-blank | -0.11948 | 0.0947 | 1724 | -1.262 | 0.2070 |
| No writing - More writing | -0.11810 | 0.0918 | 1723 | -1.287 | 0.1982 |
| Fill-in-the-blank - More writing | 0.00138 | 0.1211 | 1713 | 0.011 | 0.9909 |

### **Table B.1.8b:** *Results for Hypotheses 2a and 2b: Writing demand and emotion regulation efficacy, Model comparison*

|  | *npar* | AIC | BIC | Log Likelihood | Deviance | Chi-Square | Df | *P* |
| --- | --- | --- | --- | --- | --- | --- | --- | --- |
| Baseline | 3 | 5154.0 | 5170.5 | -2574.0 | 5148.0 |  |  |  |
| Random effects | 7 | 5139.3 | 5177.8 | -2562.7 | 5125.3 | 22.717 | 4 | <0.001 |

### **Table B.1.9a:** *Results for Hypotheses 2a and 2b: Writing demand and reappraisal efficacy, Pairwise comparisons with grouping by contrasts*

| Contrast | *B* | SE | df | *t* | *P* |
| --- | --- | --- | --- | --- | --- |
| No writing - Fill-in-the-blank | 0.0943 | 0.109 | 1726 | 0.865 | 0.3869 |
| No writing - More writing | -0.1282 | 0.106 | 1725 | -1.213 | 0.2254 |
| Fill-in-the-blank - More writing | -0.2225 | 0.139 | 1715 | -1.595 | 0.1108 |

### **Table B.1.9b:** *Results for Hypotheses 2a and 2b: Writing demand and reappraisal efficacy, Model comparison*

|  | *npar* | AIC | BIC | Log Likelihood | Deviance | Chi-Square | Df | *P* |
| --- | --- | --- | --- | --- | --- | --- | --- | --- |
| Baseline | 3 | 5653.0 | 5669.5 | -2823.5 | 5647.0 |  |  |  |
| Random effects | 7 | 5654.2 | 5692.7 | -2820.1 | 5640.2 | 6.7838 | 4 | 0.1478 |

# Model Variances & Intraclass Correlation Coefficients

## **Table B.2**: *Percent of total variance explained by fixed and random effects and intraclass correlation (ICC) for all models*

|  |  | Fixed effects | Fixed and random effects | ICC |
| --- | --- | --- | --- | --- |
| Test | Model |  |  |  |
| Scenario domain and affect score | Null | 36.15% | 49.00% | 0.201 |
|  | Random Intercepts | 38.32% | 50.85% | 0.203 |
| Scenario domain and ER efficacy | Null | 0.00% | 50.06% | 0.501 |
|  | Random Intercepts | 1.71% | 51.25% | 0.504 |
|  | Random Intercepts controlling for affect | 7.09% | 54.47% | 0.510 |
| Scenario domain and reappraisal efficacy | Null | 0.00% | 49.90% | 0.499 |
|  | Random Intercepts | 2.85% | 52.44% | 0.51 |
|  | Random Intercepts controlling for affect | 7.70% | 55.76% | 0.521 |
| Writing demand and affect score | Null | 36.94% | 49.66% | 0.202 |
|  | Random Intercepts | 37.65% | 50.38% | 0.204 |
| Writing demand and ER efficacy | Null | 0.00% | 49.71% | 0.497 |
|  | Random Intercepts | 0.47% | 49.74% | 0.495 |
| Writing demand and reappraisal efficacy | Null | 0.00% | 52.40% | 0.524 |
|  | Random Intercepts | 0.29% | 52.63% | 0.525 |

# Null & Random Intercepts Model Results

## **Table B.3.1:** *Results for Hypothesis 1a: Scenario domain and affect score, Model results*

| Random Effects |  |  |  |  |
| --- | --- | --- | --- | --- |
|  | Groups | Name | Variance | SD |
| *Null model* |  |  |  |  |
|  | ParticipantID | Intercept | 0.1406 | 0.3750 |
|  | Residual |  | 0.5581 | 0.7471 |
| *Random intercepts model* |  |  |  |  |
|  | ParticipantID | Intercept | 0.1376 | 0.3710 |
|  | Residual |  | 0.5399 | 0.7348 |

| Fixed Effects |  |  |  |  |
| --- | --- | --- | --- | --- |
|  |  | *B* | SE | *t* |
| *Null model* |  |  |  |  |
|  | Intercept | 1.7347 | 0.1021 | 16.99 |
|  | PreEMA | 0.6680 | 0.0218 | 30.71 |
| *Random intercepts model* |  |  |  |  |
|  | Intercept | 1.8346 | 0.1055 | 17.398 |
|  | PreEMA | 0.6658 | 0.0215 | 30.964 |
|  | Discrimination | -0.2736 | 0.0791 | -3.459 |
|  | Family & Home Life | -0.0530 | 0.0657 | -0.807 |
|  | Finances | -0.0455 | 0.0723 | -0.630 |
|  | Mental Health | -0.0722 | 0.0689 | -1.048 |
|  | Physical Health | 0.0754 | 0.0668 | 1.127 |
|  | Romantic Relationships | -0.4230 | 0.0696 | -6.076 |
|  | Social Situations | -0.0578 | 0.0696 | -0.831 |

## **Table B.3.2:** *Results for Hypothesis 1b: Scenario domain and emotion regulation efficacy, Model results*

| Random Effects |  |  |  |  |
| --- | --- | --- | --- | --- |
|  | Groups | Name | Variance | SD |
| *Null model* |  |  |  |  |
|  | ParticipantID | Intercept | 0.8163 | 0.9035 |
|  | Residual |  | 0.8144 | 0.9024 |
| *Random intercepts model* |  |  |  |  |
|  | ParticipantID | Intercept | 0.8051 | 0.8973 |
|  | Residual |  | 0.7925 | 0.8902 |
| *Random intercepts model controlling for affect* |  |  |  |  |
|  | ParticipantID | Intercept | 0.7566 | 0.8698 |
|  | Residual |  | 0.7271 | 0.8527 |

| Fixed Effects |  |  |  |  |
| --- | --- | --- | --- | --- |
|  |  | *B* | SE | *t* |
| *Null model* |  |  |  |  |
|  | Intercept | 4.57588 | 0.09606 | 47.63 |
| *Random intercepts model* |  |  |  |  |
|  | Intercept | 4.68669 | 0.10593 | 44.25 |
|  | Discrimination | -0.51637 | 0.09722 | -5.311 |
|  | Family & Home Life | -0.10406 | 0.08009 | -1.299 |
|  | Finances | -0.08060 | 0.08778 | -0.918 |
|  | Mental Health | -0.02083 | 0.08394 | -0.248 |
|  | Physical Health | 0.04216 | 0.08136 | 0.518 |
|  | Romantic Relationships | -0.35132 | 0.08504 | -4.131 |
|  | Social Situations | -0.05548 | 0.08459 | -0.656 |
| *Random intercepts model controlling for affect* |  |  |  |  |
|  | Intercept | 3.43808 | 0.14571 | 23.596 |
|  | PreEMA | 0.30546 | 0.02537 | 12.042 |
|  | Discrimination | -0.58439 | 0.09331 | -6.263 |
|  | Family & Home Life | -0.15754 | 0.07685 | -2.050 |
|  | Finances | -0.15750 | 0.08433 | -1.868 |
|  | Mental Health | -0.04438 | 0.08043 | -0.552 |
|  | Physical Health | -0.01782 | 0.07810 | -0.228 |
|  | Romantic Relationships | -0.36924 | 0.08147 | -4.532 |
|  | Social Situations | -0.10482 | 0.08113 | -1.292 |

## **Table B.3.3:** *Results for Hypothesis 1c: Scenario domain and reappraisal, Model results*

| Random Effects |  |  |  |  |
| --- | --- | --- | --- | --- |
|  | Groups | Name | Variance | SD |
| *Null model* |  |  |  |  |
|  | ParticipantID | Intercept | 1.128 | 1.062 |
|  | Residual |  | 1.132 | 1.064 |
| *Random intercepts model* |  |  |  |  |
|  | ParticipantID | Intercept | 1.124 | 1.060 |
|  | Residual |  | 1.078 | 1.038 |
| *Random intercepts model controlling for affect* |  |  |  |  |
|  | ParticipantID | Intercept | 1.0807 | 1.0396 |
|  | Residual |  | 0.9949 | 0.9974 |

| Fixed Effects |  |  |  |  |
| --- | --- | --- | --- | --- |
|  |  | *B* | SE | *t* |
| *Null model* |  |  |  |  |
|  | Intercept | 4.3637 | 0.1129 | 38.64 |
| *Random intercepts model* |  |  |  |  |
|  | Intercept | 4.55120 | 0.12470 | 36.497 |
|  | Discrimination | -0.88538 | 0.11300 | -7.835 |
|  | Family & Home Life | -0.13015 | 0.09341 | -1.393 |
|  | Finances | -0.06759 | 0.10237 | -0.660 |
|  | Mental Health | -0.15213 | 0.09790 | -1.554 |
|  | Physical Health | -0.03075 | 0.09489 | -0.324 |
|  | Romantic Relationships | -0.46928 | 0.09918 | -4.732 |
|  | Social Situations | -0.10349 | 0.09865 | -1.049 |
| *Random intercepts model controlling for affect* |  |  |  |  |
|  | Intercept | 3.15309 | 0.17181 | 18.352 |
|  | PreEMA | 0.34199 | 0.02968 | 11.522 |
|  | Discrimination | -0.96213 | 0.10880 | -8.843 |
|  | Family & Home Life | -0.18988 | 0.08991 | -2.112 |
|  | Finances | -0.15327 | 0.09865 | -1.554 |
|  | Mental Health | -0.17804 | 0.09410 | -1.892 |
|  | Physical Health | -0.09772 | 0.09137 | -1.070 |
|  | Romantic Relationships | -0.48911 | 0.09532 | -5.131 |
|  | Social Situations | -0.15865 | 0.09491 | -1.672 |

## **Table B.3.4:** *Results for Hypotheses 2a and 2b: Writing demand and affect score, Model results*

| Random Effects |  |  |  |  |
| --- | --- | --- | --- | --- |
|  | Groups | Name | Variance | SD |
| *Null model* |  |  |  |  |
|  | ParticipantID | Intercept | 0.1489 | 0.3859 |
|  | Residual |  | 0.5894 | 0.7677 |
| *Random intercepts model* |  |  |  |  |
|  | ParticipantID | Intercept | 0.1497 | 0.3869 |
|  | Residual |  | 0.5838 | 0.7640 |

| Fixed Effects |  |  |  |  |
| --- | --- | --- | --- | --- |
|  |  | *B* | SE | *t* |
| *Null model* |  |  |  |  |
|  | Intercept | 1.66138 | 0.12076 | 13.76 |
|  | PreEMA | 0.68472 | 0.02605 | 26.29 |
| *Random intercepts model* |  |  |  |  |
|  | Intercept | 1.67541 | 0.14932 | 11.220 |
|  | PreEMA | 0.68626 | 0.02596 | 26.434 |
|  | Long | -0.20649 | 0.13010 | -1.587 |
|  | One-letter | -0.03243 | 0.09956 | -0.326 |
|  | Two-letter | 0.09323 | 0.10473 | 0.890 |
|  | Write your own | -0.34725 | 0.18373 | -1.890 |

## **Table B.3.5:** *Results for Hypotheses 2a and 2b: Writing demand and emotion regulation efficacy, Model results*

| Random Effects |  |  |  |  |
| --- | --- | --- | --- | --- |
|  | Groups | Name | Variance | SD |
| *Null model* |  |  |  |  |
|  | ParticipantID | Intercept | 0.7732 | 0.8793 |
|  | Residual |  | 0.7821 | 0.8844 |
| *Random intercepts model* |  |  |  |  |
|  | ParticipantID | Intercept | 0.7635 | 0.8738 |
|  | Residual |  | 0.7791 | 0.8827 |

| Fixed Effects |  |  |  |  |
| --- | --- | --- | --- | --- |
|  |  | *B* | SE | *t* |
| *Null model* |  |  |  |  |
|  | Intercept | 4.5540 | 0.1042 | 43.72 |
| *Random intercepts model* |  |  |  |  |
|  | Intercept | 4.55929 | 0.15038 | 30.319 |
|  | Long | 0.17126 | 0.15068 | 1.137 |
|  | One-letter | -0.04992 | 0.11578 | -0.431 |
|  | Two-letter | 0.13007 | 0.12143 | 1.071 |
|  | Write your own | 0.06761 | 0.21305 | 0.317 |

## **Table B.3.5:** *Results for Hypotheses 2a and 2b: Writing demand and reappraisal efficacy, Model results*

| Random Effects |  |  |  |  |
| --- | --- | --- | --- | --- |
|  | Groups | Name | Variance | SD |
| *Null model* |  |  |  |  |
|  | ParticipantID | Intercept | 1.143 | 1.069 |
|  | Residual |  | 1.038 | 1.019 |
| *Random intercepts model* |  |  |  |  |
|  | ParticipantID | Intercept | 1.144 | 1.070 |
|  | Residual |  | 1.036 | 1.018 |

| Fixed Effects |  |  |  |  |
| --- | --- | --- | --- | --- |
|  |  | *B* | SE | *t* |
| *Null model* |  |  |  |  |
|  | Intercept | 4.380 | 0.126 | 34.77 |
| *Random intercepts model* |  |  |  |  |
|  | Intercept | 4.1703 | 0.1780 | 23.425 |
|  | Long | 0.4513 | 0.1737 | 2.598 |
|  | One-letter | 0.2043 | 0.1335 | 1.530 |
|  | Two-letter | 0.2155 | 0.1400 | 1.539 |
|  | Write your own | 0.2401 | 0.2457 | 0.977 |
