# Supplementary material for: Examining State Affective and Cognitive Outcomes Following Brief Mobile Phone-Based Training Sessions to Reduce Anxious Interpretations
Source: Cognit Ther Res. Author manuscript; Available in PMC 2026 Feb 11. (PMC12890198; doi:10.1007/s10608-025-10623-z)
Supplement: Supplementary file 3 [file NIHMS2132144-supplement-Supplement_C.docx]

**Supplemental Material C: Full Sample Follow-Up Analyses (Including Faculty)** 

Table of Contents

Descriptives & Primary Analyses with Full Sample (Faculty Included)	2

Results	2

Tables	4

Table C.1: Demographic Characteristics	4

Table C.2a: Results for Hypothesis 1a: Scenario domain and affect score, Model results	4

Table C.2b: Results for Hypothesis 1a: Scenario domain and affect score, Pairwise comparisons	6

Table C.2c: Results for Hypothesis 1a: Scenario domain and affect score, Model comparison	7

Table C.3a: Results for Hypotheses 2a and 2b: Writing demand and affect score, Model results	7

Table C.3b: Results for Hypotheses 2a and 2b: Writing demand and affect score, Pairwise comparisons with contrasts	9

Table C.3c: Results for Hypotheses 2a and 2b: Writing demand and affect score, Model comparison	9

# Descriptives & Primary Analyses with Full Sample (Faculty Included) 

## **Results**

**Sample**

Sample includes the *N =* 100 participants included in primary analyses and the *N =* 6 faculty member participants included in Supplemental Material A’s primary faculty analyses for a total sample size of *N =* 106 participants and 1,731 completed microdoses. We removed any participants/microdoses 1) who completed less than four microdoses, 2) that were missing any of our outcome variables, and 3) that had a pre-microdose affect score of 6 or 7. 

**Demographics** 

98 participants provided demographic data. Most participants identified as White/European (66.04%), non-Hispanic (88.68%), women (76.42%). See Table C.1 for full demographic information. 

**How did post-microdose affect scores (controlling for pre-microdose affect) differ based on domain? (Hypothesis 1a)**

In line with our main analysis results, post-microdose affect scores were significantly more positive for the academics/work/career development, family and home life, finances, mental health, physical health, and social situations stressor domains compared to the romantic relationships domain, and scores were significantly more positive for academics/work/career development and physical health than the discrimination domain; see Tables C.2a and C.2b for model results and pairwise comparisons. No other pairwise comparisons across domains were significant. The random intercepts model with domain as a predictor performed significantly better than the null model with no predictor (Chi-square=50.75, df=7, *P<*.001). See Table C.2c for full model comparison, including Akaike Information Criterion (AIC) values. 

**How did post-microdose affect scores (controlling for pre-microdose affect) differ based on writing demand? (Hypotheses 2a and 2b)**

	Writing demand data was collected (and thus analyzed) for 87 (out of 106) participants, including 1,247 (out of 1,731) microdoses.

Consistent with main analysis results, both scenarios with no writing (one- and two-letters missing; *B=*0.367, *SE=*.09, *P=*.0001) and some writing (fill-in-the-blank scenarios; *B=*0.349, *SE=*.12, *P=*0.004) were associated with significantly more positive post-microdose affect than scenarios containing a large amount of writing (write your own and long scenarios). There was no significant difference between post-microdose affect scores for scenarios with no writing (one- and two-letters missing) and fill-in-the-blank scenarios (*B=*0.02, *SE=*.09, *P=*.84). See Tables C.3a and C.3b for model results and pairwise comparisons. The random intercepts model with writing demand as a predictor performed significantly better than the null model with no predictor (Chi-square=14.30, df=4, *P*=.006). See Table C.3c for full model comparison. 

## **Tables**

| **Table C.1:** *Demographic Characteristics* |  |
| --- | --- |
| Characteristic | *n* (%) |
| Gender: Woman Man Transgender Man Transgender Woman Other identity Not Reported Race: White/European Origin East Asian Other or Unknown South Asian Black/African Origin Participant selected more than one race American Indian/Alaska Native Native Hawaiian/Pacific Islander Ethnicity: Not Hispanic or Latino Hispanic or Latino | 81 (76.42%) 15 (14.15%) 0 (0%) 0 (0%) 1 (0.94%) 0 (0%) 70 (66.04%) 8 (7.55%) 7 (6.60%) 6 (5.66%) 4 (3.77%) 3 (2.83%) 0 (0%) 0 (0%) 94 (88.68%) 4 (3.77%) |

### **Table C.2a:** *Results for Hypothesis 1a: Scenario domain and affect score, Model results*

| Random Effects |  |  |  |  |
| --- | --- | --- | --- | --- |
|  | Groups | Name | Variance | SD |
| *Null model* |  |  |  |  |
|  | ParticipantID | Intercept | 0.1472 | 0.3837 |
|  | Residual |  | 0.5602 | 0.7484 |
| *Random intercepts model* |  |  |  |  |
|  | ParticipantID | Intercept | 0.1447 | 0.3804 |
|  | Residual |  | 0.5430 | 0.7369 |

| Fixed Effects |  |  |  |  |
| --- | --- | --- | --- | --- |
|  |  | *B* | SE | *t* |
| *Null model* |  |  |  |  |
|  | Intercept | 1.72931 | 0.10035 | 17.23 |
|  | PreEMA | 0.66665 | 0.02133 | 31.25 |
| *Random intercepts model* |  |  |  |  |
|  | Intercept | 1.83192 | 0.10400 | 17.615 |
|  | PreEMA | 0.66409 | 0.02110 | 31.466 |
|  | Discrimination | -0.27387 | 0.07837 | -3.494 |
|  | Family & Home Life | -0.05501 | 0.06378 | -0.863 |
|  | Finances | -0.04838 | 0.07156 | -0.676 |
|  | Mental Health | -0.10073 | 0.06645 | -1.516 |
|  | Physical Health | 0.07109 | 0.06579 | 1.081 |
|  | Romantic Relationships | -0.41699 | 0.06912 | -6.033 |
|  | Social Situations | -0.04578 | 0.06808 | -0.673 |

### **Table C.2b:** *Results for Hypothesis 1a: Scenario domain and affect score, Pairwise comparisons* 

| Domain |  | *B* | SE | df | *t* | *P* |
| --- | --- | --- | --- | --- | --- | --- |
| Academics/Work/Career | Discrimination | 0.27387 | 0.0784 | 1703 | 3.492 | **0.0103** |
|  | Family/Home | 0.05501 | 0.0638 | 1675 | 0.862 | 1.0000 |
|  | Finances | 0.04838 | 0.0716 | 1664 | 0.676 | 1.0000 |
|  | Mental | 0.10073 | 0.0665 | 1671 | 1.515 | 1.0000 |
|  | Physical | -0.07109 | 0.0658 | 1669 | -1.080 | 1.0000 |
|  | Romantic | 0.41699 | 0.0692 | 1682 | 6.030 | **<.0001** |
|  | Social Situations | 0.04578 | 0.0681 | 1663 | 0.672 | 1.0000 |
| Discrimination | Family/Home | -0.21886 | 0.0817 | 1696 | -2.680 | 0.1485 |
|  | Finances | -0.22550 | 0.0880 | 1695 | -2.562 | 0.1887 |
|  | Mental | -0.17315 | 0.0843 | 1699 | -2.055 | 0.6404 |
|  | Physical | -0.34497 | 0.0827 | 1688 | -4.170 | **0.0007** |
|  | Romantic | 0.14312 | 0.0862 | 1700 | 1.661 | 1.0000 |
|  | Social Situations | -0.22809 | 0.0856 | 1697 | -2.665 | 0.1485 |
| Family & Home Life | Finances | -0.00663 | 0.0752 | 1664 | -0.088 | 1.0000 |
|  | Mental | 0.04572 | 0.0698 | 1661 | 0.655 | 1.0000 |
|  | Physical | -0.12610 | 0.0698 | 1665 | -1.807 | 1.0000 |
|  | Romantic | 0.36198 | 0.0730 | 1678 | 4.957 | **<.0001** |
|  | Social Situations | -0.00923 | 0.0719 | 1667 | -0.128 | 1.0000 |
| Finances | Mental | 0.05235 | 0.0779 | 1669 | 0.672 | 1.0000 |
|  | Physical | -0.11947 | 0.0769 | 1663 | -1.553 | 1.0000 |
|  | Romantic | 0.36861 | 0.0794 | 1666 | 4.640 | **0.0001** |
|  | Social Situations | -0.00259 | 0.0791 | 1665 | -0.033 | 1.0000 |
| Mental Health | Physical | -0.17182 | 0.0727 | 1662 | -2.365 | 0.3084 |
|  | Romantic | 0.31626 | 0.0754 | 1674 | 4.193 | **0.0007** |
|  | Social Situations | -0.05494 | 0.0748 | 1662 | -0.734 | 1.0000 |
| Physical Health | Romantic | 0.48809 | 0.0744 | 1666 | 6.563 | **<.0001** |
|  | Social Situations | 0.11688 | 0.0740 | 1665 | 1.580 | 1.0000 |
| Romantic Relationships | Social Situations | -0.37121 | 0.0767 | 1671 | -4.838 | **<.0001** |

### **Table C.2c:** *Results for Hypothesis 1a: Scenario domain and affect score, Model comparison*

|  | *npar* | AIC | BIC | Log Likelihood | Deviance | Chi-Square | Df | *P* |
| --- | --- | --- | --- | --- | --- | --- | --- | --- |
| Baseline | 4 | 4062.1 | 4083.9 | -2027.1 | 4054.1 |  |  |  |
| Random effects | 11 | 4016.4 | 4076.4 | -1997.2 | 3994.4 | 59.751 | 7 | <.0001 |

### **Table C.3a:** *Results for Hypotheses 2a and 2b: Writing demand and affect score, Model results*

| Random Effects |  |  |  |  |
| --- | --- | --- | --- | --- |
|  | Groups | Name | Variance | SD |
| *Null model* |  |  |  |  |
|  | ParticipantID | Intercept | 0.1567 | 0.3958 |
|  | Residual |  | 0.5916 | 0.7692 |
| *Random intercepts model* |  |  |  |  |
|  | ParticipantID | Intercept | 0.1562 | 0.3952 |
|  | Residual |  | 0.5846 | 0.7646 |

| Fixed Effects |  |  |  |  |
| --- | --- | --- | --- | --- |
|  |  | *B* | SE | *t* |
| *Null model* |  |  |  |  |
|  | Intercept | 1.65647 | 0.11861 | 13.96 |
|  | PreEMA | 0.68258 | 0.02553 | 26.74 |

| *Random intercepts model* |  |  |  |  |
| --- | --- | --- | --- | --- |
|  | Intercept | 1.67434 | 0.14549 | 11.508 |
|  | PreEMA | 0.68459 | 0.02541 | 26.946 |
|  | Long | -0.18398 | 0.12447 | -1.478 |
|  | One-letter | -0.03027 | 0.09477 | -0.319 |
|  | Two-letter | 0.06626 | 0.09987 | 0.664 |
|  | Write your own | -0.51444 | 0.16873 | -3.049 |

### **Table C.3b:** *Results for Hypotheses 2a and 2b: Writing demand and affect score, Pairwise comparisons with contrasts*

| Contrast | *B* | SE | df | *t* | *P* |
| --- | --- | --- | --- | --- | --- |
| No writing - Fill-in-the-blank | -0.018 | 0.0933 | 1193 | 0.193 | 0.8470 |
| No writing - More writing | 0.367 | 0.0906 | 1202 | 4.052 | **0.0001** |
| Fill-in-the-blank - More writing | 0.349 | 0.1210 | 1178 | 2.880 | **0.0040** |

### **Table C.3c:** *Results for Hypotheses 2a and 2b: Writing demand and affect score, Model comparison* 

|  | *npar* | AIC | BIC | Log Likelihood | Deviance | Chi-Square | Df | *P* |
| --- | --- | --- | --- | --- | --- | --- | --- | --- |
| Baseline | 4 | 3009.7 | 3030.2 | -1500.9 | 3001.7 |  |  |  |
| Random effects | 8 | 2999.3 | 3040.3 | -1491.7 | 2983.3 | 18.406 | 4 | **0.001** |
